# Role of 2‑Hydroxyimines in Chiral Phosphoric Acid-Catalyzed Mannich-Type Reactions: Enhancing Reactivity and Selectivity via Dimerization

**DOI:** 10.1021/jacs.5c22497

**Published:** 2026-05-06

**Authors:** Markus Hecht, Hendrik Fischer, Wagner Silva, Verena Eichstetter, Christian L. Scholtes, Ana Sofia Ferreira, Eurico J. Cabrita, Dominik Horinek, Ruth M. Gschwind

**Affiliations:** † Institute of Organic Chemistry, 9147 University Regensburg, Regensburg 93053, Germany; ‡ Institute of Physical and Theoretical Chemistry, 9147 University Regensburg, Regensburg 93053, Germany; § UCIBIO, Faculdade de Ciências e Tecnologia, Universidade Nova de Lisboa, Caparica 2829-516, Portugal

## Abstract

Chiral phosphoric acids (CPAs) have emerged as versatile catalysts for asymmetric catalysis, capable of transforming a wide selection of substrates with high stereoselectivities. However, the mechanistic role of higher aggregates in CPA-catalyzed reactions remains poorly understood, although increasing evidence suggests that dimeric and trimeric CPA species can promote challenging transformations. This work provides comprehensive experimental evidence demonstrating that special [CPA/imine]_2_ species critically enhance the reactivity and selectivity in CPA-catalyzed Mannich-type reactions with imines bearing an *N*-2-hydroxyphenyl moiety. Using low-temperature NMR spectroscopy, diffusion-ordered spectroscopy (DOSY), and molecular dynamics (MD) simulations, we revealed that imines with a *N*-2-hydroxyphenyl moiety promote the formation of dimeric [CPA/imine]_2_ aggregates, while monomeric CPA/imine complexes dominate, with imines lacking this moiety. [CPA/imine]_2_ formation is favored under low-temperature and high-concentration conditions. Dimers with sufficient structural flexibility provide enhanced reactivity, acidity, and selectivity. In contrast, at higher temperatures, where no [CPA/imine]_2_ aggregates are formed, the Mannich-type reaction proceeds inefficiently. A nonlinear effect analysis provided evidence of asymmetric amplification in the present Mannich-type reaction, proving the participation of aggregated species in the reaction pathway. Together, these results highlight the importance of controlling catalyst aggregation as a strategy to optimize the reactivity and selectivity in asymmetric organocatalysis.

## Graphical abstract

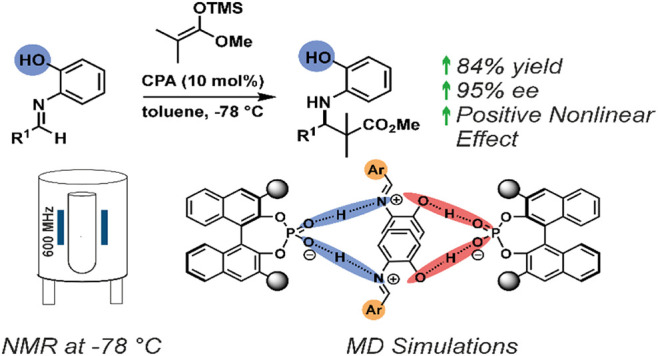

## Introduction

Since first reports by Akiyama and Terada in 2004, chiral Brønsted acids have become a highly valuable class of catalysts, adaptable to a wide range of asymmetric transformations.^1–5^ These catalysts create a stereoinductive environment around the substrate through hydrogen-bonding, Coulombic forces, and other noncovalent interactions enabling highly enantioselective transformations.^6–9^ Specifically, 1,1′-bi-2-naphthol (BINOL)-derived chiral phosphoric acids (CPAs) represent a class of catalysts known for their high enantioselectivity.^10,11^ The dual functionality of CPAs as both hydrogen bond acceptors and donors is recognized to be fundamental to the “three-point interaction model”, which is crucial for achieving high stereoselectivity.^12–15^ Furthermore, by incorporating both hydrogen bond acceptor and donor functionalities into the substrate, bidentate binding between catalyst and substrate can lead to a rigid preorganization, creating a highly confined structural space.^10,16–20^ Exemplarily, the group of Akiyama employed such a bidentate structural motif in a CPA-catalyzed Mannich-type reaction by introducing an *ortho*-hydroxy group in the imine substrate.^5^ The *N*-2-hydroxyphenyl group of the aldimine was found to be essential for the present Mannich-type reaction at low temperature (−78 °C) and their computational study revealed a transition state, in which the substrate is anchored by two hydrogen bonds (Figure 1).^10^ In contrast, NMR studies of these CPA and *N*-2-hydroxyphenyl imines indicated an intermolecular bidentate substrate binding and revealed a structural distribution composed of three dominant species of [CPA/imine]_2_ dimers (Figure 1).^21^ Molecular dynamics (MD) simulations revealed that in these [CPA/imine]_2_ dimers, two imines effectively bridge two CPA molecules, indicating that the respective transformation in the Mannich-type reaction might proceed via a dimeric reaction pathway rather than through the monomeric bidentate CPA/imine complex. However, to the best of our knowledge, experimental insights into potential monomeric or dimeric reaction pathways of CPA-catalyzed reactions remain limited, despite increasing evidence suggesting the presence of higher aggregates in CPA-catalyzed reactions.^21–25^ For example, the group of Hunger demonstrated that diphenyl phosphoric acids form a multimer with quinaldine,^26^ while Detering and colleagues identified phosphoric acid trimers in reaction solutions.^27^ Understanding and controlling such aggregation processes is of paramount importance as it would not only enable deeper insights into organocatalytic mechanisms but also unlock new opportunities in catalysis, when such catalyst aggregates can be designed and applied in a controlled fashion.^28^ Therefore, this report presents a comprehensive experimental study on the impact of temperature, catalyst loading, and nonlinear effects in the Mannich-type reaction.^29–31^

**Figure 1:**
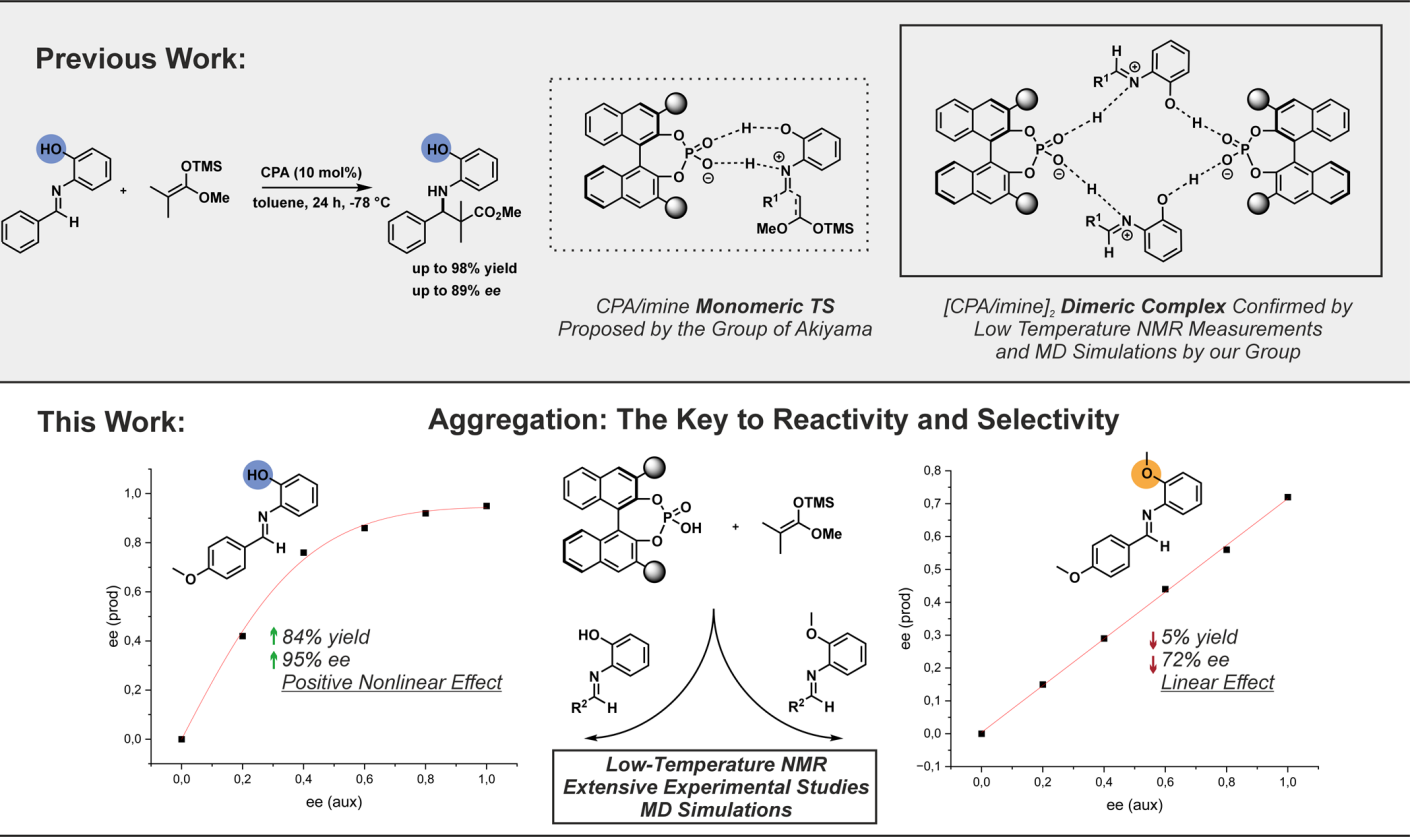
Monomeric vs Dimeric Reaction Pathway: CPA-catalyzed Mannich-type reaction developed by the group of Akiyama,^5,10^ yielding β-amino esters with high yields and enantioselectivities. Extensive NMR studies by our group confirmed the bidentate substrate binding proposed by the Akiyama group but revealed a broad structural space, favoring [CPA/imine]_2_ dimers over the proposed monomeric complex by the group of Akiyama. This study investigates the effects of temperature, catalyst loading, and nonlinear effects in the Mannich-type reaction, providing evidence for the involvement of flexible CPA aggregates, supported by low-temperature NMR and MD simulations.

Supported by low-temperature NMR spectroscopy, kinetics, and MD simulations, the study examines chiral phosphoric acids (CPAs) and *N*-Triflylphosphoramides (NTPAs) with a *N*-(*ortho*-hydroxyaryl) imine, *N*-(*ortho*-methoxyaryl) imine, *N*-(para-hydroxyaryl) imine, and unsubstituted imine (9 acid/imine combinations), highlighting the crucial role of the *N*-2-hydroxyphenyl group in the aldimine for the reaction. Both the synthetic study and the low-temperature NMR measurements, along with computer simulations, corroborated the unique aggregation behavior of CPAs and provided evidence for the involvement of flexible CPA dimers in reactivity and stereoselectivity in the Mannich-type reaction. In contrast, no such aggregation was observed for the NTPA system, supporting our previous findings.^32^

## Results and Discussion

### Model Systems

CPA catalyst **1a**, bearing a 4-NO_2_C_6_H_4_ residue at the 3,3′-position, was selected based on Akiyama’s model system^5^ and because of its lack of prior spectroscopic investigation (Figure 2). In parallel, CPA catalyst **1b**, featuring 3,5-CF_3_C_6_H_3_ substituents at the 3,3′-position, was included due to its comparable acidity, its unexplored reactivity in the Mannich-type reaction, and additional NMR insights through ^19^F NMR investigations. In addition, the more acidic NTPA catalyst **1c** was studied to compare the aggregation behavior of CPAs and NTPAs, as well as to evaluate the reactivity and selectivity of the NTPA complex relative to the aggregated CPA species, as the NTPA catalyst is employed in Mannich-type reactions of imines lacking the *N*-2-hydroxyphenyl moiety.^16^ Moreover, CPA catalyst **1d** was included, as it had previously been used for the spectral elucidation of the [CPA/imine]_2_ dimers and serves in this study as a reference for the dimer specification (Figure 2).^33^ For the imines **2a**–**d**, a para-methoxy residue was selected as a potential probe for structural investigations via NOESY and DOSY NMR as it is well separated from the crowded aromatic region (Figure 2). The *N*-*ortho* substituent was systematically varied to demonstrate the origin of the aggregation behavior and its influence on reaction yields, stereoselective outcomes, and nonlinear effects. All NMR measurements were carried out at −78 °C to simulate reaction conditions developed by the group of Akiyama,^5,10^ slow down potential exchange processes, and achieve optimally resolved hydrogen bond patterns. In total, 16 samples with various acid/imine combinations and concentrations were analyzed. In the previous work,^5,10^ toluene was used as the solvent, as it yielded the best yield and stereoselectivity in the reaction. However, based on our previous studies,^21,34^ low-temperature ^1^H NMR spectra in toluene resulted in very broad signals making them unsuitable for further investigations. Hence, CD_2_Cl_2_ was selected as the solvent for NMR measurements, as it offered the best chemical shift dispersion and line widths (Figure 3). In addition, previous studies of acid/imine complexes were successfully conducted in CD_2_Cl_2_ in our research group,^34–36^ and nonlinear effects for the Mannich-type reaction in this study were investigated in CD_2_Cl_2_ as well as in toluene (Figure 5). To investigate the structural space and check higher aggregate formation in the model systems, catalysts **1a**–**d** were initially studied in 1:1 catalyst/imine samples with imine **2a** (Figure 3A), which has the essential *N*-2-hydroxyphenyl moiety and previously formed dimeric species with CPAs.^21^ Simultaneously, we also analyzed reaction condition samples with a 1:10 catalyst/imine ratio (10:100 mM). Similar hydrogen-bond patterns were observed in the ^1^H spectra, along with comparable ^19^F and ^31^P patterns (Figures S2–S7, S20–S25, and S38–41). However, 1:1 catalyst/imine samples were used for more detailed investigations due to better signal intensities. In the **1a/2a** and **1b/2a** system, various signals were observed in the H-bond region (Figure 3A). Previous studies by our group focusing on the structural space of 19 other CPA/imine combinations assigned signals at 14 ppm to POHN hydrogen bonds of the dimeric species based on scalar couplings and ^1^H–^1^H COSY spectra, while signals at 12 ppm were attributed to POHO hydrogen bonds based on cross-signals in the ^1^H–^31^P HMBC spectrum.^21^ Furthermore, the ^1^H NMR spectrum of **1d/2a** was reproduced in this study. **1d/2a** was previously used as the model system for the structural elucidation of [CPA/imine]_2_ dimer and serves here as the basis for the H-bond interpretations of our highly reactive and selective complexes.^21^ In **1d/2a**, the very sharp H-bond signals allowed for an in-depth structural assignment. Moreover, the sharp signals indicate reduced chemical exchange between the dimeric structures and reduced flexibility within the dimers compared with the **1a/2a** and **1b/2a** complexes.

**Figure 2:**
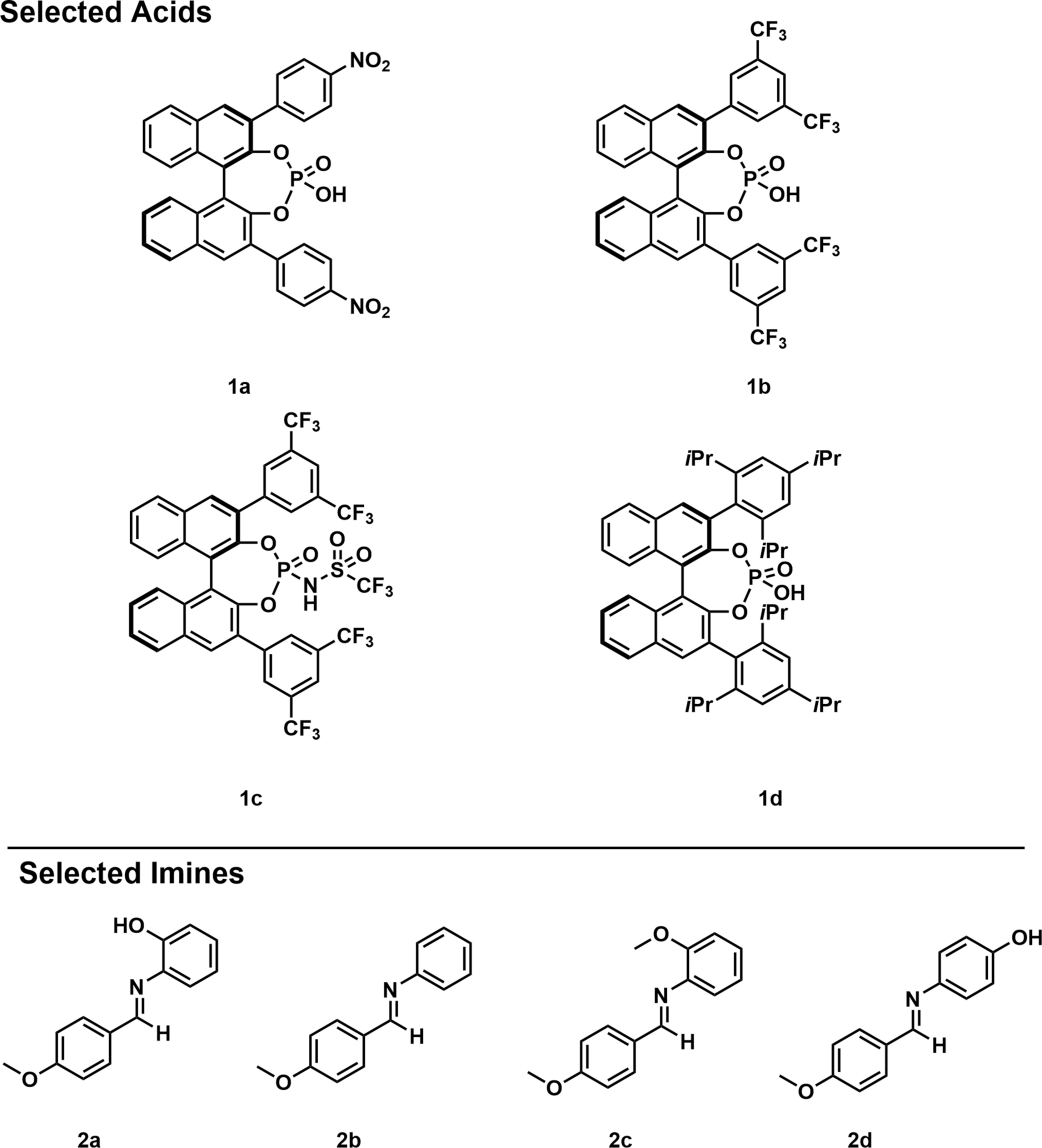
Structures of the investigated chiral phosphoric acids (CPAs) **1a/1b/1d**, *N*-Triflylphosphoramides (NTPAs) **1c**, and imines **2a**–**d**. **1a** was selected based on Akiyama’s model system, and catalysts **1b**, **1c**, and **1d** were added to enable ^19^F NMR investigations, to compare the aggregation behavior of CPAs and NTPAs and as a reference for the dimer specification, respectively. For the imines **2a**–**d**, a *para*-methoxy residue was selected as a potential probe for structural NMR investigations and the *N*-*ortho* substituent was varied to demonstrate the origin of the aggregation behavior and its influence.

**Figure 3:**
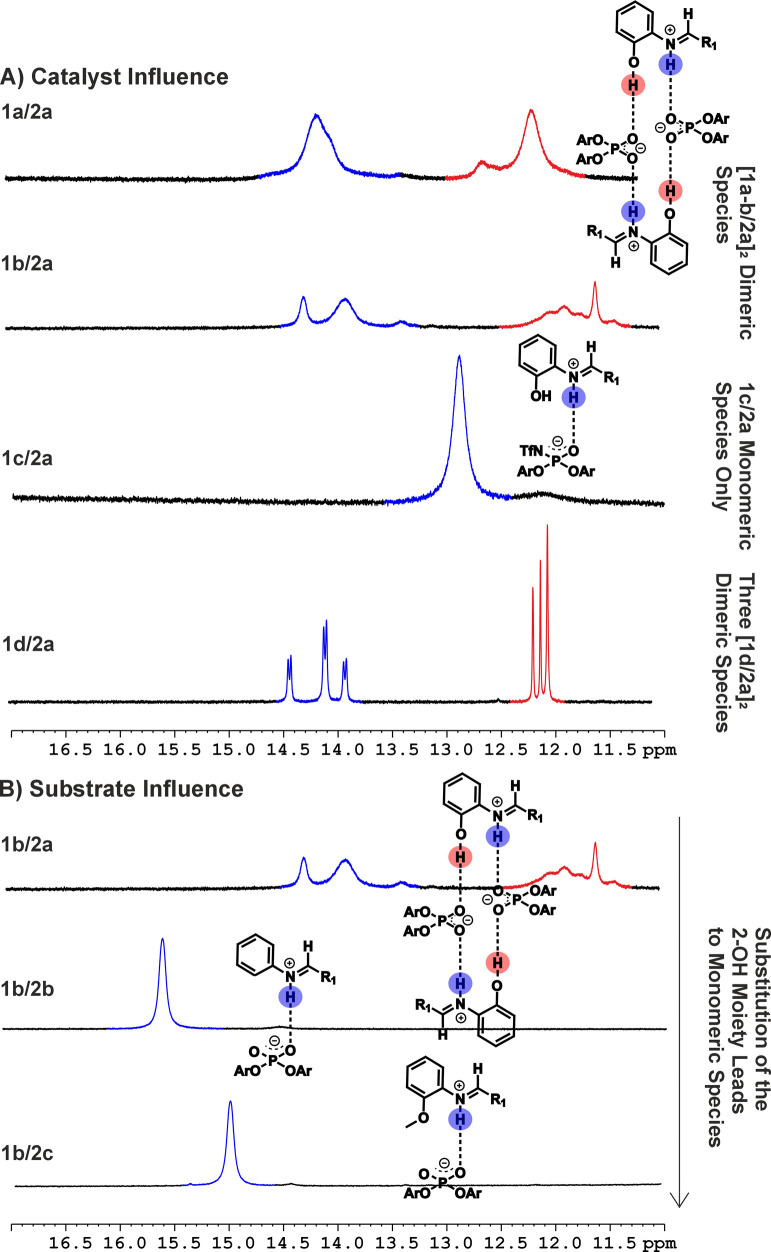
A) H-Bond section of the ^1^H NMR spectra of **1a–d/2a** at a 1:1 ratio and a concentration of 10 mM in CD_2_Cl_2_ at 600 MHz at −78 °C. For **1a/2a** and **1b/2a**, distinct species were observed previously identified as [CPA/imine]_2_ dimers with the **1d/2a** combination (reproduced in this study),^21^ while for **1c/2a**, only a monomeric binary complex was obtained. B) H-Bond section of the ^1^H NMR spectra of **1b/2a** (top), **1b/2b** (middle), and **1b/2c** (bottom) at a 1:1 ratio and a concentration of 10 mM in CD_2_Cl_2_ at 600 MHz at −78 °C. As soon as the crucial *N*-2-hydroxyphenyl moiety is substituted in the **1b/2b** and **1b/2c** system, only a monomeric binary complex could be identified. Focusing on the previously unexplored Mannich-type reaction catalyzed by CPA **1b**, the **1b/2a–c** systems are an ideal model to study the influence of aggregation on reactivity and selectivity and to shed light on why the *N*-2-hydroxyphenyl moiety is so crucial for the reaction.

Although the spectra of the reactive **1a/2a** system and the yet unexplored **1b/2a** system in the Mannich-type reaction appear rather broad, the H-Bond region of the ^1^H and the ^19^F/^31^P spectra (Figures S2–S7, S38–S41) clearly confirms the presence of several coexisting dimeric species, making them valuable for detailed investigations regarding reactivity and selectivity. In contrast, for the stronger chiral Brønsted acid systems **1c/2a**, only a single H-bond is observed in the ^1^H spectra, indicating only a monomeric complex between the acid and imine (Figure 3A). The ^19^F and ^31^P spectra also confirm the presence of only a monomeric complex (Figures S21, S22, S24 and S25), supporting our previous studies showing that NTPAs do not form higher aggregates.^32^ However, what is even more interesting is that the hydrogen-bonded protons in the aggregated CPA/imine complexes **1a/2a** and **1b/2a** shift to ppm values comparable to those observed for the more acidic NTPA/imine complex **1c/2a**. It is well established that the chemical shift of the H-bond proton directly reflects the acidity of the system. Indeed, previous studies from our group have extensively focused on CPA/imine complexes and demonstrated through a Steiner–Limbach correlation^37–39^ a direct correlation between the ^1^H chemical shift and internal acidity in these systems, which in turn correlates with higher reactivity.^40,41^ This finding also explains why the *N*-2-hydroxyphenyl group of the aldimine is essential for the reactivity observed in the present Mannich-type reaction previously catalyzed by CPA **1a**, as it enhances the internal acidity of the system. To gain more detailed structural insights into the role of the *N*-2-hydroxyphenyl moiety of the imine and the aggregation behavior, we next investigated 1:1 and 1:10 samples of catalyst **1b** with imines **2a**–**d** (Figures S8–S19, S42). CPA **1b** was selected due to its superior spectral resolution compared to CPA **1a** and because of its unknown reactivity in the Mannich-type reaction. Indeed, when the *N*-2-hydroxyphenyl moiety in the imine is substituted (**2b**/**2c**), only a single peak is readily apparent in the H-bond region, indicating only a monomeric complex between CPA and the imine (Figure 3B). Similarly, in the ^19^F and ^31^P spectra, only a monomeric complex is observed. In addition, we shifted the crucial hydroxy group to the 4-position using imine **2d**, as the group of Akiyama previously reported strongly reduced enantioselectivity and yield for the reaction with **2d**.^10^ Also in our study, we observed a strong *ortho* versus *para* effect. In the **1** **b** **/2d** complex, the POHO hydrogen bond signal is about 2 ppm high-field shifted indicating in average weaker POHO hydrogen bonds (Figure S42). However, **2d** alone as well as **1b/2d** complexes showed tremendously reduced solubilities in both CD_2_Cl_2_ and toluene, preventing any further investigations.

Still, what is even more intriguing is the downfield shift of the hydrogen-bonded protons in the monomeric complexes **1b/2b** and **1b/2c** relative to the aggregated CPA/imine complexes **1b/2a**, indicating again reduced internal acidity. As demonstrated in previous work by the group of Akiyama,^5,10^ the Mannich-type reaction catalyzed by CPA **1a** proceeded effectively only with the *N*-2-hydroxyphenyl moiety, where we identified [CPA/imine]_2_ dimers. Substitution or relocation of this moiety to the 4-position led to a decrease in reactivity and stereoselectivity.^10^

### Temperature, Catalyst Loading, and Substituents Affecting Reactivity, Selectivity, and Complex Structure

Since the NMR spectra show multiple [CPA/imine]_2_ species for the **1b/2a** system, possible aggregates for the poorly soluble **1b/2d** system, and only monomeric complexes for the **1b/2b–c** systems, we first aimed to determine if the reactivity and stereoselectivity trends observed in previous studies with CPA **1a** ^5,10^ align with our findings in the **1b/2a–d** systems. Therefore, we examined CPA **1b** in the Mannich-type reaction with the imines **2a**–**d** (Table 1). We selected the same reaction conditions previously reported by the group of Akiyama, using a temperature of −78 °C and a catalyst loading of 10% in toluene.^5,10^ In addition, the **1a/2a** and **1c/2a** combinations were included, and all reactions with CPA **1b** were also performed in dichloromethane at −78 °C with a catalyst loading of 10% (Table 1). Indeed, the same interesting effect of the *N*-aryl group of the aldimine previously reported with CPA **1a** was found in our systems.

**Table 1:**
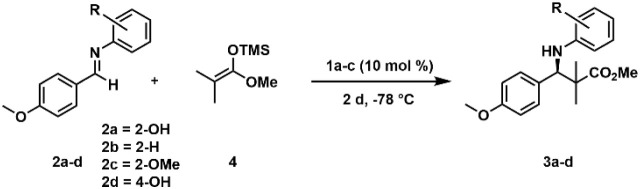
CPAs **1a**–**c** Were Examined in the Mannich-Type Reaction with Imines **2a**–**2d** to Address the Importance of the *N*-2-Hydroxyphenyl Moiety^a^

Entry	Imine	Catalyst	Solvent	Aggregates	*ee*%	Yield [%]
1	2a	1a	toluene	yes	75	86 ^10^
2	2a	1b	toluene	yes	95	84
3	2a	1c	toluene	no	43	89
4	2b	1b	toluene	no	50	20
5	2c	1b	toluene	no	72	5
6	2d	1b	toluene	n.d.	-	-
7	2a	1b	CD_2_Cl_2_	yes	81	71
8	2b	1b	CD_2_Cl_2_	no	39	37
9	2c	1b	CD_2_Cl_2_	no	41	35
10	2d	1b	CD_2_Cl_2_	n.d.	-	-
11	2a	1d	toluene	sharp	-	-

a Temperature of −78 °C and 10 mol % were chosen to match the reaction conditions from previous work. Aggregates with broad NMR H-bond signals indicating flexible structures show high reactivity. Aggregates with sharp H-bond signals assigned to closed dimers show no reactivity (entry 11).

Imine **2a** with the *N*-2-hydroxyphenyl moiety exhibited remarkably superior reaction yields up to 84% and enantioselectivity of *ee*% = 95% compared to imines **2b**, **2c**, and **2d** with CPA **1b** in both CD_2_Cl_2_ and toluene (Table 1, entries 2 and 7). Moreover, for imine **2a**, both yield and stereoselectivity were enhanced using CPA **1b**, compared to values previously reported by Akiyama for CPA **1a** (yields up to 86%, *ee*% = 75%).^10^ NTPA catalyst **1c**, which does not form aggregates but has comparable acidity according to the H-bond analysis, exhibits good reactivity but only poor selectivity (yields up to 89%, *ee*% = 43%). When the *N*-2-substituent is changed from R = OH to R = H or R = OCH_3_ with CPA **1b**, the yield drops to 20% and 5%, respectively, while the enantioselectivity decreases to *ee*% = 50% and *ee*% = 72% (Table 1). Unfortunately, no reaction was observed when CPA **1b** was applied with imine **2d**, likely due to the very poor solubility of imine **2d**. To our surprise, CPA catalyst **1d**, which has been our model system for NMR studies due to its very sharp H-bond signals, did not show any reactivity. This hints that for an effective transformation, a certain flexibility of the dimeric structure seems to be essential.

Overall, the reduced reactivity of the Mannich-type reaction with imines lacking the *N*-2-hydroxyphenyl moiety is attributed to the formation of only monomeric CPA/imine complexes, as previously identified by NMR spectroscopy (Figure 3), resulting in lower internal acidity and efficiency of the reaction. This is particularly interesting because when these aggregation processes occur, they can change the electronic environment of the active site, which in turn can significantly influence the catalyst’s activity. For example, studies by the groups of List and Thiel showed that the heterodimerization of CPAs with small carboxylic acids increased CPA acidity, leading to higher reaction rates for epoxide ring openings.^25^ In the present case, the change in the catalytic environment is substrate-driven, mediated by the *N*-2-hydroxyphenyl moiety of imine **2a**, rather than occurring from catalyst acid–acid interactions. The substrate effects seem to be a real mechanistic advantage since substrate-driven dimerization is required for an efficient catalysis, whereas catalyst dimerization typically gives rise to competing pathways often resulting in diminished stereoselectivity. Following, our results indicate that the additional hydrogen bonding of the *N*-2-hydroxyphenyl group and therefore the identified [CPA/imine]_2_ dimers via low-temperature NMR spectroscopy may significantly amplify reactivity and selectivity given that the dimer has sufficient structural flexibility. Therefore, we aimed to study the **1b/2a** system in more detail by varying the temperature and catalyst loading in the present Mannich-type reaction, an approach that has not yet been explored within this system. Previous studies of other CPA-catalyzed reactions have shown that these parameters influence the formation of higher aggregates, potentially affecting both reactivity and stereoselectivity.^42–44^ For example, Jansen and coworkers demonstrated that in a CPA-catalyzed transfer hydrogenation of quinolines, the reaction shows a concentration-dependent change in reaction mechanism, involving either one or two catalyst molecules and therefore influencing reactivity and selectivity.^42^ Research by the group of Collum observed that temperature influenced the aggregation states of enolates, which affected the stereochemistry and the mechanism of the aldol additions.^45^ This is also particularly important because we can detect the higher aggregates in the CPA/imine system via NMR only at lower temperatures, which align with the reaction conditions. Hence, we studied CPA **1b** in the Mannich-type reaction with the imine **2a** using temperatures ranging from −78 to +80 °C and varied catalyst loadings from 1% to 25% (Table 2). When the reaction was carried out at room temperature or +80 °C, we were not able to detect any conversion to the desired product. Reducing the temperature to −10 °C, we began to observe product formation with a 10% catalyst loading, yielding 15% of the product (Table 2). As the catalyst loading increased, product formation also increased, reaching 44% with 25% catalyst loading. Finally, at −78 °C, we observed optimal reactivity and selectivity with both 10% and 25% catalyst loadings, yielding the product with high stereoselectivities up to *ee*% = 95% and yields up to 84%. The absolute stereochemistry of **3a** was determined by chiral HPLC analysis by comparison of the retention time with that found in the literature.^16^ The major isomer formed in the Mannich-type reaction with CPA **1b** (Table 2) is *S*-**3a** across all experiments.

**Table 2:**
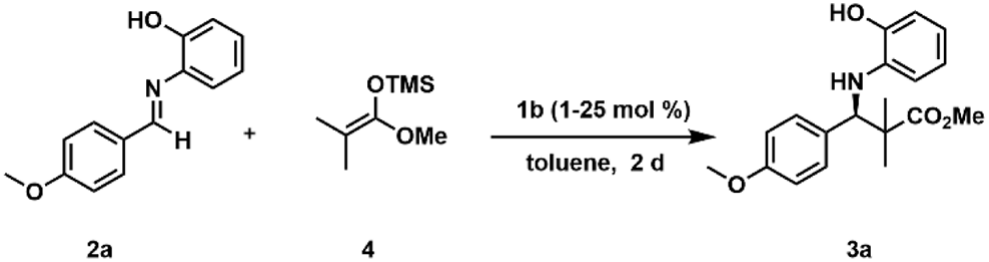
CPA **1b** Was Examined in the Mannich-Type Reaction with Imine **2a** to Address the Importance of the Temperature and Catalyst Loading^a^

Entry	Temperature [°C]	Cat. Loading [%]	*ee*%	Yield [%]
1	r.t.	1	-	n.c.
2	r.t.	10	-	n.c.
3	r.t.	25	-	n.c.
4	+80	1	-	n.c.
5	+80	10	-	n.c.
6	+80	25	-	n.c.
7	–10	1	-	n.c.
8	–10	10	89	15
9	–10	25	85	44
10	–78	1	-	n.c.
11	–78	10	95	84
12	–78	25	95	82

a Temperatures ranging from −78 °C to +80 °C and catalyst loadings from 1% to 25% were chosen to investigate the aggregation influence. No conversion (N.C.) of imine **2a** was observed at room temperature and +80 °C, and decomposition of the silyl acetal **4** was detected after 48 h.

While the rise in reactivity between −10 and −78 °C can be attributed to the formation of aggregated complexes (Figure 4), the observed difference in selectivity can be rationalized by the presence and differing reactivity of a flexible [CPA/imine]_2_ dimer, which is later explained in the nonlinear analysis and the MD simulations. Once again, this strongly suggests that the identified [CPA/imine]_2_ dimers, specifically at low temperatures, play a key role in enhancing both reactivity and selectivity. For this purpose, we focused more intensively on the temperature range between +25 °C and −10 °C, as the reaction begins to proceed within this range. NMR spectroscopy and DOSY measurements are valuable tools for investigating the proposed temperature-dependent aggregation behavior. At −80 °C, that means in the slow exchange regime, the range of volumes expected from our previous investigations is up to ∼2800 Å^3^ for a binary CPA/imine complex and up to ∼6000 Å^3^ for [CPA/imine]_2_ dimers.^21^ From room temperature down to −20 °C, free imine, binary CPA/imine complex, and [CPA/imine]_2_ dimers exchange fast on the NMR time scale. Therefore, the volumes presented in Figure 4B represent the average of all populated species and are smaller than those at −80 °C. First, we studied the **1b/2a** system at five different temperatures ranging from +25 °C to −20 °C at a 1:1 ratio of **1b** and **2a** (Figure 4). Reducing temperature, the ^1^H signals of the POHN and POHO hydrogen bonds within the [CPA/imine]_2_ dimers appear and sharpen, as the lifetime of the H-bonds progressively prolongs (Figure 4A). Moreover, looking into the temperature-dependent DOSY measurements, an increase in the molecular volume from 2800 Å^3^ at room temperature to 4600 Å^3^ at −20 °C can be observed. This shows a strongly increased population of [CPA/imine]_2_ dimers at lower temperatures for **1b/2a** (Figure 4B). In contrast, when switching to the **1b**/**2b**–**c** systems, which lack the crucial *N*-2-hydroxyphenyl moiety, only a slight increase in volume was observed, confirming the absence of [CPA/imine]_2_ dimers and explaining again the reduced reactivity in the Mannich-type reaction (Table 1). Interestingly, the volume of **1b/2a** compared to that of the **1b/2b–c** complexes is considerably higher even at room temperature, indicating dimer contributions. This result, in combination with the missing reactivity at room temperature, hints at a certain lifetime of the dimers/H-bonds to be necessary for an effective reaction. At lower catalyst loadings, the volumes using **1b/2a** are still slightly increased compared to **1b/2b–c** at room temperature (∼1400 Å^3^) and increase to 3800 Å^3^ at −20 °C. Thus, also under catalytic conditions, a significant dimer formation is observed. Overall, these results demonstrate once more why the reaction proceeds efficiently only at lower temperatures and higher catalyst loadings, due to the formation of the dimeric species previously identified with NMR spectroscopy.

**Figure 4:**
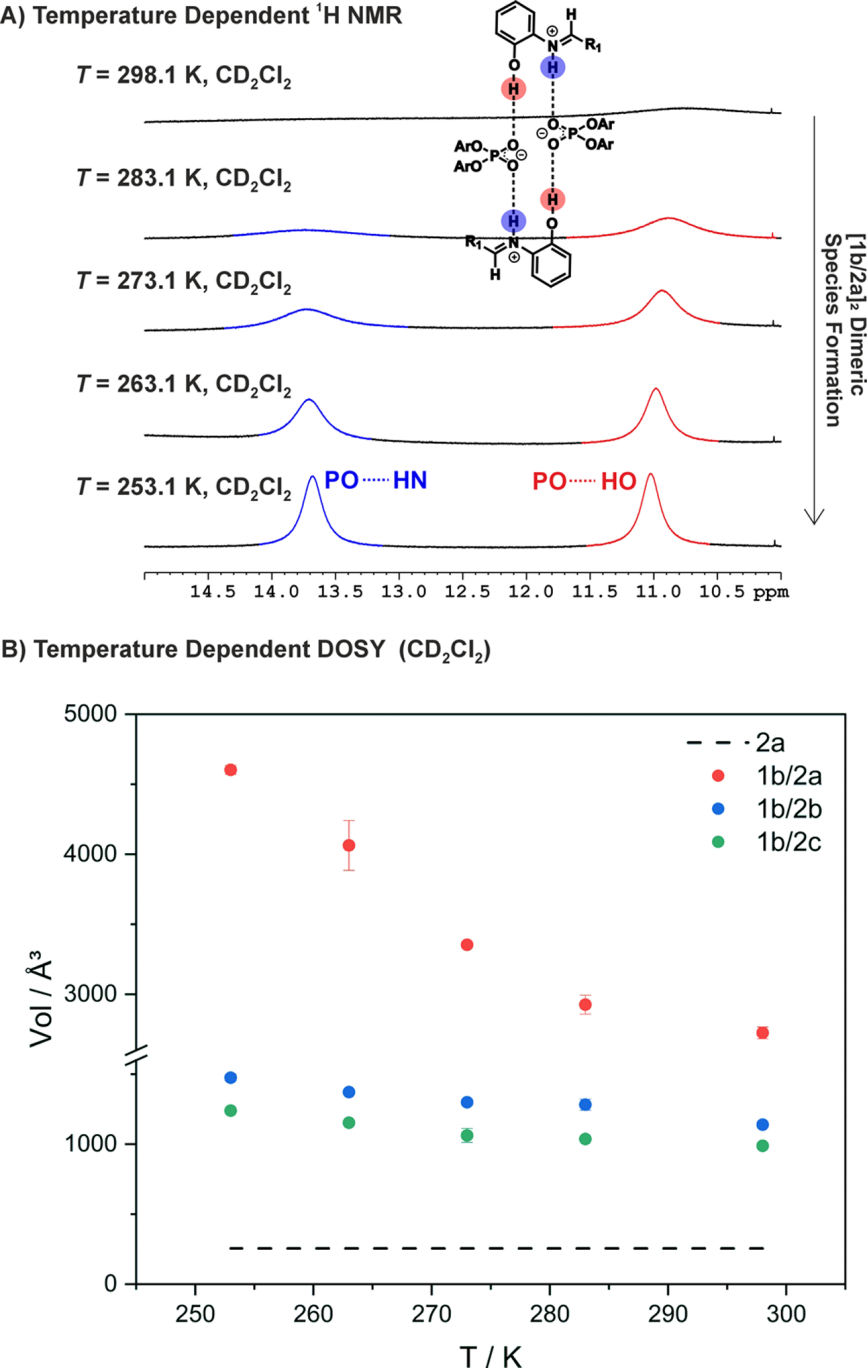
A) H-Bond section of the ^1^H NMR spectra of **1b/2a** recorded at five different temperatures, showing the POHN and POHO hydrogen bonds within the [CPA/imine]_2_ dimers. B) Volumes measured by DOSY spectroscopy are shown for the imine **2a** (black), binary complexes **1b/2b** (blue) and **1b/2c** (green), and for higher aggregates in the **1b/2a** system (red) at a 1:1 ratio and a concentration of 10 mM, respectively, in CD_2_Cl_2_ at 600 MHz. For the **1b/2a** system, the molecular volume increased from 2800 Å^3^ at room temperature to 4600 Å^3^ at −20 °C indicating increased dimer population at lower temperatures.

### Proving the Influence of Dimeric CPA/Imine Species

After gathering multiple indications for the influence of [CPA/imine]_2_ dimers—low-temperature NMR measurements, diffusion-ordered spectroscopy, substrate dependency, and the effects of temperature and catalyst loading—we further investigated their role in the Mannich-type reaction by examining the linear and nonlinear behavior in the **1b/2a** and **1b/2c** systems (Figure 5).

**Figure 5:**
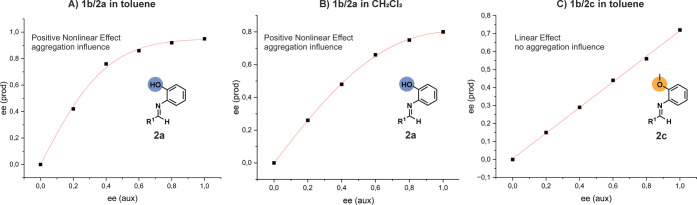
Probing catalyst systems **1b/2a** and **1b/2c** for a nonlinear relationship between product enantioselectivity and catalyst enantiopurity in the Mannich-type reaction (Table 1). A positive nonlinear effect in toluene (A) as well as in CH_2_Cl_2_ (B) provides clear evidence for the influence of higher aggregated species previously identified for the **1b/2a** system. C) For the **1b/2c** system, where no [CPA/imine]_2_ dimers could be identified, a completely linear behavior was observed, indicating no influence of aggregation.

These systems were chosen for their similar steric and electronic properties, yet they differ significantly in their ability to form [CPA/imine]_2_ dimers due to the presence of the *N*-2-hydroxyphenyl moiety. Probing catalyst systems for a nonlinear relationship between product enantioselectivity and catalyst enantiopurity has become a widely used mechanistic tool.^29–31,46^ A positive deviation from the linear relationship is referred to as an “asymmetric amplification”, which was first quantified by Puchot and coworkers through both theoretical studies and the first experimental description in asymmetric catalysis.^30^ A deviation from the linear behavior between product enantioselectivity and catalyst enantiopurity provides clear evidence of the influence of higher aggregates. Therefore, we conducted the Mannich-type reaction (Table 1) using six different catalyst enantiopurities *ee*(aux), ranging from 1 to 0, with the **1b/2a** system in toluene and CH_2_Cl_2_, as well as six different catalyst enantiopurities *ee*(aux), ranging from 1 to 0, with the **1b/2c** system in toluene (Figure 5). Additionally, for the **1b/2a** system, six further catalyst enantiopurities ranging from 0 to −1 in both solvents were tested and included in the SI (Tables S5 and S6). In fact, for the Mannich-type reaction in the **1b/2a** system using the imine with the *N*-2-hydroxyphenyl moiety, where we clearly identified [CPA/imine]_2_ dimers, we observed a deviation from linear behavior, resulting in an asymmetric amplification. This positive nonlinear effect (NLE) provides definitive proof of the influence of higher aggregated species. Additionally, to determine whether the nonlinear correlation is present in different solvents, we conducted experiments in CH_2_Cl_2_ as all our NMR measurements were performed in this solvent. Again, the reaction in CH_2_Cl_2_ also showed a deviation from the linear behavior, leading to an asymmetric amplification. Interestingly, when the solvent is changed from toluene to CH_2_Cl_2_, the yield drops to 71%, while the enantioselectivity also decreases to *ee*% = 81%. This is consistent with previous studies,^5,10^ where the Mannich-type reaction of imines with the *N*-2-hydroxyphenyl moiety in CH_2_Cl_2_ resulted in lower stereoselectivity and reactivity than that in toluene. This also explain why the asymmetric amplification effect was stronger in toluene, likely due to the higher polarity of CH_2_Cl_2_, which breaks hydrogen bonds of the aggregates more effectively and thereby reducing its influence.^47,48^ When examining the relationship between product enantioselectivity and catalyst enantiopurity for the **1b/2c** system, where no [CPA/imine]_2_ dimers could be identified, a completely linear behavior was observed, even in toluene, where the strong positive NLE for the **1b/2a** system was observed. This indicates no aggregation influence and therefore explains the overall reduced reactivity and enantioselectivity in the Mannich-type reaction (yield drops to 5%, while enantioselectivity also decreases to *ee*% = 72%). Furthermore, a linear relationship is also observed for the stronger Brønsted acid system **1c/2a**, where low-temperature NMR measurements revealed only a single H-bond in the ^1^H spectra, indicating only a monomeric complex between the NTPA catalyst and imine **2a** bearing the *N*-2-hydroxyphenyl moiety (Figure S46). Based on our NMR measurements of the [CPA/imine]_2_ dimer structure, which confirm the presence of an aggregate consisting of two chiral ligands, we fitted the nonlinear reaction behavior to the simplest ML_2_ model. This model was originally developed by Kagan and later refined by Blackmond and coworkers by consideration of the kinetic behavior.^29–31,46^ The model describes an asymmetric catalytic reaction based on two enantiomeric chiral ligands, where three different catalyst species may be formed, two homochiral and one heterochiral (*meso*) complexes. The two enantiopure catalysts give identical reaction rates, whereas the *meso* catalyst exhibits a reactivity of *g* relative to the enantiopure catalysts and the relative concentrations are fixed by an equilibrium constant *K*. Larger values of *K* indicate predominance of the *meso* species while values of *g* less than 1 indicate that the *meso* species is a less active catalyst. If the *meso* species is less active, then an asymmetric amplification in product enantioselectivity is observed. Applying the ML_2_ model fit to the **1b/2a** system in toluene yielded values of *K* = 10.2 and *g* = 0.02, whereas in CH_2_Cl_2_, the values obtained were *K* = 2.6 and *g* = 0.11 (Figures S43, S44). Both **1b/2a** systems, in toluene and CH_2_Cl_2_, exhibited asymmetric amplification with *g* values smaller than 1, indicating that the *meso* species is less active than the enantiopure catalyst. For the **1b/2a** system in toluene, a larger equilibrium constant *K* is obtained, indicating a greater predominance of the *meso* species. This also explains why the positive nonlinear effect is stronger in toluene than in CH_2_Cl_2_ and the higher enantioselectivities obtained in toluene. In addition, we performed kinetic studies to determine whether the [CPA/imine]_2_ dimers actively participate in the reaction pathway. Therefore, we measured the initial reaction rates at five different catalyst concentrations (Figure S47). Indeed, a catalyst order of 1.26 was obtained at lower catalyst concentrations, which converged to 0 at higher catalyst concentrations (for further details, see SI, Chapter “Reaction Kinetics of the Mukaiyama-Mannich Reaction”). This trend is typical for dimeric reactions in combination with the occurrence of side reactions.^31,46^

Most importantly, the obtained catalyst order larger than 1 for low catalyst concentrations supports the participation of the dimers in the product formation. In conclusion, the kinetic studies are fully consistent with the NLE analysis (Figure 5). For the first time, we finally clarified the long-standing question of why the *N*-2-hydroxyphenyl moiety is so crucial for the reaction and demonstrated experimentally why it is essential for achieving high enantioselectivity and reactivity in CPA-catalyzed Mannich-type reactions. This is attributed to the formation of aggregated species, as demonstrated through experimental studies on temperature, catalyst loading, kinetics, and nonlinear effects, and further confirmed by low-temperature NMR measurements. This understanding is pivotal for future developments, as it opens new opportunities in catalysis by enabling the controlled design and application of catalyst aggregates in asymmetric catalysis.

### Molecular Dynamic**s** Simulations of [**1b/2a**]_2_ Dimers

As a detailed structural analysis by NMR spectroscopy for the **1b/2a** system was not fruitful due to signal overlap of several dimeric species showing at least 4 broad POHO hydrogen bonds, we finally aimed to gain deeper structural insights into the different [CPA/imine]_2_ dimers in the **1b/2a** system via molecular dynamics simulations. For a good sampling of the conformational space at low temperature, we performed replica exchange MD simulations using 62 replicas in the range of 190–453 K. For structural analysis, the three replicas at 190 192.5, and 195 K were selected. We considered two ion pairs to form a dimer if the distance between the nitrogen atoms of the two imines is less than 14 Å. Hydrogen bonds within the dimer were required to have a hydrogen-acceptor distance below 2.4 Å and a donor-hydrogen-acceptor angle of more than 105°. Justifications for these selection criteria are given in the SI. Using these three criteria, several structural conformers were identified, as depicted in Figure 6A. Based on the occurrence of the respective structures, free energy differences were calculated and summarized in Figure 6A. Structure motifs I and II are consistent with the [CPA/imine]_2_ complexes previously identified in our working group for **1d/2a**, whereas structure motif III has not been observed yet.^21^ For the bridged dimers (motifs I and II), two CPA molecules are interconnected by four hydrogen bonds over two imine molecules nested in between the CPAs. Both POHN hydrogen bonds can point toward one CPA, while two POHO hydrogen bonds are directed to the other CPA (motif I) or each CPA can form one POHN and one POHO hydrogen bond (motif II). For the first time, an additional structure motif III was observed, featuring one bridging imine and one nonbridging imine, which appears to present an intermediate between motif I and II and is stabilized by aromatic interactions between the imines and the CPA backbone. Considering the huge reactivity differences between the **1b/2a** (84% yield, see Table 1) in comparison to the **1d/2a** system (no yield, see Table 1), the open motif III seems to be essential for the efficiency of the dimer. We note that a traditional transition state (TS) analysis based on saddle points on the potential energy surface is not applicable to systems with high flexibility and a vast accessible conformational space.^49,50^ Therefore, an analysis of the substrate accessible surface area (SuASA) of the electrophilic carbon was used to reveal that motif III provides the most accessible site for a nucleophilic attack, with an average SuASA of 2.4 Å^2^, followed by motif I (1.1 Å^2^) and motif II (0.1 Å^2^). When the relative occurrence of these structural motifs is used as a normalization factor and the specifically exposed face of the iminium ion, thereby dictating product enantioselectivity, is taken into account, motif III emerges not only as the most accessible structure but also as the one whose preferred exposure leads to formation of the *S* enantiomer rather than the *R* enantiomer (for further details, see SI, Chapter “Calculation of the Substrate Accessible Surface Area and Prediction of Product Isomerism”). Therefore, we propose that the reaction with the ketene silyl acetal **4** occurs more readily in open motif III. Consistent with this proposal, the major isomer formed in the Mannich-type reaction with CPA **1b** (Table 2) is *S*-**3a**, correlating with the trends observed in the SuASA investigations. For CPA catalyzed transformations of imines bearing the *N*-2-hydroxyphenyl moiety, originally a monomeric bidentate binding to the catalyst was proposed.^5^ However, our detailed investigation regarding complex structures using low-temperature NMR spectroscopy combined with MD simulations for the first time revealed the presence of more aggregated species in these transformations. Furthermore, comprehensive experimental studies examining the effects of temperature and catalyst loading, along with a nonlinear analysis, demonstrated that the identified [CPA/imine]_2_ dimers participate in the reaction pathway, influencing both selectivity and reactivity by enhancing internal acidity.

**Figure 6:**
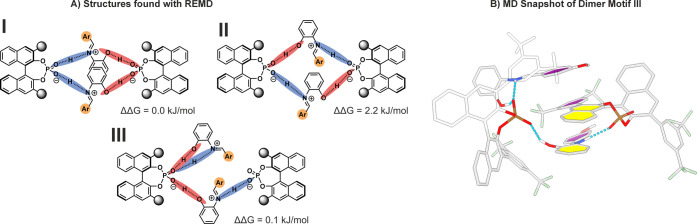
A) Two different dimer motifs were identified via REMD featuring two different CPA molecules bridged by two imines through hydrogen bonding (motifs I and II). Both POHN hydrogen bonds (blue) can point toward one CPA, while two POHO hydrogen bonds (red) are directed to the other CPA (motif I) or each CPA can form one POHN and one POHO hydrogen bond (motif II). An additional [CPA/imine]_2_ structure motif was found in which one imine is bridged by two different CPA molecules, while the second imine forms a bifurcated hydrogen bond with one CPA molecule (motif III). B) MD snapshot of dimer motif III which has not been observed in our previous studies, showing a [CPA/imine]_2_ structure with one bridging imine and one nonbridging imine.

## Conclusion

This study presents comprehensive experimental evidence elucidating the pivotal role of catalyst aggregation in enhancing both the reactivity and selectivity in chiral phosphoric acid catalysis. Through a combination of low-temperature NMR spectroscopy, MD simulations, nonlinear effect analysis, and detailed investigations of temperature and catalyst loading, we provided insight into the mechanistic role of the crucial *N*-2-hydroxyphenyl moiety in the imine substrate for the present Mannich-type reaction catalyzed by the CPAs. Its presence is essential as it promotes the formation of dimeric [CPA/imine]_2_ species, which we directly observe at low temperature by downfield-shifted hydrogen-bonded protons in the NMR spectra, reflecting an increase in internal acidity. These dimeric species enhance both reactivity and enantioselectivity given that the dimer has sufficient structural flexibility. In contrast, imines lacking this functionality or using stronger chiral Brønsted acids such as *N*-triflylphosphoramides formed only monomeric complexes and exhibited significantly reduced reaction yields and enantioselectivities. Unlike previous reports highlighting dimerization via acid–acid interactions and most often competing pathways of monomeric and dimeric species, with hydroxyimines, the dimerization is substrate-driven and the monomer pathway is rather unreactive. This allows for very high stereoselectivity at an inverse temperature/reactivity correlation (no reaction at room temperature/high yields-stereoselectivity at −78 °C). While a detailed TS analysis was limited due to system flexibility, MD simulations further identified three distinct [CPA/imine]_2_ species, with motif III featuring a more flexible structure with one imine bridging the two CPA catalysts and a second, nonbridging imine, which is more accessible for the substrate and therefore proposed to be the reactive species. These insights not only address a key mechanistic puzzle but also highlight the broader importance of higher-order aggregates in chiral Brønsted acid catalysis. The findings offer a powerful strategy for advancing asymmetric organocatalysis by designing and applying these aggregates in a controlled fashion.

## Supplementary Material



## Data Availability

Primary NMR data as well as calculations and Bruker pulse sequence codes are available free of charge at: 10.5281/zenodo.17939659. The authors have cited additional references within the Supporting Information.^51–72^

## Notes

Experimental details are provided in the Supporting Information: synthesis and characterization of substrates; NMR system screening; DOSY experiments; discussion of species; calculations; and NMR pulse sequences (DOCX)
