# Supplementary material for: Role of 2‑Hydroxyimines in Chiral Phosphoric Acid-Catalyzed Mannich-Type Reactions: Enhancing Reactivity and Selectivity via Dimerization
Source: J Am Chem Soc. 2026 May 6;148(19):19598–609. doi: 10.1021/jacs.5c22497 (PMC13195647; doi:10.1021/jacs.5c22497)
Supplement: Supplementary file 1 [file ja5c22497_si_001.docx]

**The Role of 2-Hydroxyimines in Chiral Phosphoric Acid-Catalyzed Mannich-Type Reactions: Enhancing Reactivity and Selectivity via Dimerization**

Markus Hecht^[a]^, Hendrik Fischer^[b]^, Wagner Silva^[a]^, Verena Eichstetter^[a]^, Christian L. Scholtes^[a]^, Ana Sofia Ferreira^[c]^, Eurico J. Cabrita^[c]^, Dominik Horinek*^[b]^ and Ruth M. Gschwind*^[a]^

Contents

[General Experimental Details 5](#_Toc227571721)

[NMR-Spectroscopy 5](#_Toc227571722)

[Pulse Sequences and Acquisition Parameters 5](#_Toc227571723)

[Mass Spectrometry 6](#_Toc227571724)

[Chemistry 6](#_Toc227571725)

[NMR Sample Preparation 7](#_Toc227571726)

[Synthesis of Imine Substrates 7](#_Toc227571727)

[Asymmetric Mukaiyama-Mannich reaction 12](#_Toc227571728)

[Synthesis of Amines 12](#_Toc227571729)

[HPLC Conditions 17](#_Toc227571730)

[HPLC Chromatograms 18](#_Toc227571731)

[NMR Spectroscopic Investigations 40](#_Toc227571732)

[Acid/Imine Systems 40](#_Toc227571733)

[Diffusion Ordered Spectroscopy (DOSY) 62](#_Toc227571734)

[Non-linear Analysis 65](#_Toc227571735)

[Theory 65](#_Toc227571736)

[Mukaiyama-Mannich Reaction Data 67](#_Toc227571737)

[Data Fitting 69](#_Toc227571738)

[Reaction Kinetics of the Mukaiyama-Mannich reaction 71](#_Toc227571739)

[Procedure for the ex-situ kinetics 71](#_Toc227571740)

[Computational Details 75](#_Toc227571741)

[General Molecular Dynamics (MD) Simulations Details 75](#_Toc227571742)

[Replica Exchange Molecular Dynamics Simulations Details 75](#_Toc227571743)

[Results and Statistics 76](#_Toc227571744)

[Calculation of the Substrate Accessible Surface Area and Prediction of Product Isomerism 83](#_Toc227571745)

[Force Field and Force Field Generation Details 88](#_Toc227571746)

# General Experimental Details

### NMR-Spectroscopy

NMR experiments were performed on Bruker Avance III HD spectrometers operating at a ^1^H base frequencies of 600.03 MHz and 600.28 MHz equipped either with a 5 mm triple resonance broadband inverse probe TBI-F (^1^H/^19^F, BB, ^2^H) with z-gradient (56 G∙cm^-1^) or a 5 mm diffusion probe DiffBB (BB, ^1^H/^19^F, ^2^H) with z-gradient (1700 G∙cm^-1^). Additionally, DOSY NMR experiments were performed using a Bruker Ascend 500 MHz spectrometer equipped with a 5 mm Prodigy triple resonance cryogenic probe CRPN2-TR (^1^H/^19^F, ^13^C, ^15^N).

The temperature of the spectrometer was regulated by a BVT 3900 unit and liquid nitrogen. Furthermore, samples for the characterization of in this work synthesized imines were measured on a Bruker III 400 MHz spectrometer equipped with 5 mm BBFO (BB/^19^F, ^1^H, ^2^H) probe head with z-Gradients. Spectrometer control and spectra processing was performed by Bruker Software TopSpin 3.2 PL7. For data processing, preparation and presentation Microsoft Excel, Origin 2020, ChemDraw 23.1.2 and CorelDraw 2025 were used.

### Pulse Sequences and Acquisition Parameters

Standard pulse sequences from the Bruker pulse sequence catalogue (zg, zg30, etc.) have been used. The respective pulse programs are not listed within the experimental part. The following acquisition parameter have been used:

^1^H-NMR: Pulse program zg30, Relaxation delay = 1.00 s, Acquisition time = 2.73 s, SW = 20 - 24 ppm, TD = 66 K, ns = 1 – 256;

^13^C NMR: Pulse program: zgpg30, Relaxation delay = 2.00 s, Acquisition time = 0.80 s, TD = 66 K; SW = 270.0 ppm, TD = 64k, NS = 1k – 4k;

^15^N NMR: Pulse program: zg30; Relaxation delay = 3.00 s, Acquisition time = 1.06 s; SW = 507.5 ppm, TD = 66 K; NS = 1K – 4K;

^19^F-NMR: Pulse program: zg30; Relaxation delay = 2.00 s, Acquisition time = 2.90 s, SW = 20.0 ppm, TD = 66k, NS = 16 – 64;

^31^P-NMR: Pulse program: zgpg30; Relaxation delay = 1.00 s, Acquisition time = 0.68 s, SW = 50.0 ppm, TD = 16k, NS = 32 – 2k;

### Mass Spectrometry

HRMS were measured at the Central Analytical Laboratory of the University of Regensburg on an Agilent Q-TOF 6540 UHD and a Jeol AccuTOF GCX instrument.

### Chemistry

Deuterated solvents were purchased from Deutero or Sigma Aldrich. Where dry solvents were essential, CD_2_Cl_2_ was refluxed over CaH_2_ for at least 5 h under Argon atmosphere and freshly distilled prior use. The chiral phosphoric acids were purchased from BLDpharm. 1,3,5-Trimethoxybenzene and Methyl trimethylsilyl dimethylketene acetal **4** were purchased from Sigma Aldrich.

### NMR Sample Preparation

General procedure for the preparation of binary and aggregated complexes (GP I):

The chiral Brønsted acid catalyst was weighted into a 5 mm NMR tube and dried for 30 min at 130 °C under reduced pressure. After cooling the tube to room temperature, imine was weighed directly into the NMR tube. The tube was evacuated and flushed with Argon three times. Deuterated and dry CD_2_Cl_2_ was added under Argon atmosphere, TMS was added, and the tube was closed and sealed with a parafilm. The sample was stored in a -80 °C freezer. For all samples a 1:1 ratio or 1:10 ratio of catalyst/imine was used. Even after careful sample preparation, partial hydrolysis of the imine took place. Therefore, the catalyst/imine-ratios were slightly different from 1:1 or 1:10. The concentration of 10 mmol L^-1^ or 100 mmol L^-1^ was used for all samples.

### Synthesis of Imine Substrates

General procedure for the synthesis of imines (GP II):

Molecular sieves 4 Å (4 g) were weighed in to a 50 ml Schlenk flask equipped with a stopper. The setup was connected to a vacuum line and was evacuated and dried with a heat gun at 350 °C. Under Argon flow, Aniline (10.5 mmol, 1.1 equiv.) and the respective aldimine (10 mmol, 1.0 equiv.) were added to the Schlenk flask under argon atmosphere and were dissolved in 20 ml Et_2_O. The solution was stirred at room temperature for 18 hours. The orange solution was filtered off from the molecular sieves and was concentrated under reduced pressure. The remaining solid was recrystallized from methanol. The product was obtained as white/yellow solid.

**2a:(E)-2-((4-methoxybenzylidene)amino)phenol**

Imine was synthesized following GP II and obtained as light-yellow needles (82 %) predominantly as *E* isomer (>99 % via ^1^H-NMR). ^1^H data was in accordance with literature.(*1*)

**^1^H-NMR** (400.1 MHz, CD_2_Cl_2_) δH = 8.66 (s, 1H), 7.90 (d, 2H, *J* = 8.8 Hz), 7.30 (dd, 1H, *J* = 7.9 Hz, *J* = 1.4 Hz), 7.24 (brs, 1H), 7.16 (m, 1H), 7.01 (d, 2H, *J* = 8.8 Hz), 6.98 – 6.87 (m, 2H), 3.88 (s, 3H) ppm.


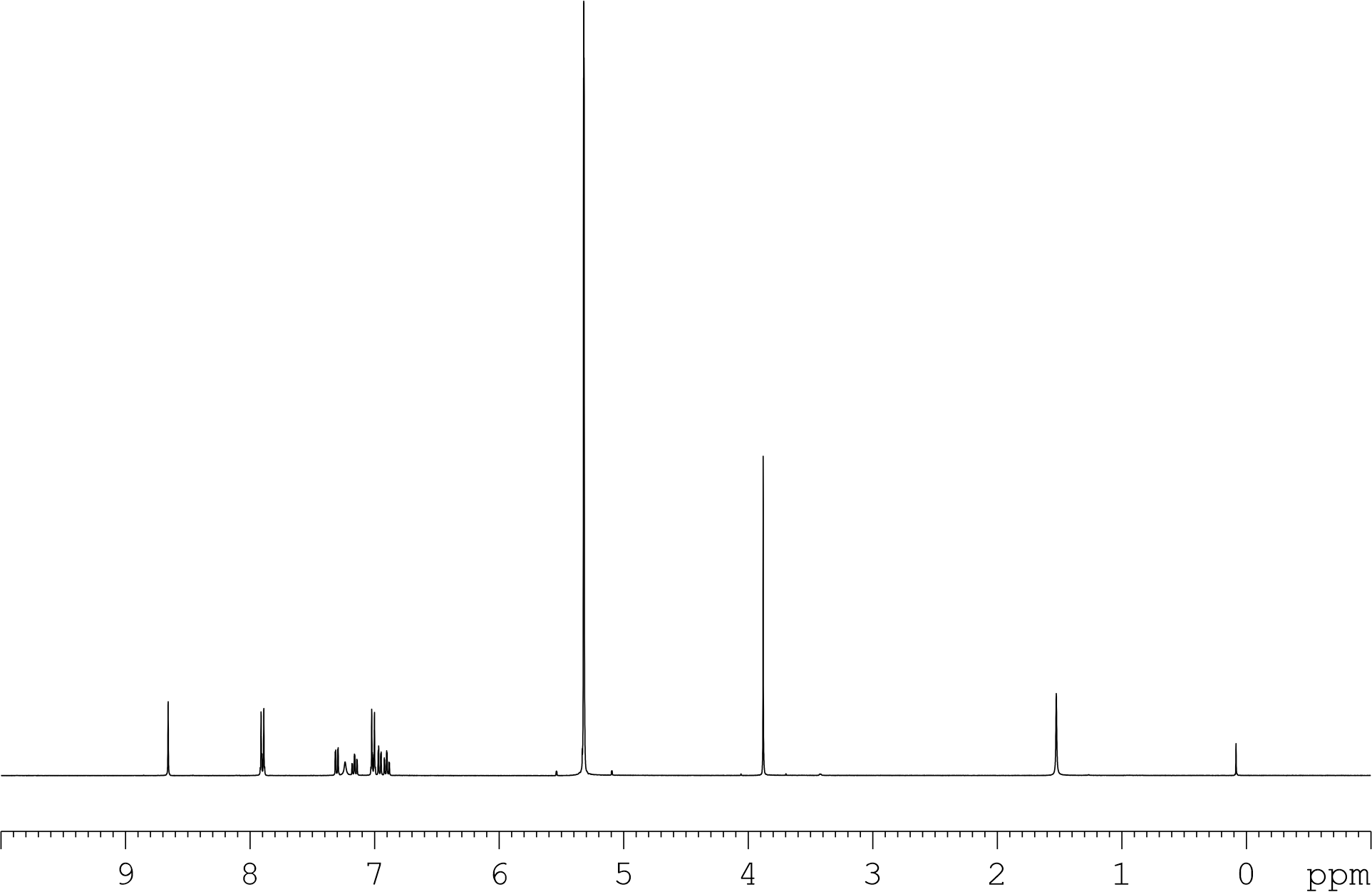


**2b:(E)-1-(4-methoxyphenyl)-N-phenylmethanimine**

Imine was synthesized following GP II and obtained as light-yellow needles (77 %) predominantly as *E* isomer (>99 % via ^1^H-NMR). ^1^H data was in accordance with literature.(*2*)

**^1^H-NMR** (400.1 MHz, CD_2_Cl_2_) δH = 8.39 (s, 1H), 7.85 (d, 2H, *J* = 8.8 Hz), 7.38 (t, 2H, *J* = 7.7 Hz) 7.23 – 7.15 (m, 3H), 6.99 (d, 2H, *J* = 8.8 Hz), 3.87 (s, 3H) ppm.


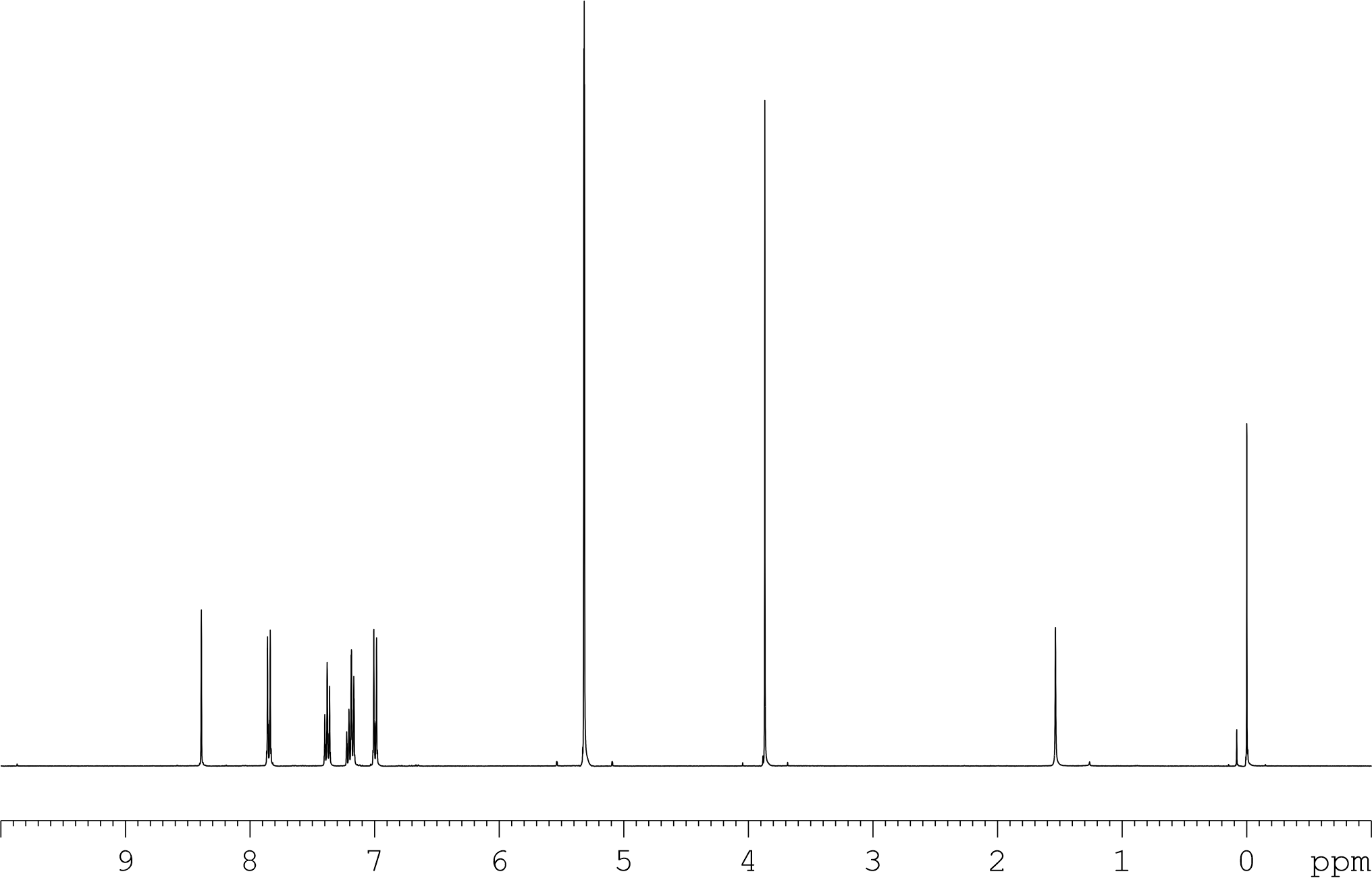


**2c:(E)-N-(2-methoxyphenyl)-1-(4-methoxyphenyl)methanimine**

Imine was synthesized following GP II and purified by bulb-to-bulb distillation to obtain a yellow oil (85 %) predominantly as *E* isomer (>99 % via ^1^H-NMR). ^1^H data was in accordance with literature.(*3*)

**^1^H-NMR** (400.1 MHz, CD_2_Cl_2_) δH = 8.38 (s, 1H), 7.87 (d, 2H, *J* = 8.8 Hz), 7.19 (m, 1H), 7.04 – 6.96 (m, 5H), 3.87 (s, 6H) ppm.


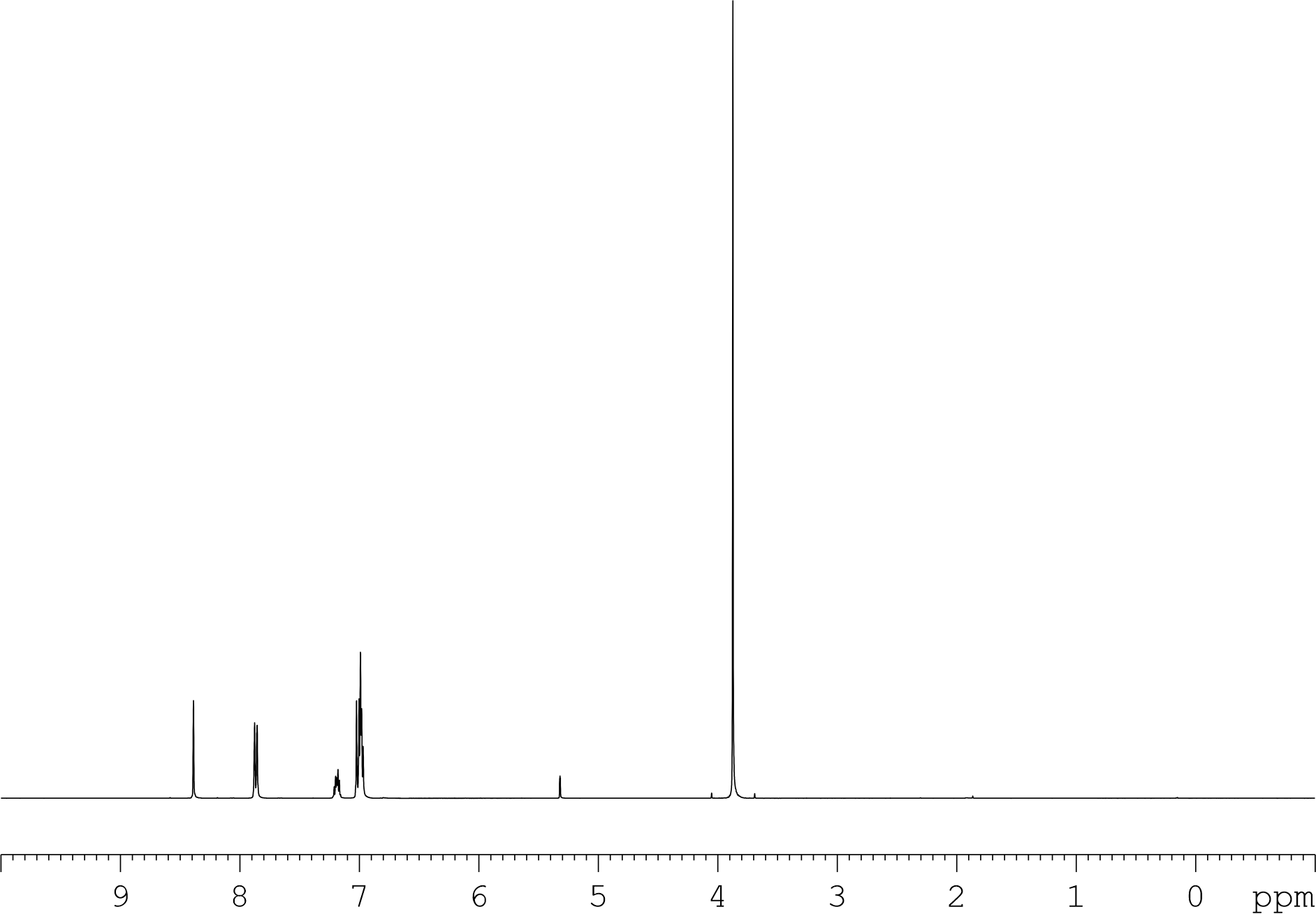


**2d:** **(E)-4-((4-methoxybenzylidene)amino)phenol**

Imine was synthesized following GP II and purified by bulb-to-bulb distillation to obtain light-yellow needles (50 %) predominantly as *E* isomer (>99 % via ^1^H-NMR). ^1^H data was in accordance with literature. (*4*)

**^1^H-NMR** (400.1 MHz, CD_2_Cl_2_) δH = 9.43 (s, 1H), 8.51 (s, 1H), 7.83 (d, 2H, *J* = 8.8 Hz), 7.14 (d, 2H, *J* = 8.8 Hz), 7.04 (d, 2H, *J* = 8.7 Hz), 6.78 (d, 2H, *J* = 8.7 Hz), 3.82 (s, 3H) ppm.


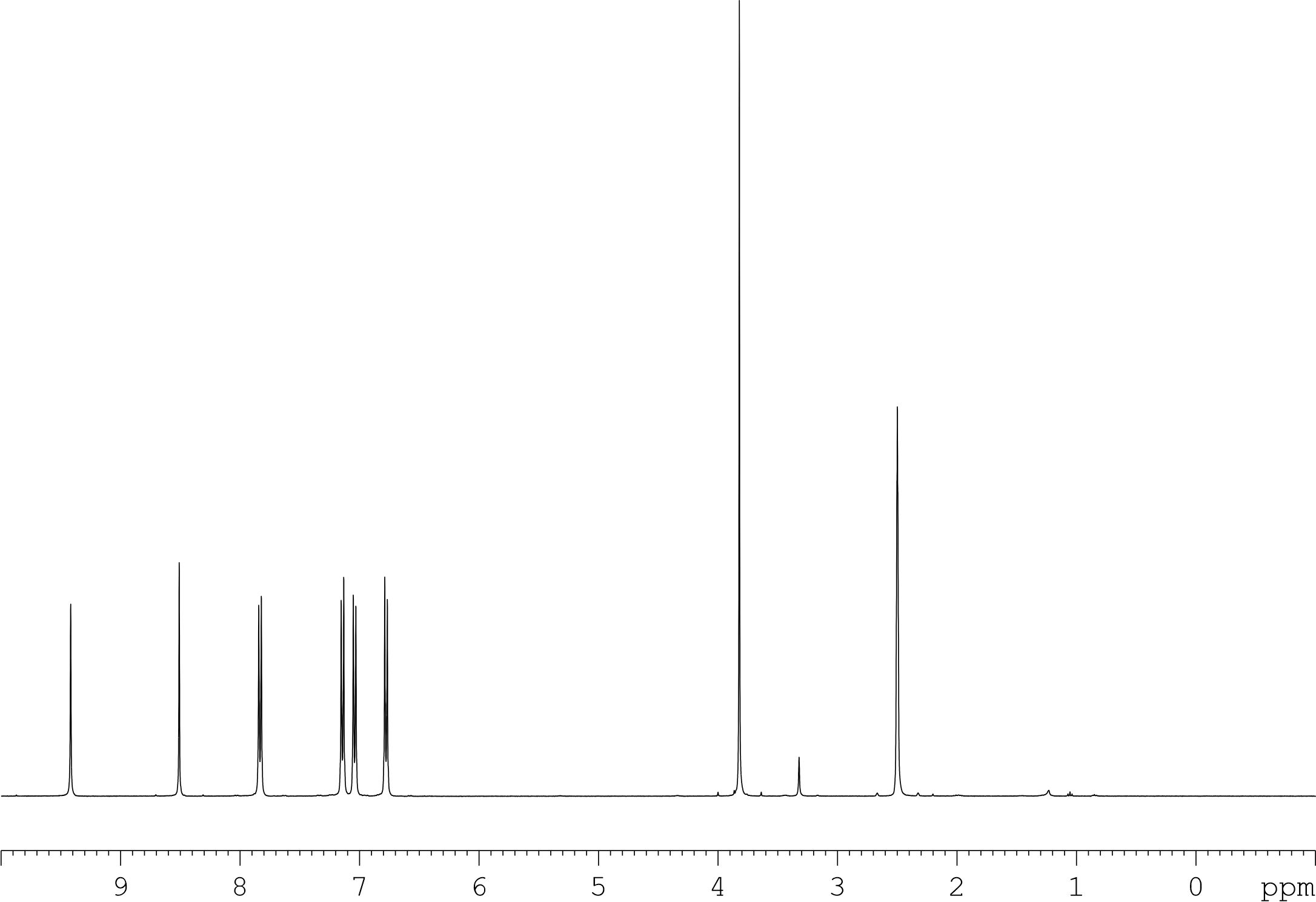


# Asymmetric Mukaiyama-Mannich reaction

### Synthesis of Amines

General procedure for the Asymmetric Mukaiyama-Mannich reaction (GP III):

To a solution of aldimine (0.2 mmol) and the chiral Brønsted acid catalyst (0.02 – 0.05 mmol) in dry toluene tempered to the temperature of interest (temperatures from -78 °C up to 80 °C) and then a solution of ketene silyl acetal (69.8 mg, 81.2 μL, 0.4 mmol) was added dropwise under argon atmosphere (r.t.). After being stirred at the temperature for 48 hours, the mixture was quenched by addition of saturated NaHCO_3_ (0.4 mL). THF (10 mL) and 1 M HCl (2 mL) was added at room temperature. The mixture was then stirred for one hour and extracted with CD_2_Cl_2_ (2x 10 mL). The combined organic layers were washed with brine, dried over anhydrous Na_2_SO_4_ and concentrated under reduced pressure. The crude product was purified by column chromatography (SiO_2_, hexane:ethylacetate = 3:1). The product was obtained as a white solid and enantiomeric excess was determined by varying Daciel Chiralpak column.

The specific conditions for each amine are provided in the chapter “HPLC Conditions”. After the pure amines **3a-c** were obtained and characterized by NMR spectroscopy, HPLC measurements were performed using the quenched crude reaction mixtures. Peak assignments were made by comparison with the retention times of pure, racemic samples, supported by literature data and confirmed using a DAD detector.

**3a:Methyl (S)-3-((2-hydroxyphenyl)amino)-3-(4-methoxyphenyl)-2,2-dimethylpropanoate**

Amine was synthesized following GP III and obtained as a yellow oil. ^1^H data was in accordance with literature.(*5*)

**^1^H-NMR** (400.1 MHz, acetone-*d_6_*) δH = 8.29 (s, 1H), 7.27 (d, 2H, *J* = 8.6 Hz), 6.84 (d, 2H, *J* = 8.7 Hz), 6.68 (dd, 1H, *J* = 8.3 Hz, *J* = 1.6 Hz), 6.53 – 6.46 (m, 1H), 6.40 – 6.34 (m, 2H), 5.33 (d. 1H, *J* = 8.8 Hz), 4.59 (d, 1H, *J* = 8.4 Hz), 3.74 (s, 3H), 3.63 (s, 3H), 1.25 (s, 3H), 1.18 (s, 3H) ppm.


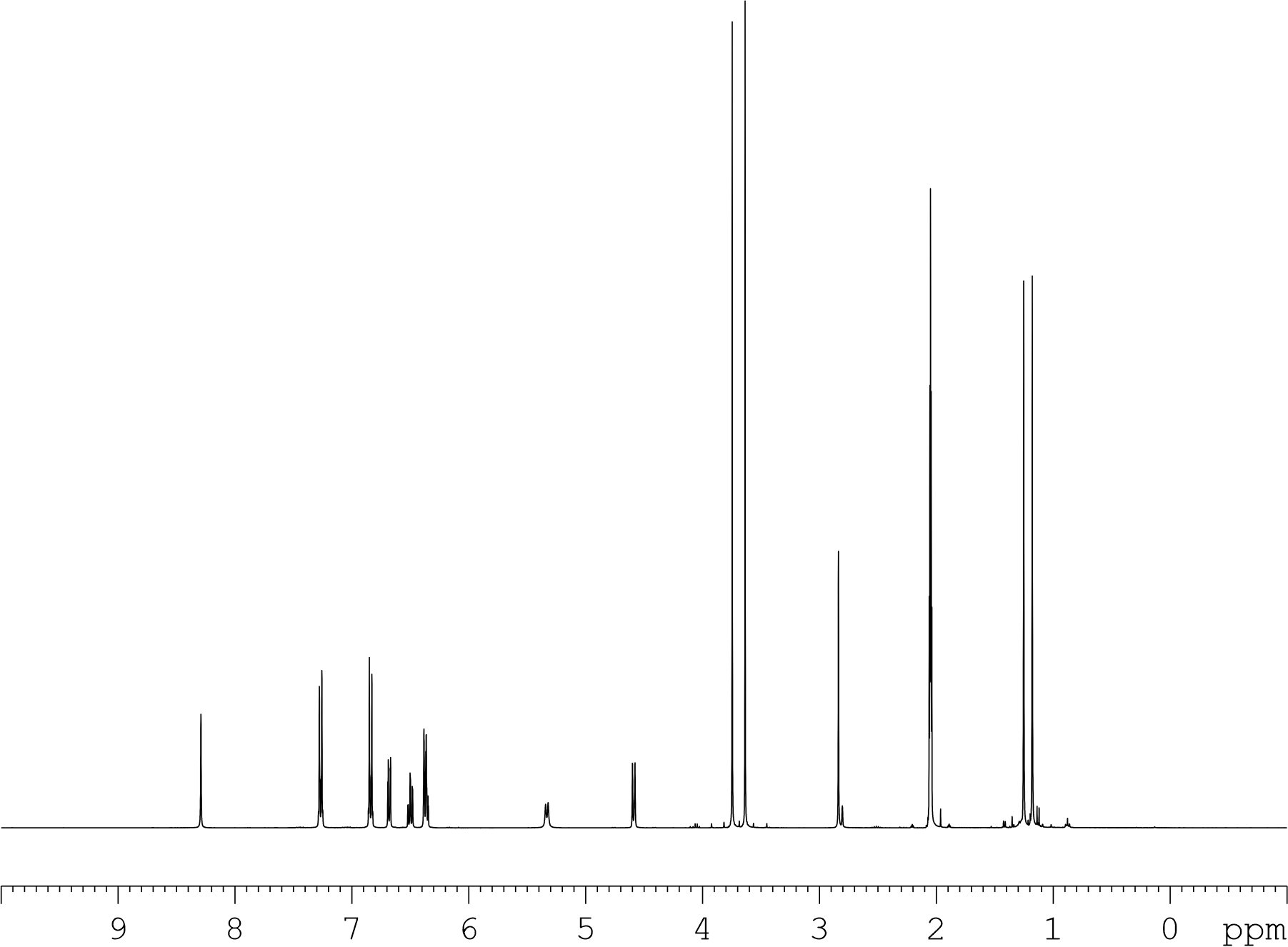


**3b:Methyl (S)-3-(4-methoxyphenyl)-2,2-dimethyl-3-(phenylamino)propanoate**

Amine was synthesized following GP III and obtained as a white solid. ^1^H data was in accordance with literature.(*5*)

**^1^H-NMR** (400.1 MHz, CDCl_3_) δH = 7.18 (d, 2H, *J* = 8.7 Hz), 7.05 (m, 2H), 6.81 (d, 2H, *J* = 8.6 Hz), 6.61 (t, 1H, *J* = 7.2 Hz), 6.49 (d, 2H, *J* = 7.7 Hz), 4.44 (brs, 1H), 3.77 (s, 3H), 3.65 (s, 3H), 1.26 (s, 3H), 1.15 (s, 3H) ppm.


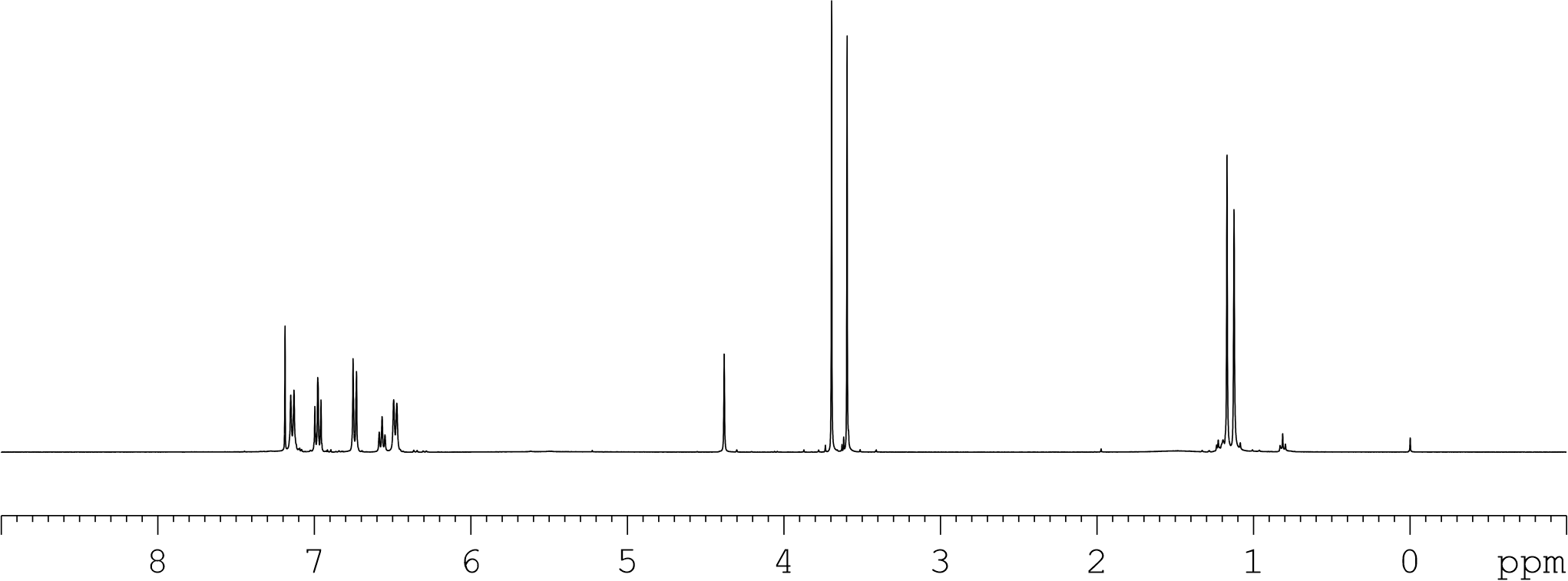


**3c:Methyl (S)-3-(4-methoxyphenyl)-3-((2-methoxyphenyl)amino)-2,2-dimethylpropanoate**

Amine was synthesized following GP III and obtained as a yellow oil. ^1^H, ^13^C and mass data were obtained.

**^1^H-NMR** (400.1 MHz, acetone-*d_6_*) δH = 7.24 (d, 2H, *J* = 8.6 Hz), ), 6.83 (d, 2H, *J* = 8.7 Hz), 6.76 (dd, 1H, *J* = 7.9 Hz, *J* = 1.3 Hz), 6.61 – 6.55 (m, 1H), 6.51 – 6.46 (m, 1H), 6.37 (1, 2H, *J* = 7.8 Hz), 5.47 (d. 1H, *J* = 8.2 Hz), 4.56 (d, 1H, *J* = 8.4 Hz), 3.88 (s, 3H), 3.74 (s, 3H), 3.64 (s, 3H), 1.26 (s, 3H), 1.17 (s, 3H) ppm.


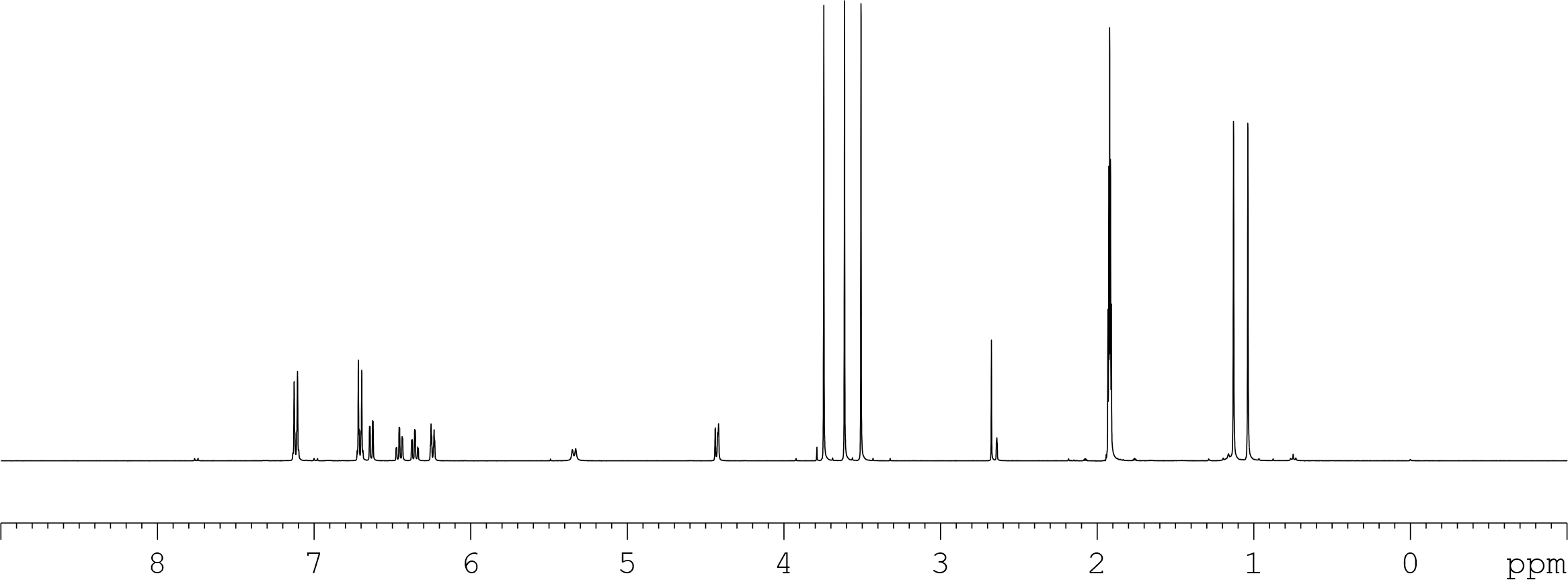


**^13^C-NMR** (100.6 MHz, acetone-*d_6_*) δC = 176.2, 159.0, 147.1, 137.2, 131.6, 129.3, 120.8, 116.2, 113.1, 110.9, 109.6, 63.0, 55.2, 54.5, 51.2, 47.0, 23.8, 20.5 ppm.


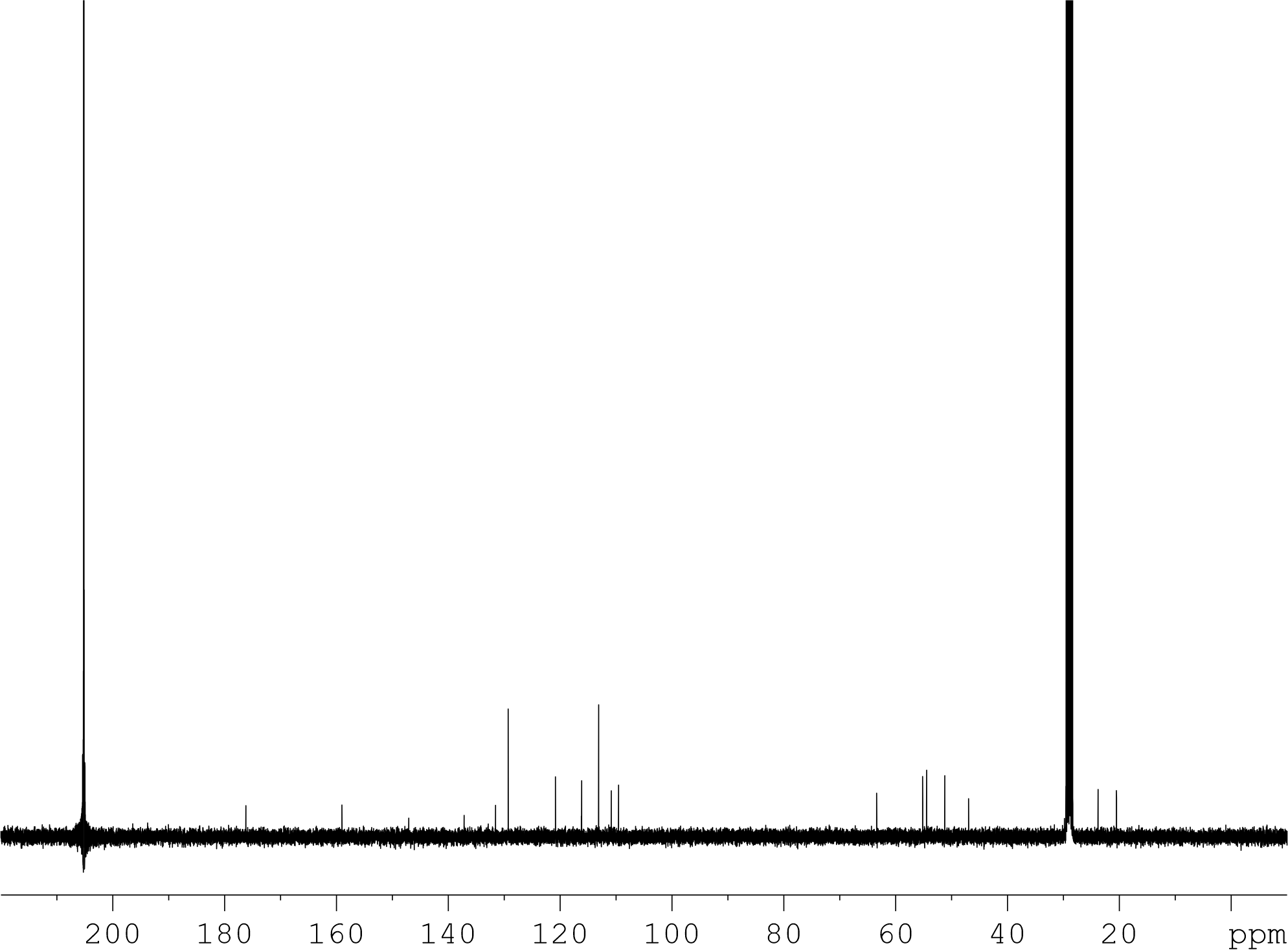


**HR-MS** (ESI, *m/z*): found 344.1898 (M-H)+ (calculated 343.1784 for M)

### HPLC Conditions

**3a:** The conditions for separating major and minor enantiomer were previously established in the group of Yamamoto.(*5*) HPLC, Daciel Chiralpak ADH, Hexane:i-PrOH = 5:1, Flow rate 0.7 mL/min. Major and minor isomer in all reactions were checked with a diode array detector, λ = 254 nm. The resulting UV-spectrum is shown below the respecting HPLC chromatogram.

**3b:** The conditions for separating major and minor enantiomer were previously established in our group.(*6*) HPLC, Daciel Chiralpak IA, Hexane:i-PrOH = 97:3, Flow rate 0.5 mL/min, Major and minor isomer in all reactions were checked with a diode array detector, λ = 254 nm. The resulting UV-spectrum is shown below the respecting HPLC chromatogram.

**3c:** The conditions for separating major and minor enantiomer were established in our group. HPLC, Daciel Chiralpak ADH, Hexane:i-PrOH = 98:2, Flow rate 0.5 mL/min, Major and minor isomer in all reactions were checked with a diode array detector, λ = 254 nm. The resulting UV-spectrum is shown below the respecting HPLC chromatogram.

### HPLC Chromatograms

Asymmetric Mukaiyama-Mannich reaction with aldimine **2a** and ketene silyl acetal catalyzed by CPA **1b** at – 78 °C and 10% catalyst loading in toluene.


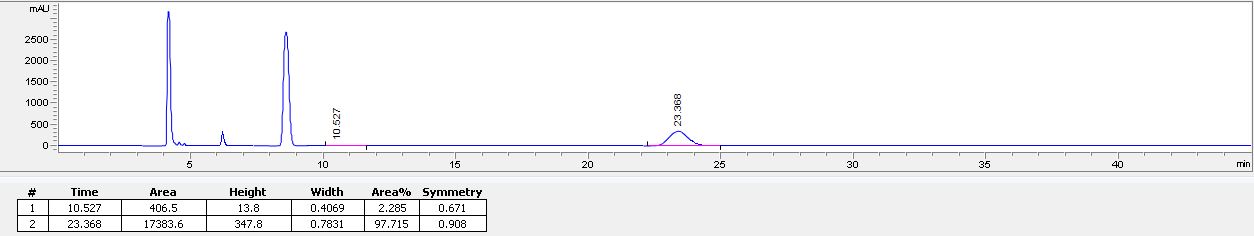


Racemic **3a**


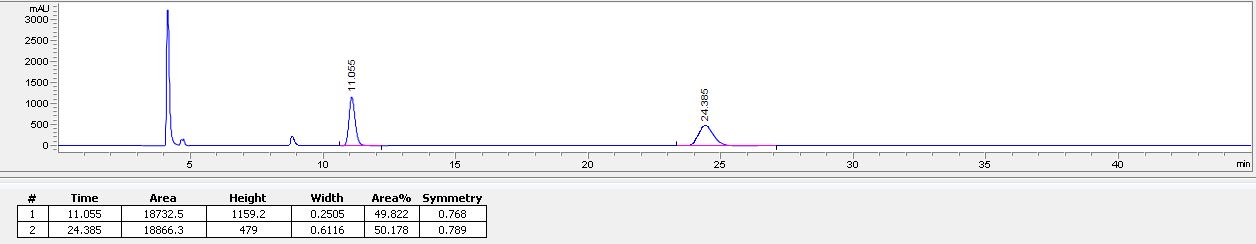


The resulting UV-spectrum of the amine **3a** at λ = 254 nm.


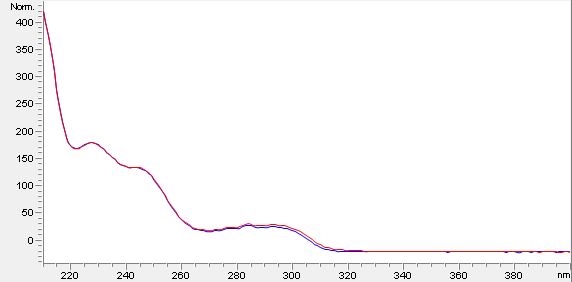


Asymmetric Mukaiyama-Mannich reaction with aldimine **2b** and ketene silyl acetal catalyzed by CPA **1b** at – 78 °C and 10% catalyst loading in toluene.


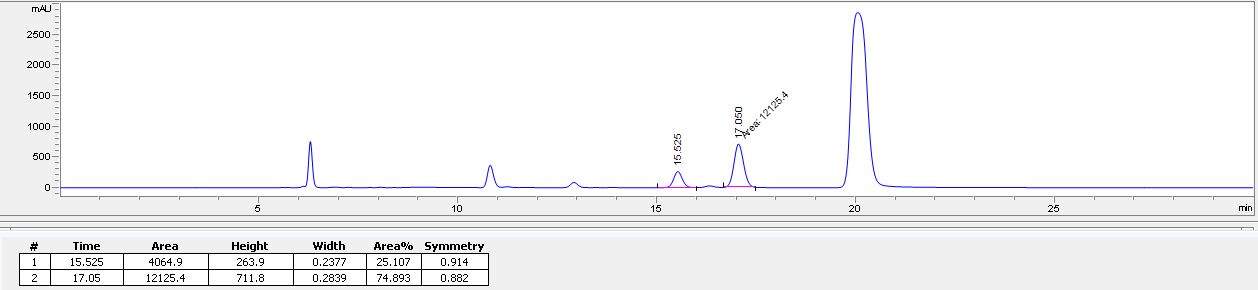


Racemic **3b**


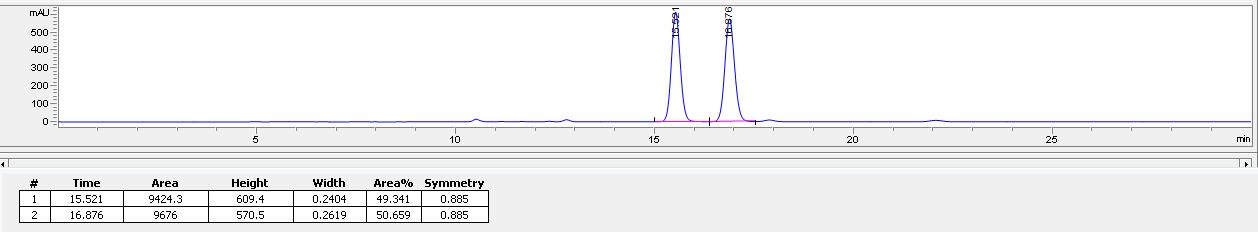


HPLC Chromatogram of 4-Methoxybenzaldehyde (decomposition product after workup)


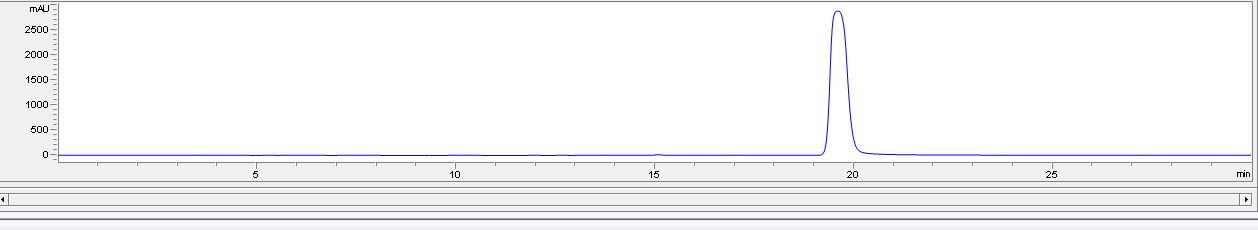


The resulting UV-spectrum of the amine **3b** at λ = 254 nm.


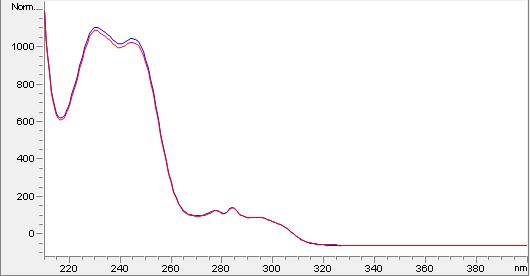


Asymmetric Mukaiyama-Mannich reaction with aldimine **2c** and ketene silyl acetal catalyzed by CPA **1b** at – 78 °C and 10% catalyst loading in toluene.


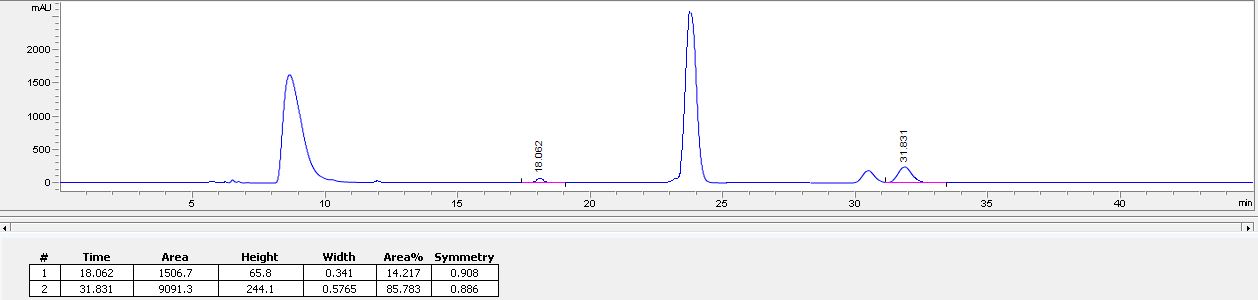


Racemic **3c**


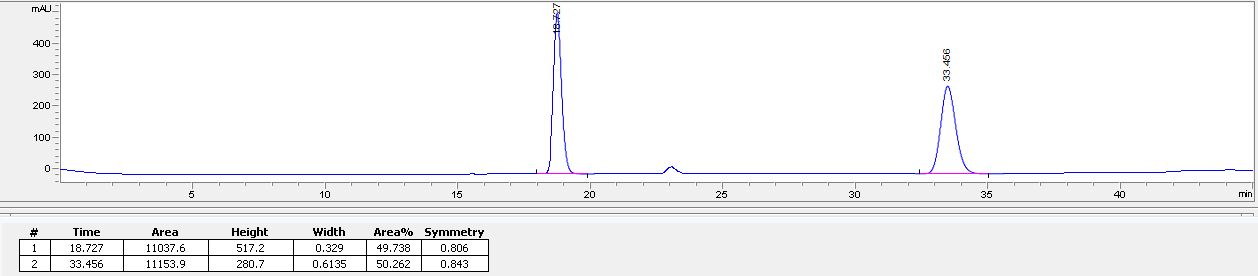


HPLC Chromatogram of 4-Methoxybenzaldehyde (decomposition product after workup)


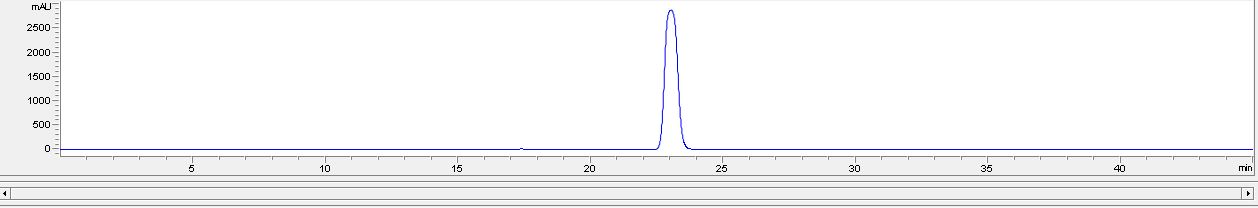


The resulting UV-spectrum of the amine **3c** at λ = 254 nm.


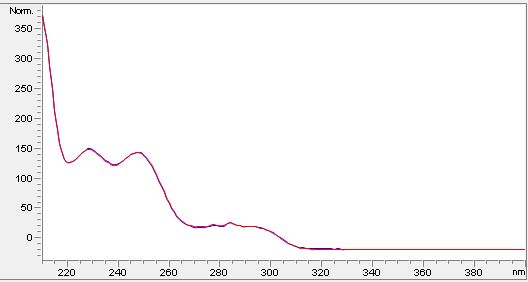


Asymmetric Mukaiyama-Mannich reaction with aldimine **2a** and ketene silyl acetal catalyzed by CPA **1b** at – 10°C and 10% catalyst loading in toluene.


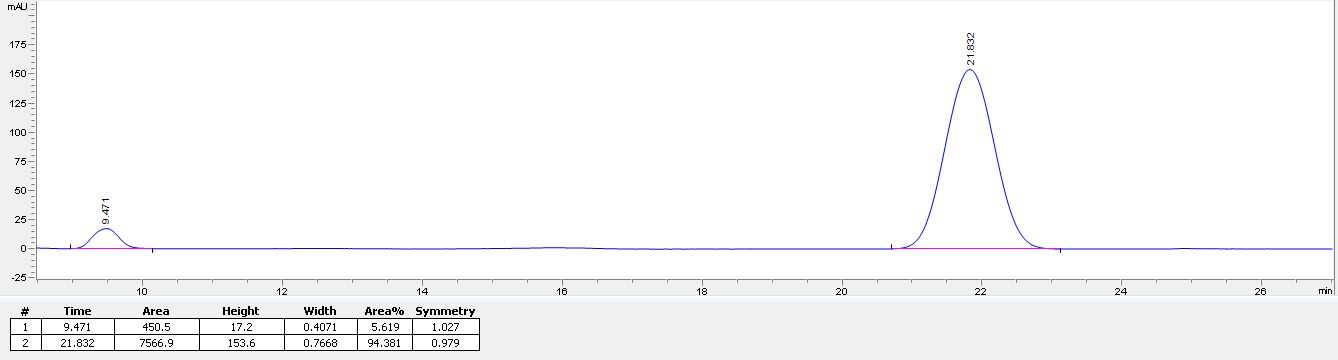


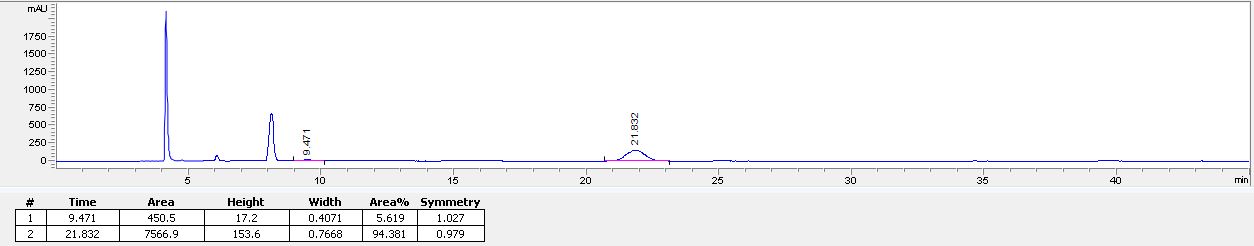


Asymmetric Mukaiyama-Mannich reaction with aldimine **2a** and ketene silyl acetal catalyzed by CPA **1b** at – 10°C and 25% catalyst loading in toluene.


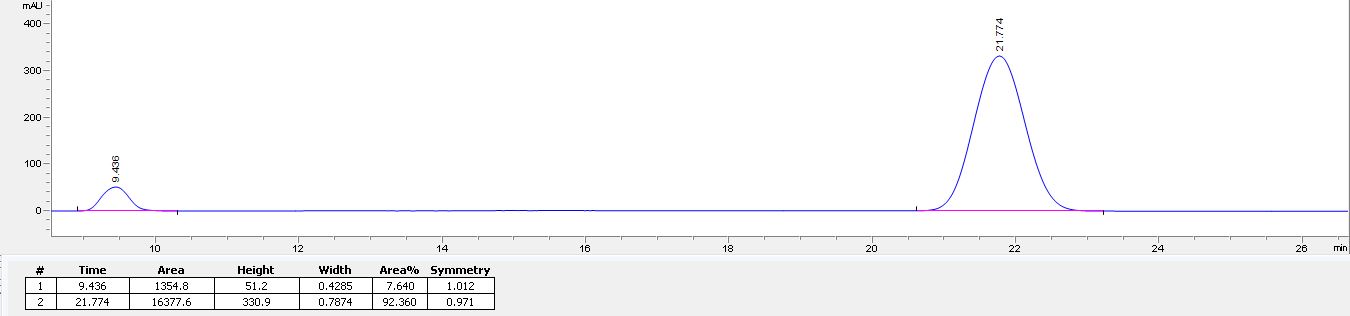


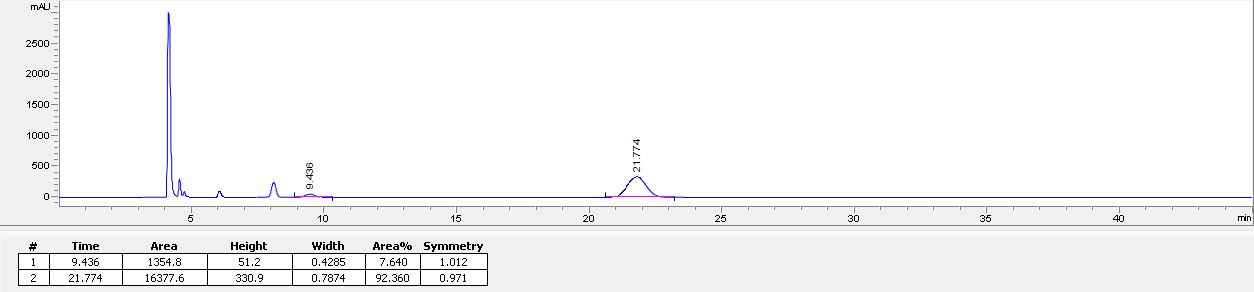


Asymmetric Mukaiyama-Mannich reaction with aldimine **2a** and ketene silyl acetal catalyzed by CPA **1b** at – 78°C and 25% catalyst loading in toluene.


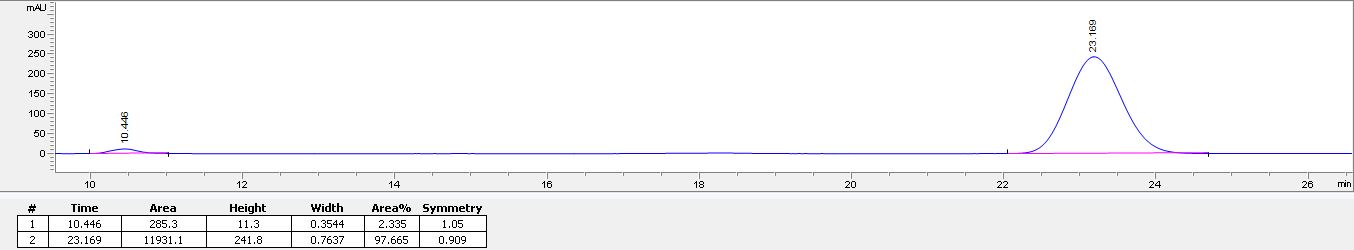


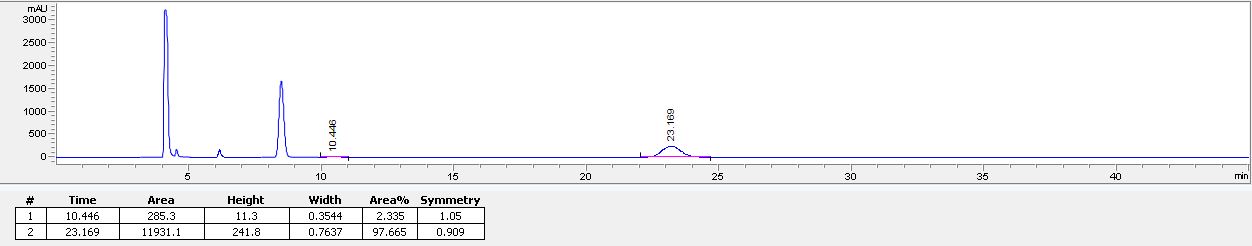


Asymmetric Mukaiyama-Mannich reaction with aldimine **2a** and ketene silyl acetal catalyzed by CPA **1b (90R+10S)** at – 78 °C and 10% catalyst loading in toluene.


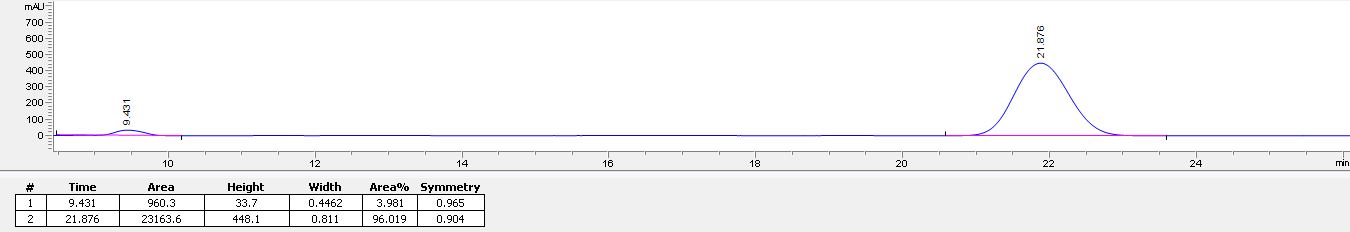


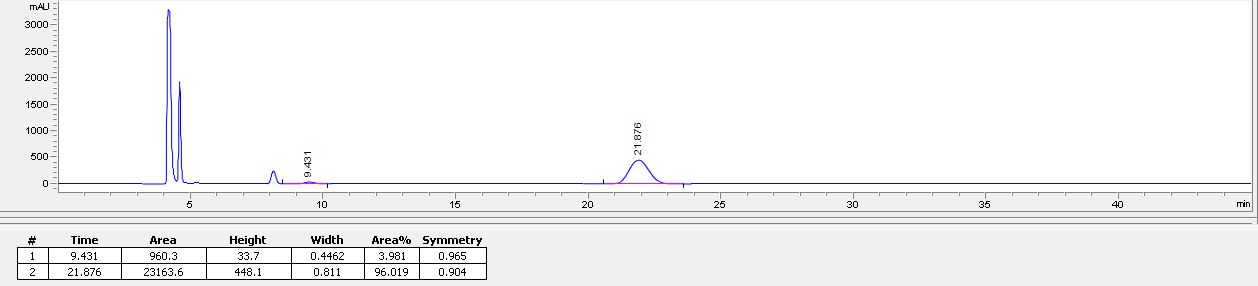


Asymmetric Mukaiyama-Mannich reaction with aldimine **2a** and ketene silyl acetal catalyzed by CPA **1b (80R+20S)** at – 78 °C and 10% catalyst loading in toluene.


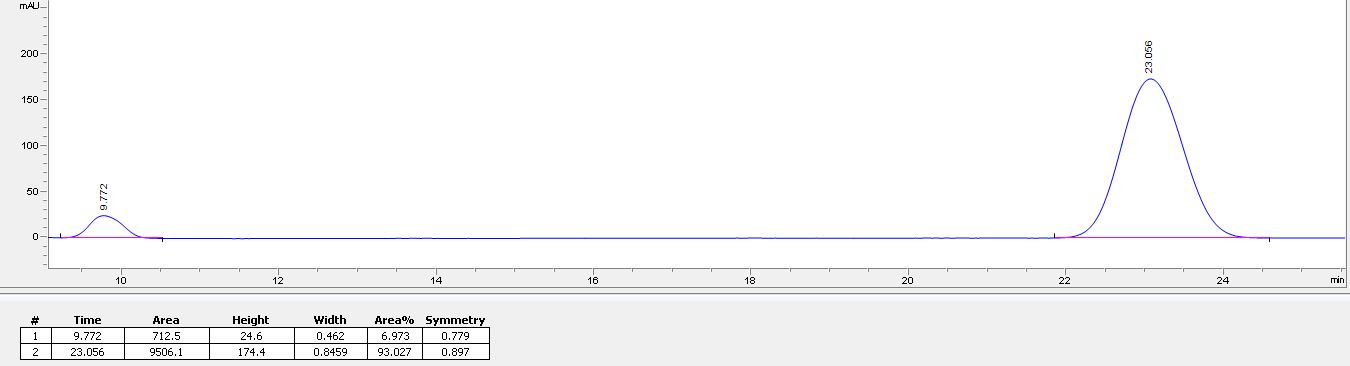


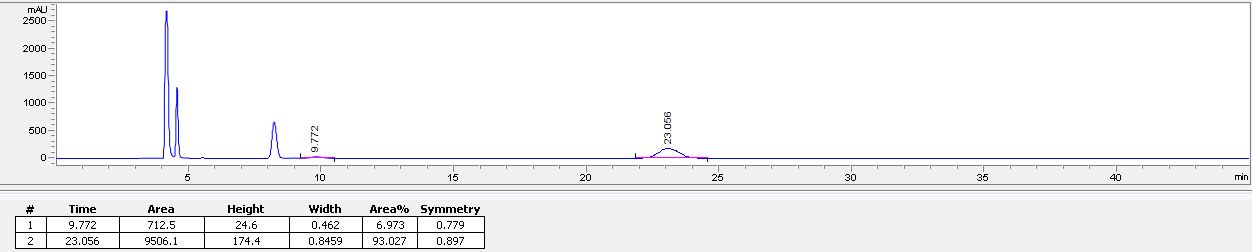


Asymmetric Mukaiyama-Mannich reaction with aldimine **2a** and ketene silyl acetal catalyzed by CPA **1b (70R+30S)** at – 78 °C and 10% catalyst loading in toluene.


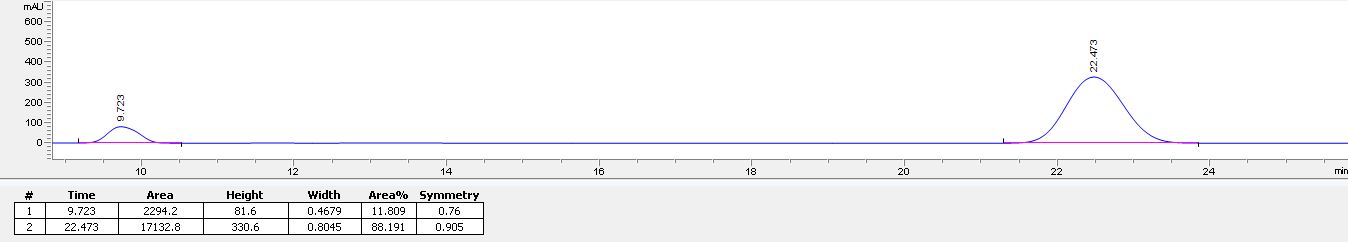


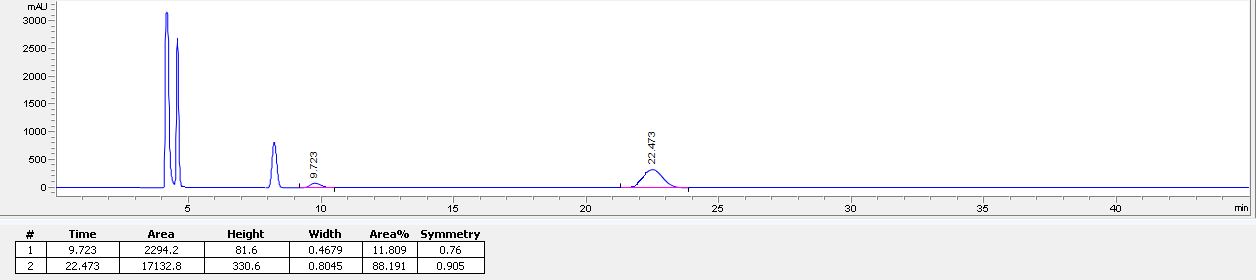


Asymmetric Mukaiyama-Mannich reaction with aldimine **2a** and ketene silyl acetal catalyzed by CPA **1b (60R+40S)** at – 78 °C and 10% catalyst loading in toluene.


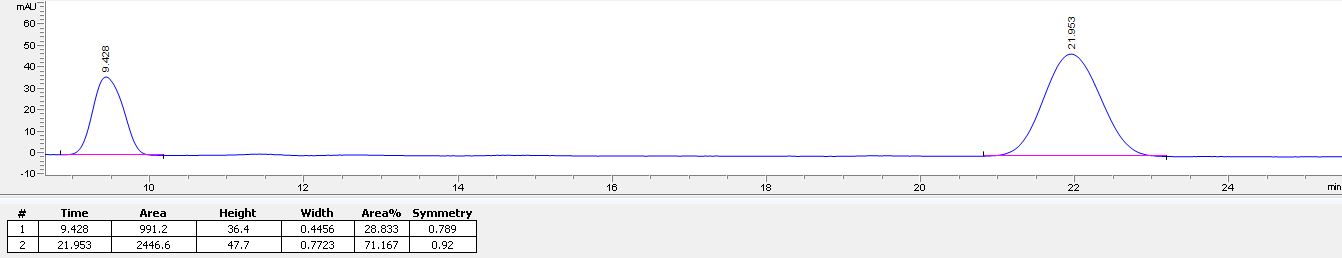


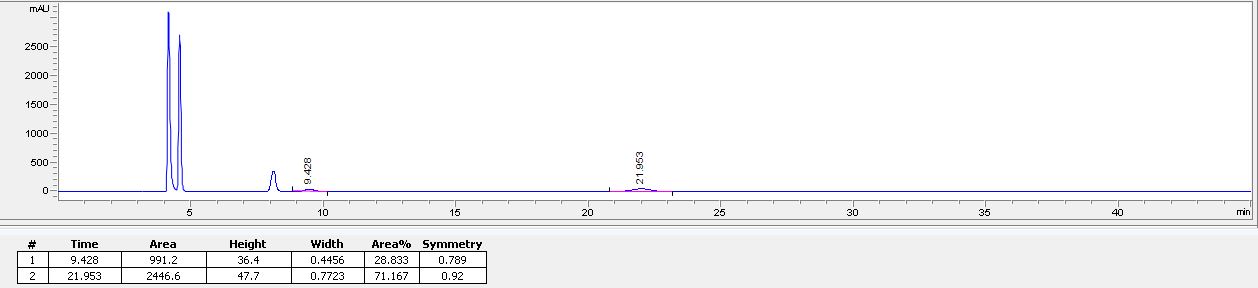


Asymmetric Mukaiyama-Mannich reaction with aldimine **2a** and ketene silyl acetal catalyzed by CPA **1b (50R+50S)** at – 78 °C and 10% catalyst loading in toluene.


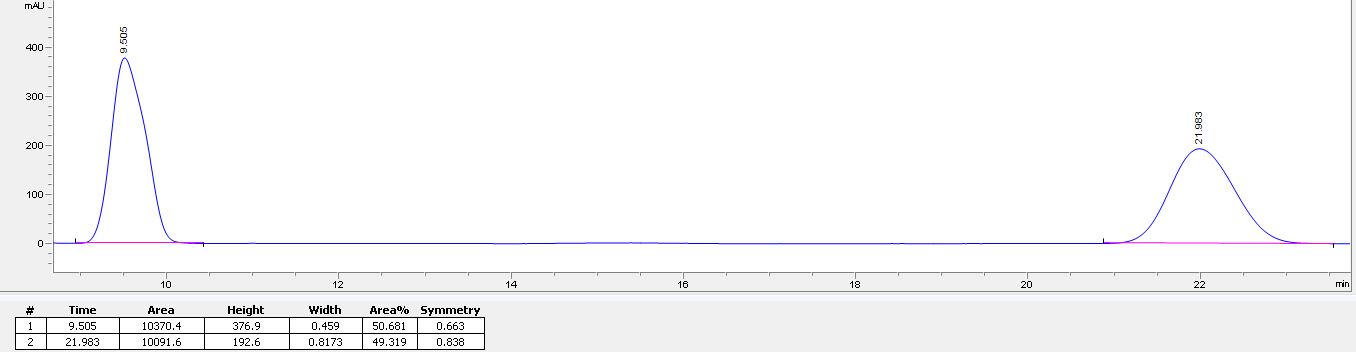


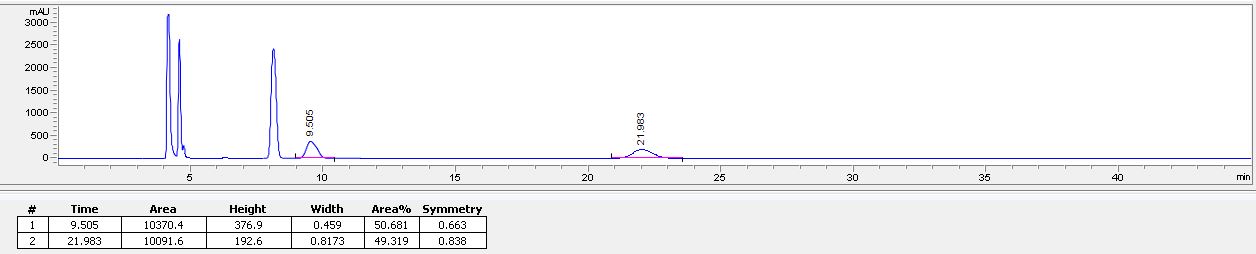


Asymmetric Mukaiyama-Mannich reaction with aldimine **2a** and ketene silyl acetal catalyzed by CPA **1b (40R+60S)** at – 78 °C and 10% catalyst loading in toluene.


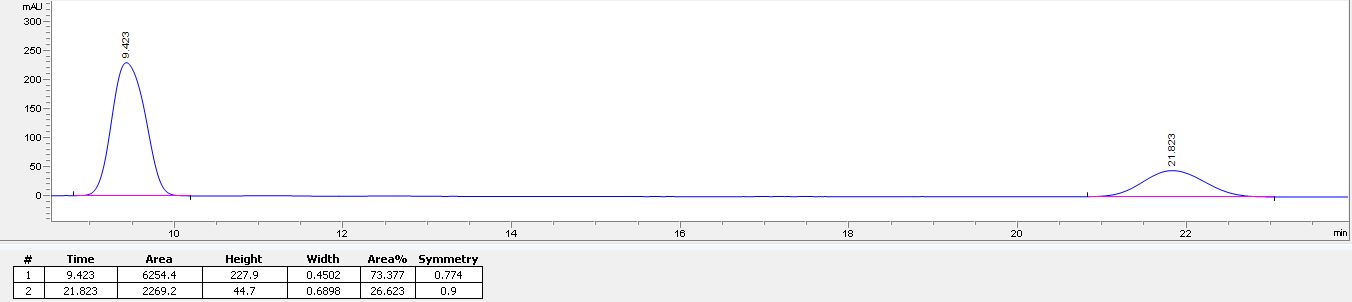


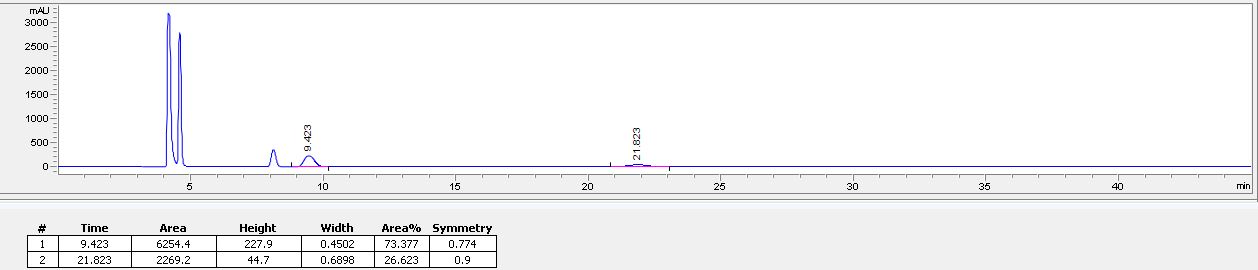


Asymmetric Mukaiyama-Mannich reaction with aldimine **2a** and ketene silyl acetal catalyzed by CPA **1b (30R+70S)** at – 78 °C and 10% catalyst loading in toluene.


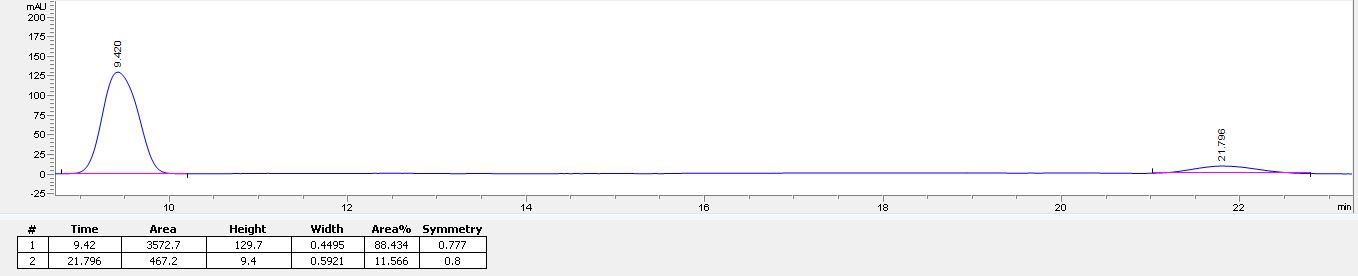


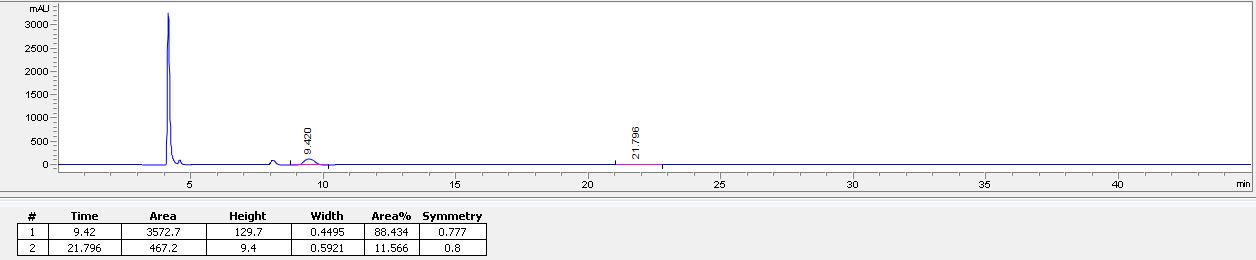


Asymmetric Mukaiyama-Mannich reaction with aldimine **2a** and ketene silyl acetal catalyzed by CPA **1b (20R+80S)** at – 78 °C and 10% catalyst loading in toluene.


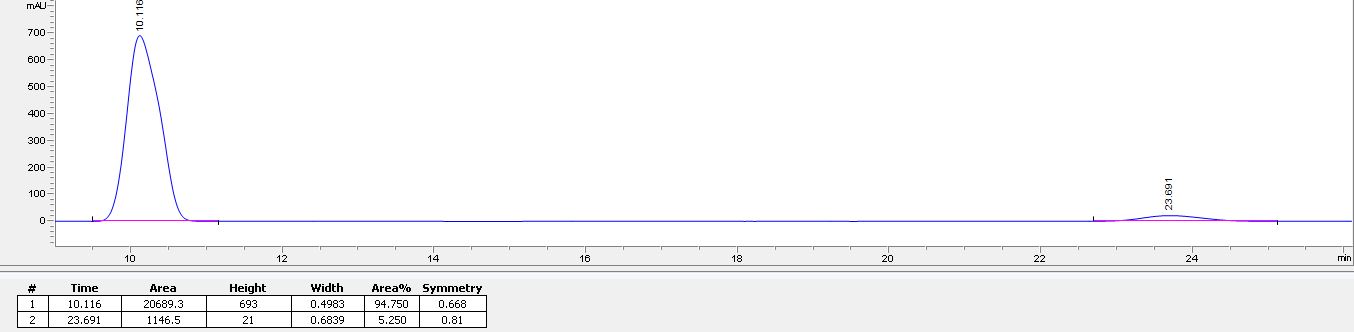


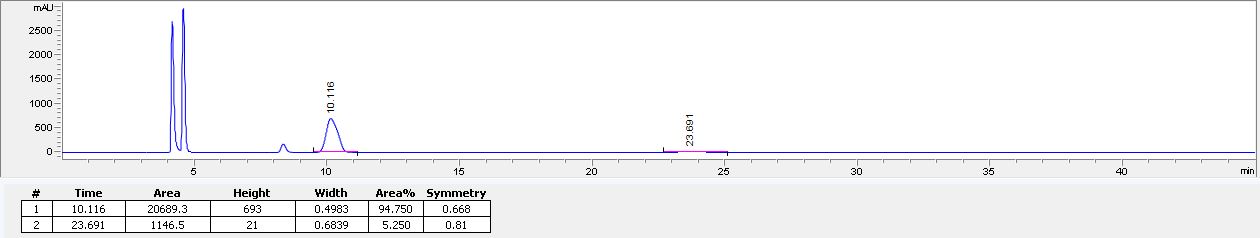


Asymmetric Mukaiyama-Mannich reaction with aldimine **2a** and ketene silyl acetal catalyzed by CPA **1b (10R+90S)** at – 78 °C and 10% catalyst loading in toluene.


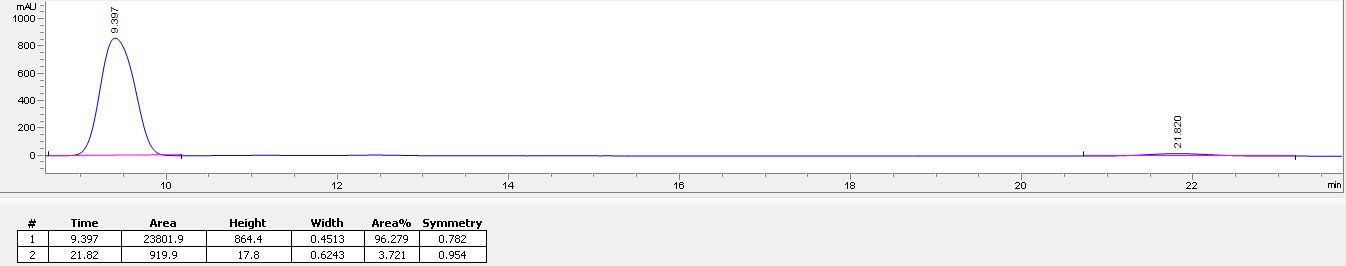


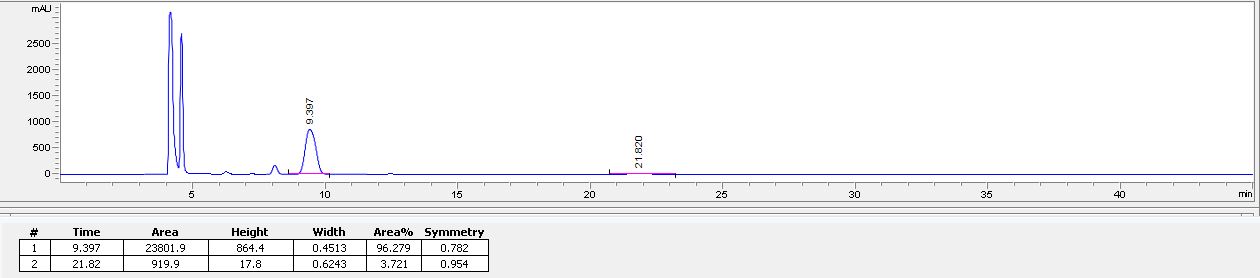


Asymmetric Mukaiyama-Mannich reaction with aldimine **2a** and ketene silyl acetal catalyzed by CPA **1b (100S)** at – 78 °C and 10% catalyst loading in toluene.


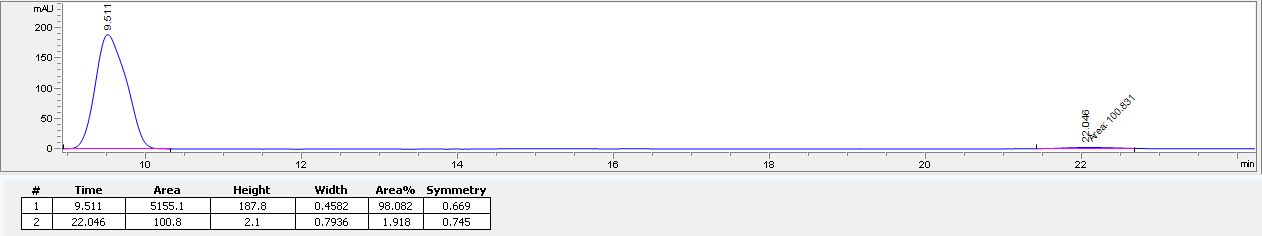


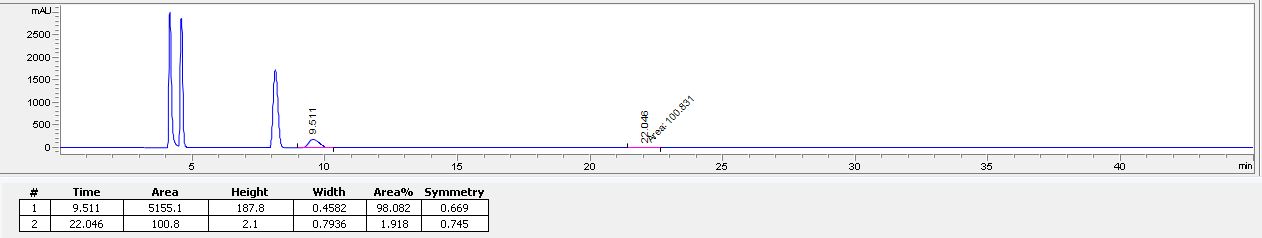


Asymmetric Mukaiyama-Mannich reaction with aldimine **2a** and ketene silyl acetal catalyzed by CPA **1b (100R)** at – 78 °C and 10% catalyst loading in CD_2_Cl_2_.


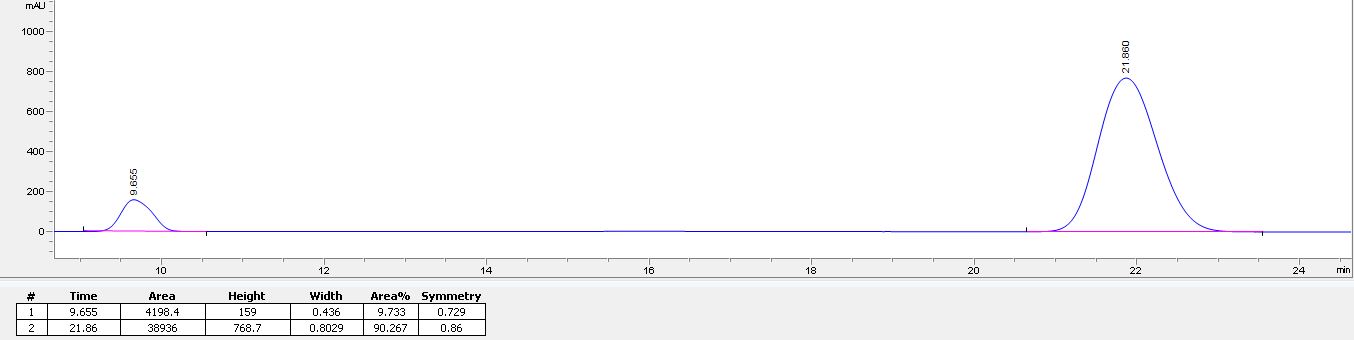


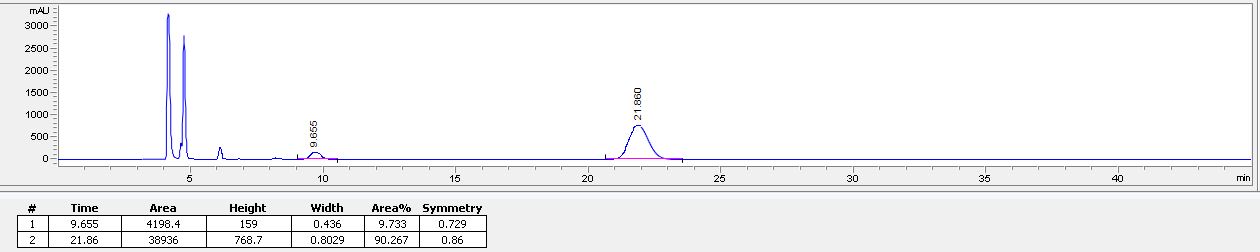


Asymmetric Mukaiyama-Mannich reaction with aldimine **2a** and ketene silyl acetal catalyzed by CPA **1b (90R+10S)** at – 78 °C and 10% catalyst loading in CD_2_Cl_2_.


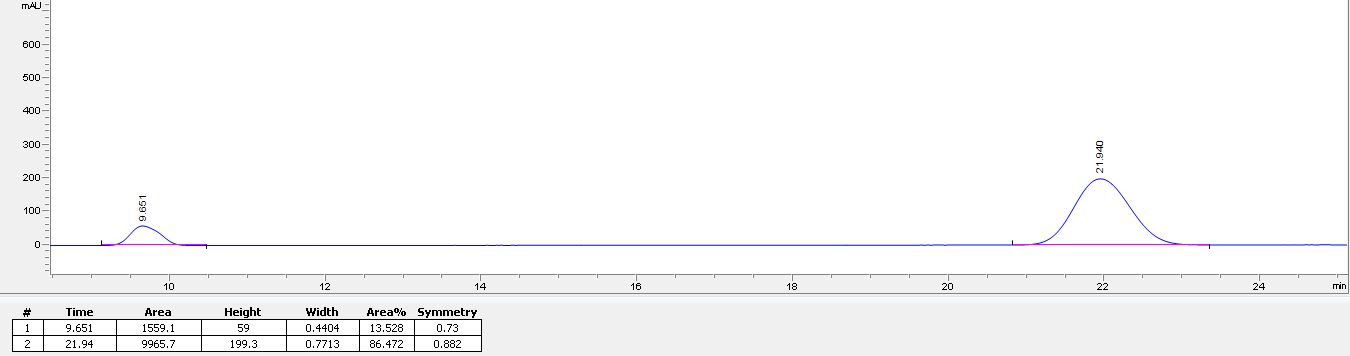


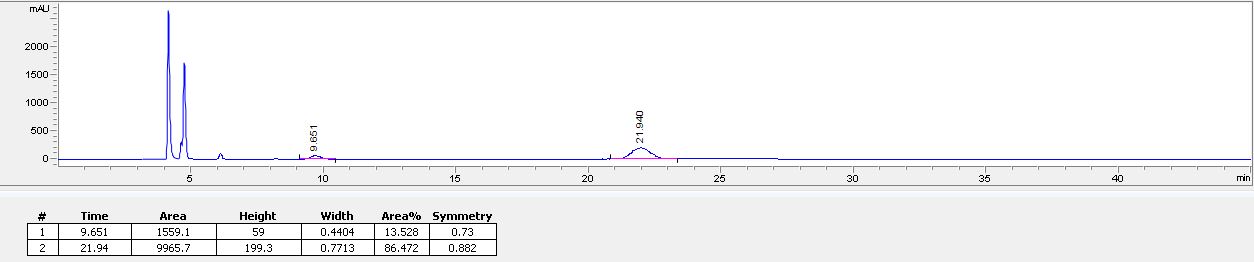


Asymmetric Mukaiyama-Mannich reaction with aldimine **2a** and ketene silyl acetal catalyzed by CPA **1b (80R+20S)** at – 78 °C and 10% catalyst loading in CD_2_Cl_2_.


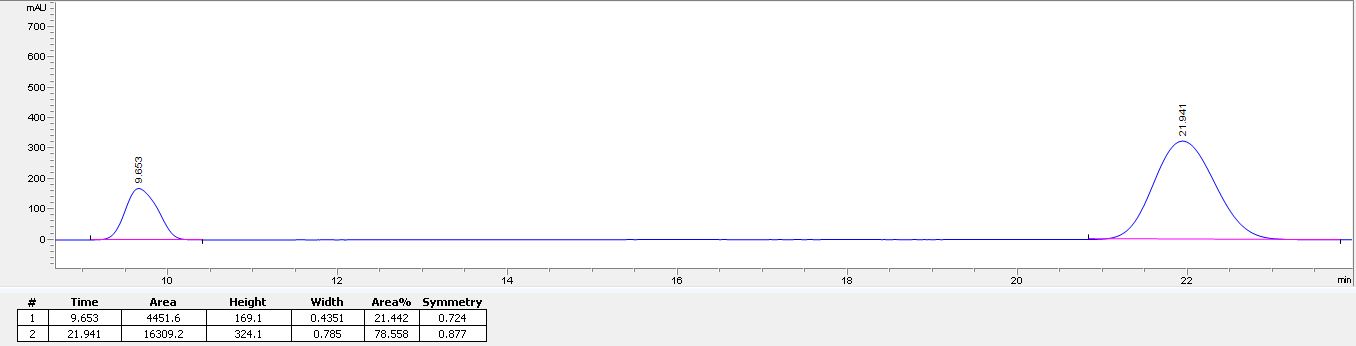


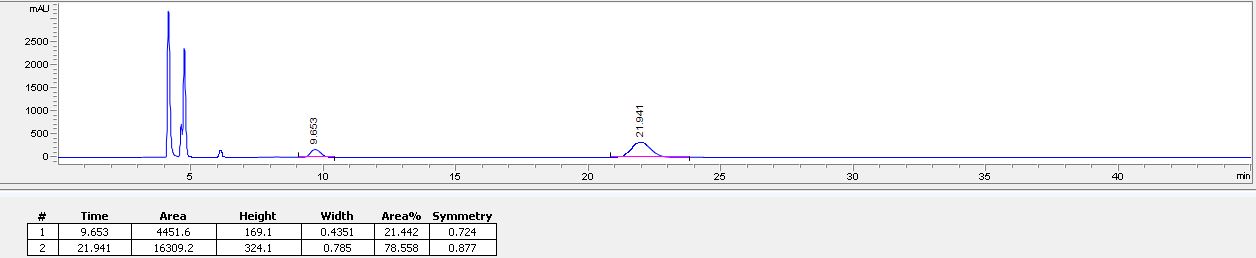


Asymmetric Mukaiyama-Mannich reaction with aldimine **2a** and ketene silyl acetal catalyzed by CPA **1b (70R+30S)** at – 78 °C and 10% catalyst loading in CD_2_Cl_2_.


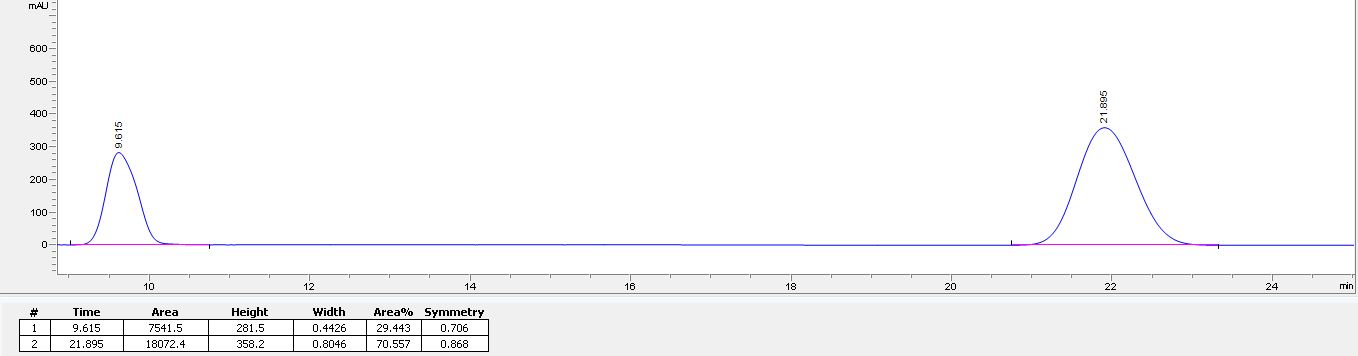


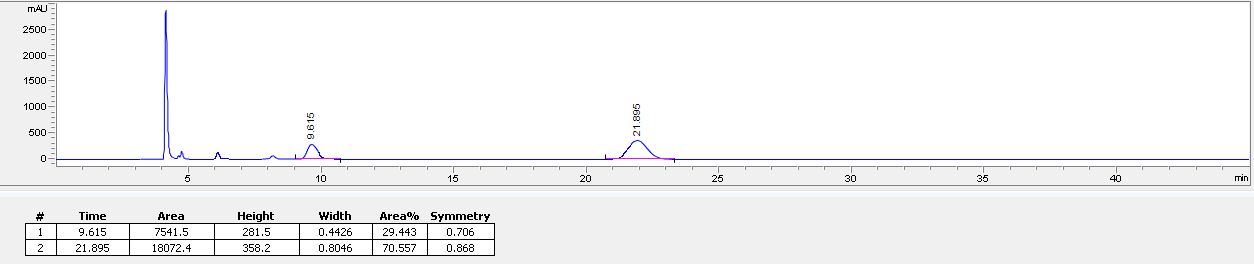


Asymmetric Mukaiyama-Mannich reaction with aldimine **2a** and ketene silyl acetal catalyzed by CPA **1b (60R+40S)** at – 78 °C and 10% catalyst loading in CD_2_Cl_2_.


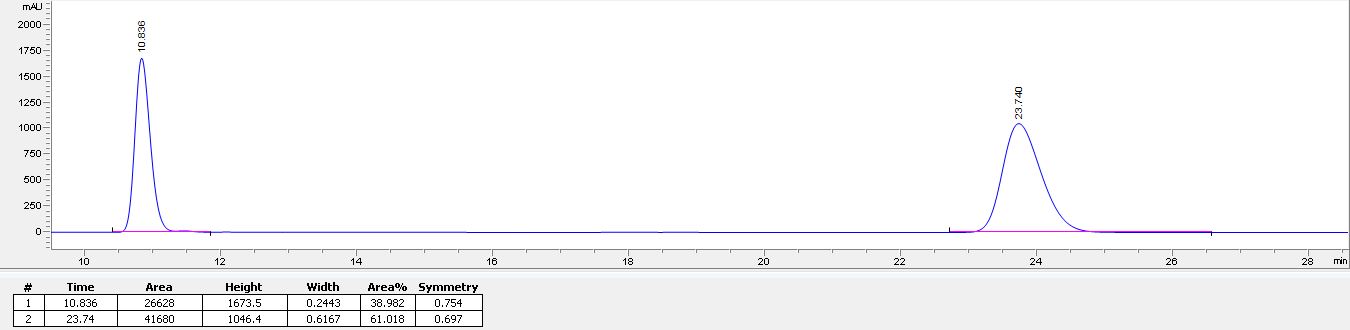


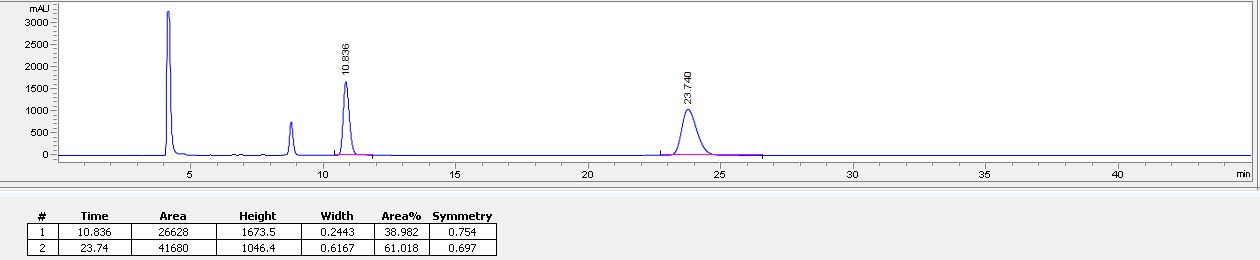


Asymmetric Mukaiyama-Mannich reaction with aldimine **2a** and ketene silyl acetal catalyzed by CPA **1b (50R+50S)** at – 78 °C and 10% catalyst loading in CD_2_Cl_2_.


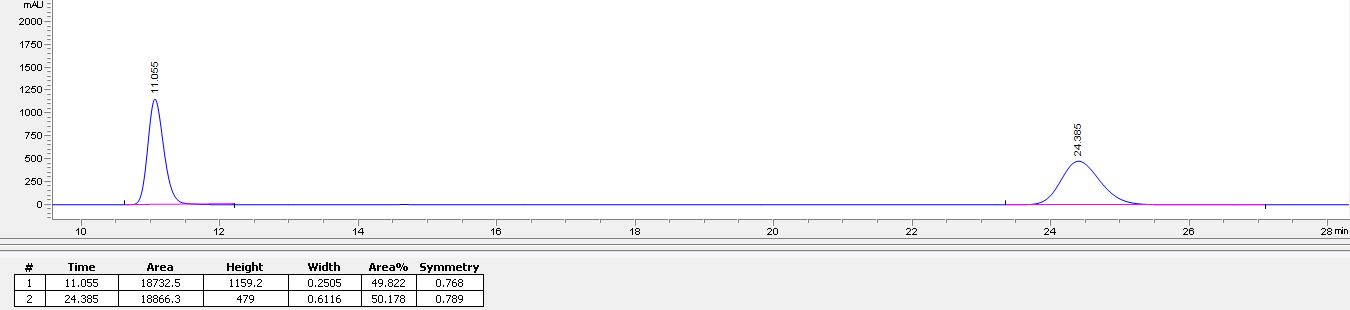


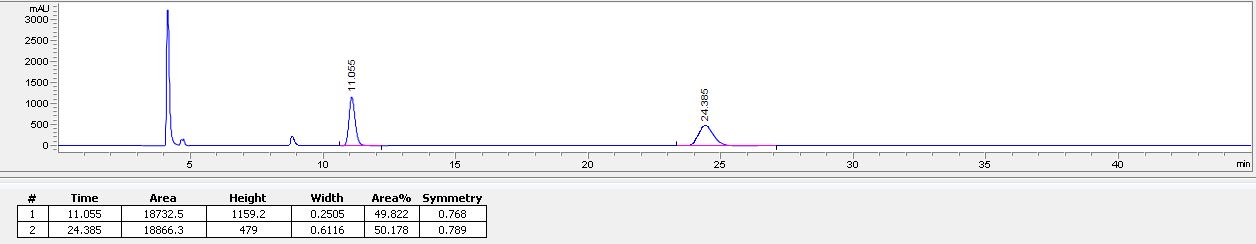


Asymmetric Mukaiyama-Mannich reaction with aldimine **2a** and ketene silyl acetal catalyzed by CPA **1b (40R+60S)** at – 78 °C and 10% catalyst loading in CD_2_Cl_2_.


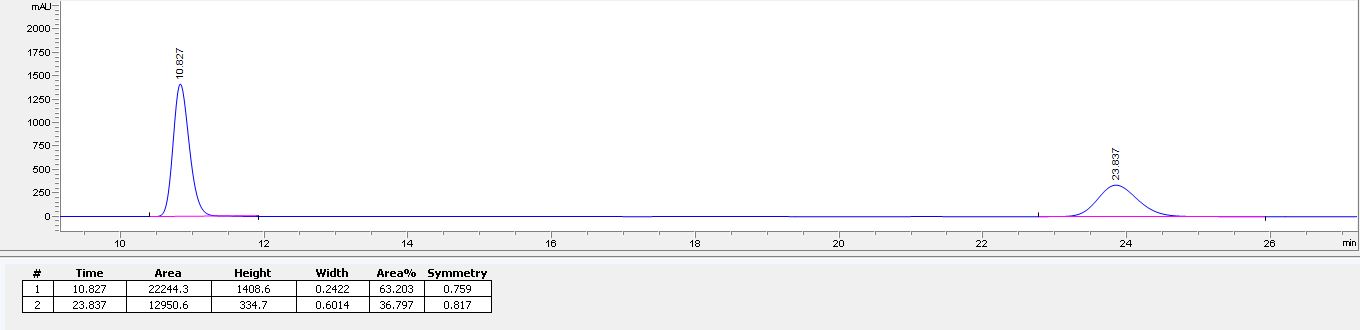


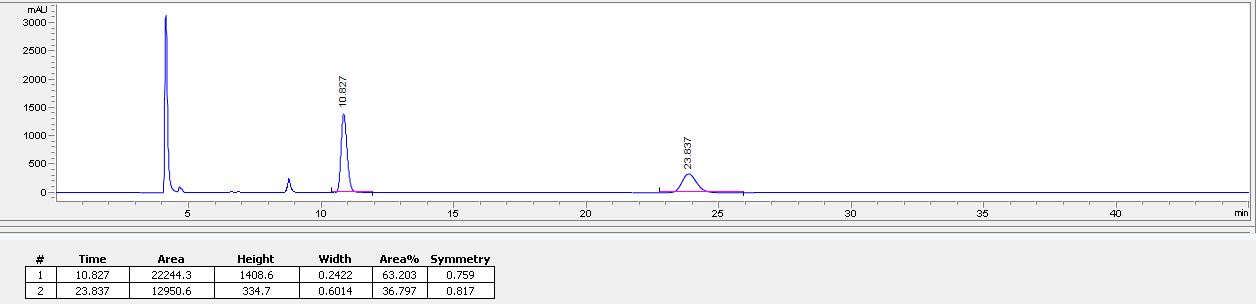


Asymmetric Mukaiyama-Mannich reaction with aldimine **2a** and ketene silyl acetal catalyzed by CPA **1b (30R+70S)** at – 78 °C and 10% catalyst loading in CD_2_Cl_2_.


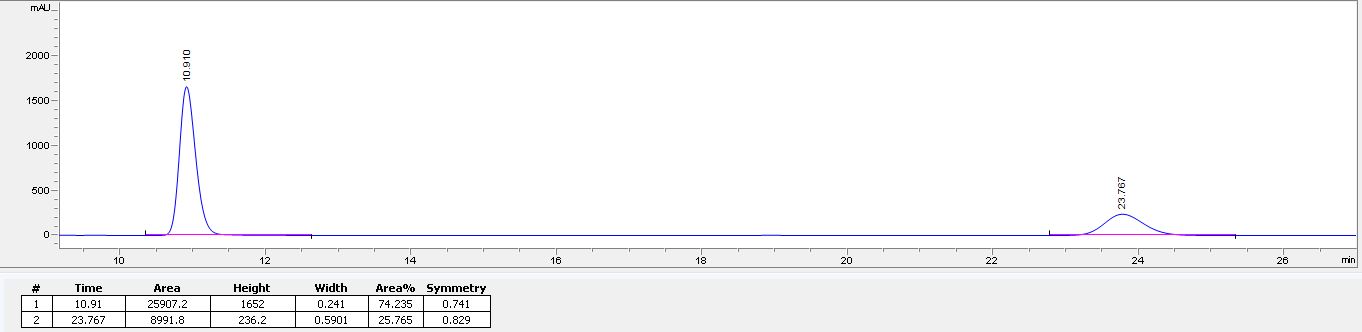


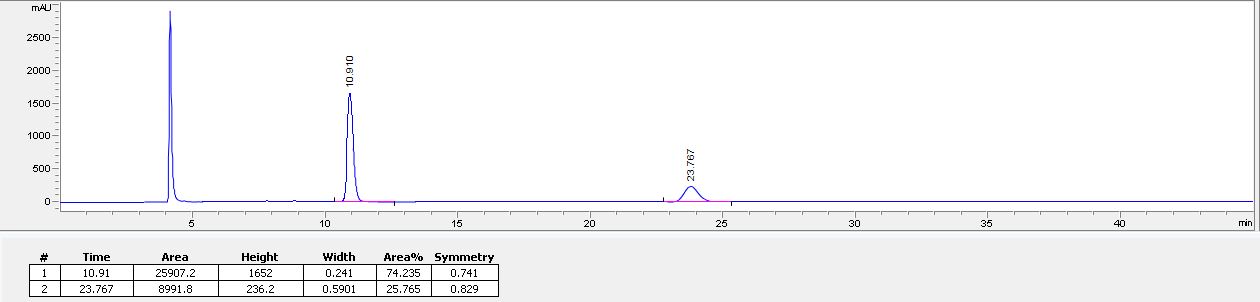


Asymmetric Mukaiyama-Mannich reaction with aldimine **2a** and ketene silyl acetal catalyzed by CPA **1b (20R+80S)** at – 78 °C and 10% catalyst loading in CD_2_Cl_2_.


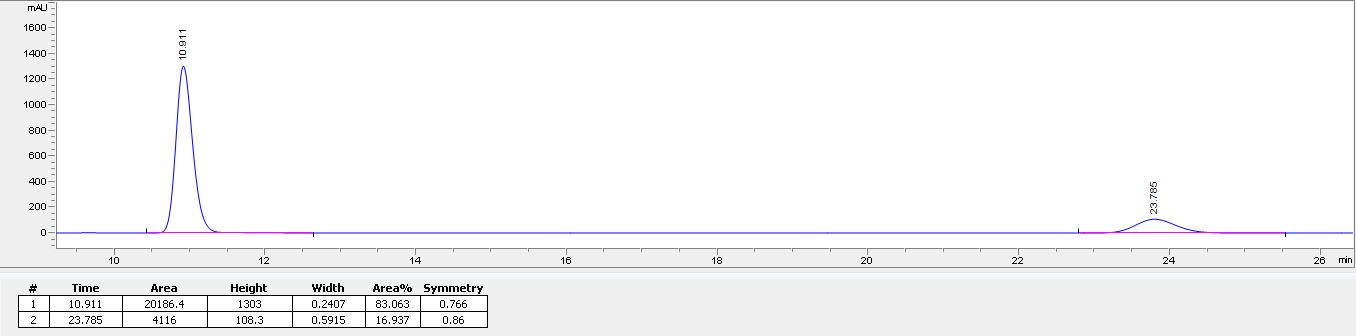


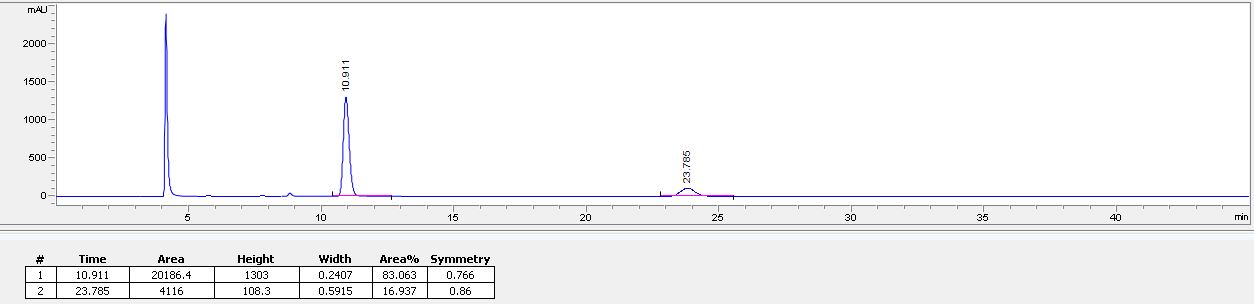


Asymmetric Mukaiyama-Mannich reaction with aldimine **2a** and ketene silyl acetal catalyzed by CPA **1b (10R+90S)** at – 78 °C and 10% catalyst loading in CD_2_Cl_2_.


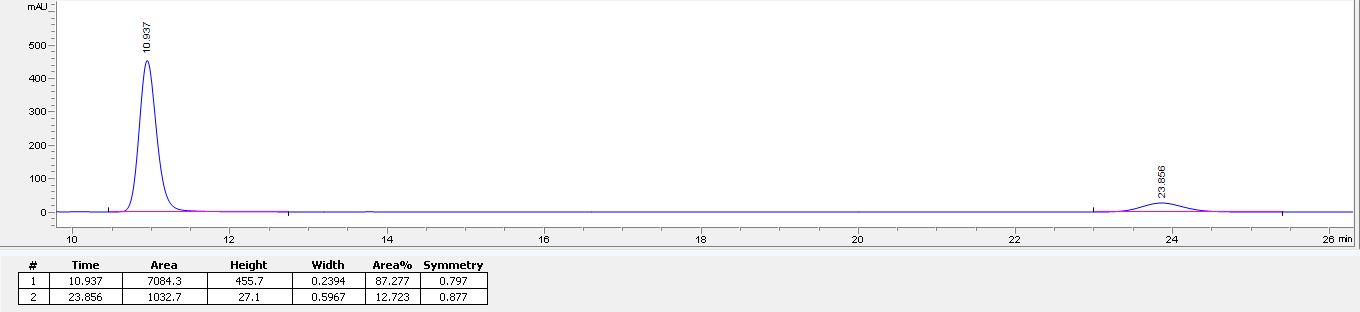


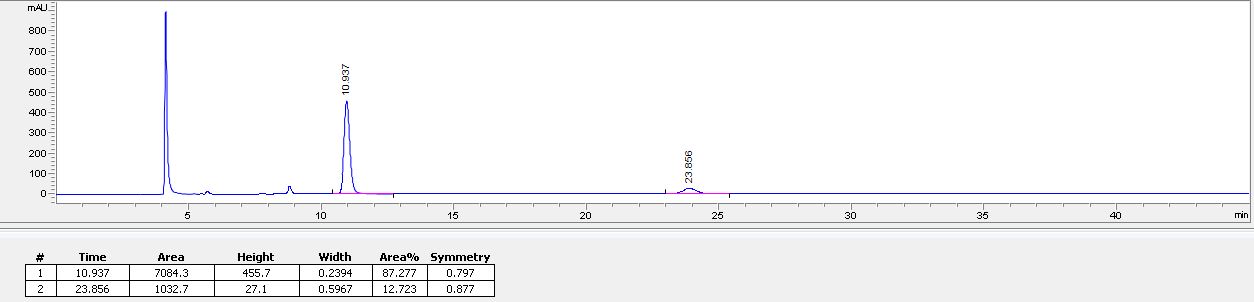


Asymmetric Mukaiyama-Mannich reaction with aldimine **2a** and ketene silyl acetal catalyzed by CPA **1b (100S)** at – 78 °C and 10% catalyst loading in CD_2_Cl_2_.


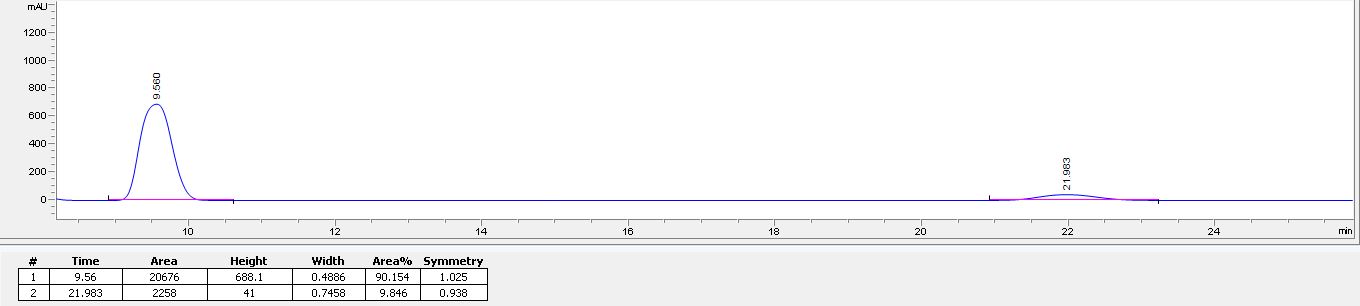


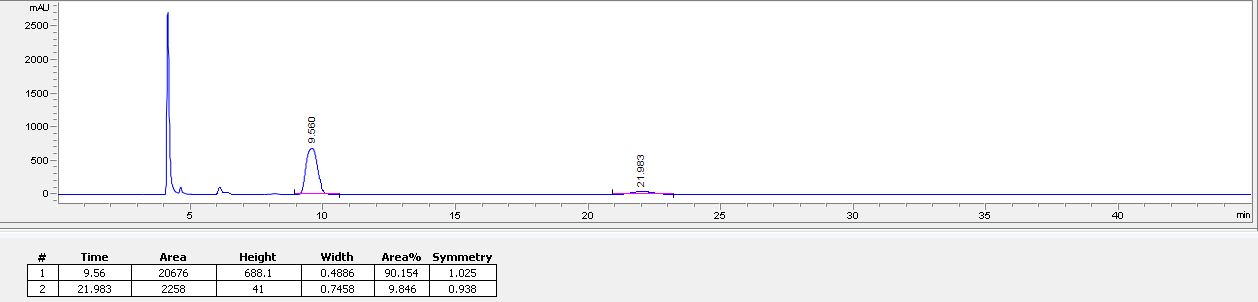


Asymmetric Mukaiyama-Mannich reaction with aldimine **2c** and ketene silyl acetal catalyzed by CPA **1b (100R)** at – 78 °C and 10% catalyst loading in toluene.


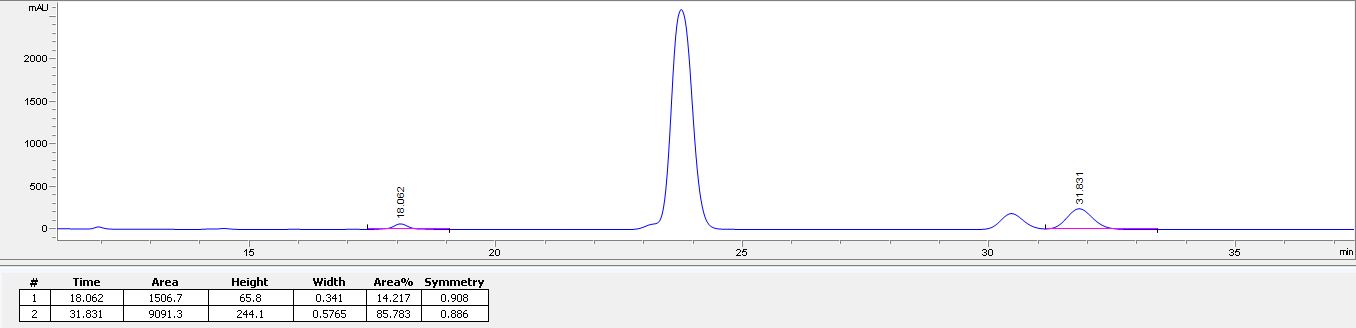


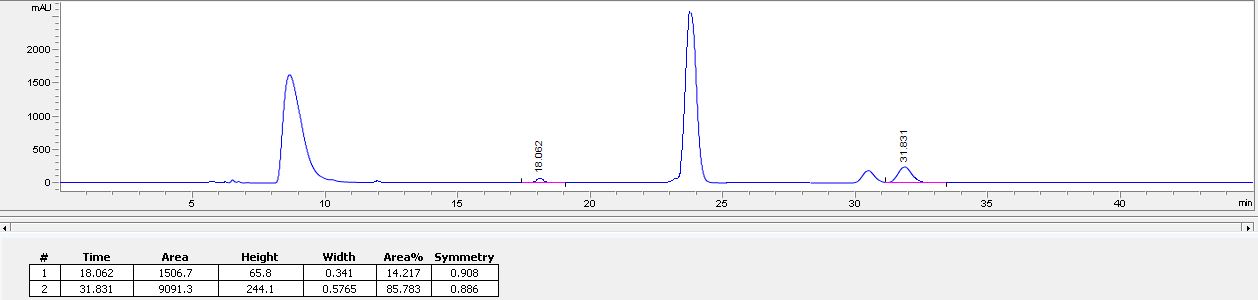


Asymmetric Mukaiyama-Mannich reaction with aldimine **2c** and ketene silyl acetal catalyzed by CPA **1b (90R+10S)** at – 78 °C and 10% catalyst loading in toluene.


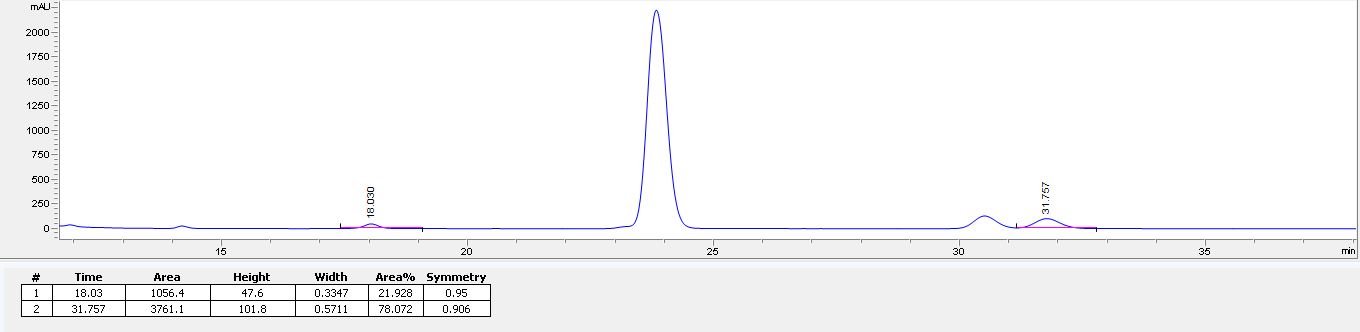


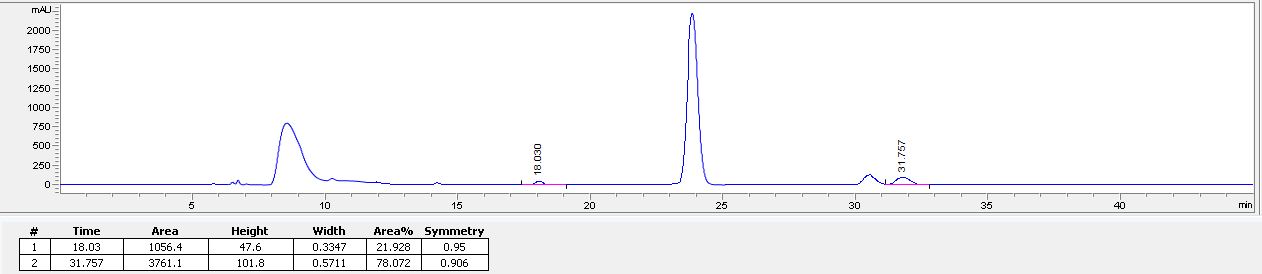


Asymmetric Mukaiyama-Mannich reaction with aldimine **2c** and ketene silyl acetal catalyzed by CPA **1b (80R+20S)** at – 78 °C and 10% catalyst loading in toluene.


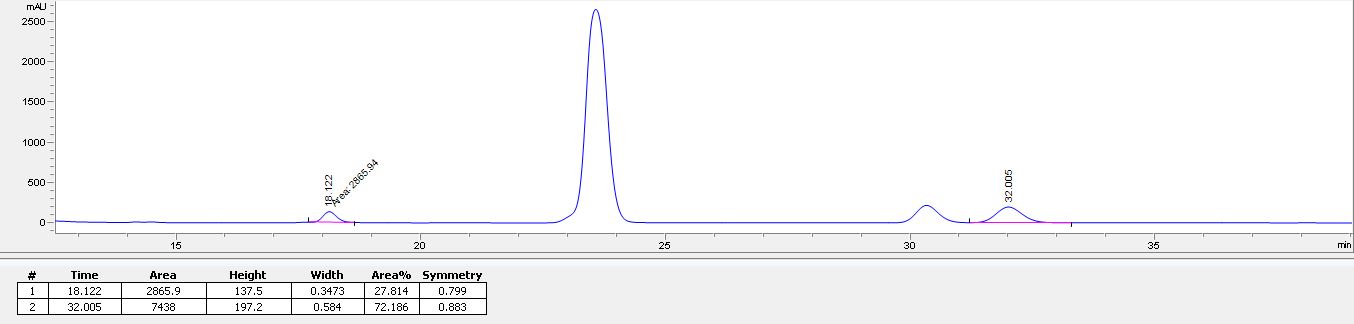


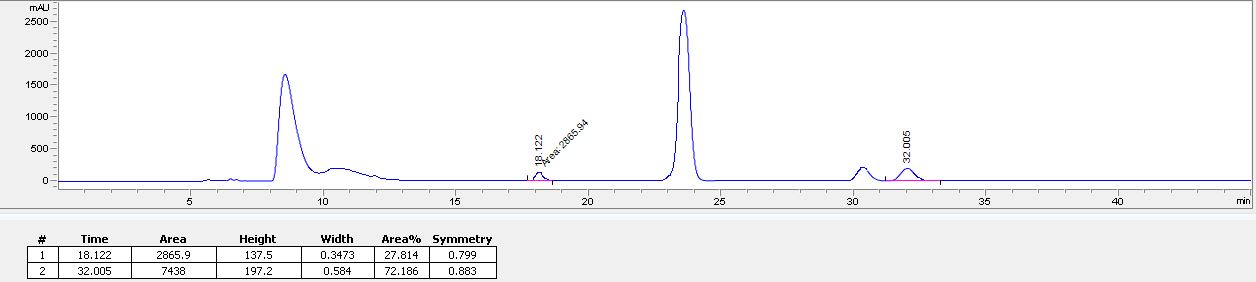


Asymmetric Mukaiyama-Mannich reaction with aldimine **2c** and ketene silyl acetal catalyzed by CPA **1b (70R+30S)** at – 78 °C and 10% catalyst loading in toluene.


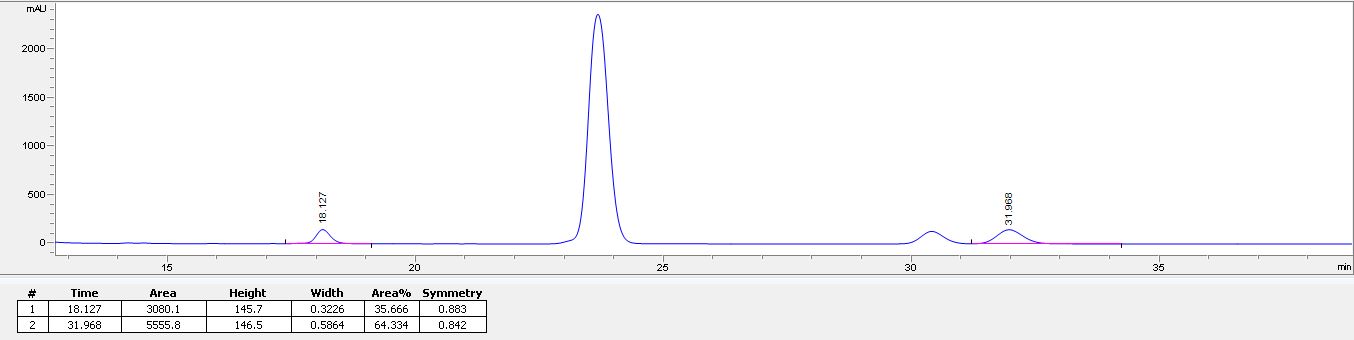


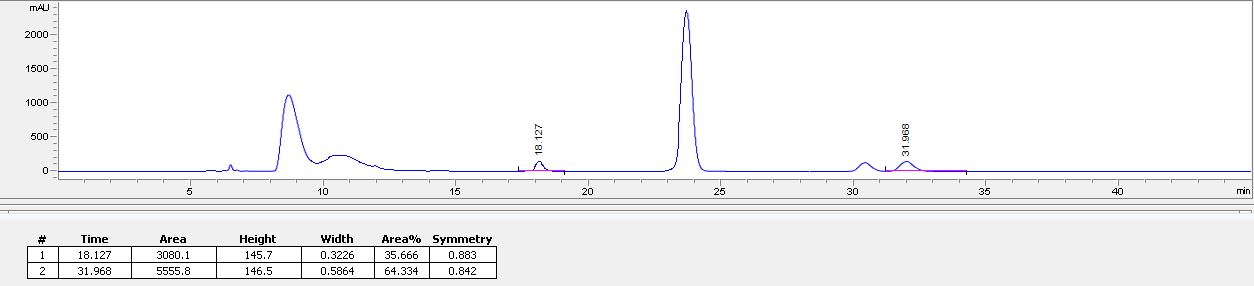


Asymmetric Mukaiyama-Mannich reaction with aldimine **2c** and ketene silyl acetal catalyzed by CPA **1b (60R+40S)** at – 78 °C and 10% catalyst loading in toluene.


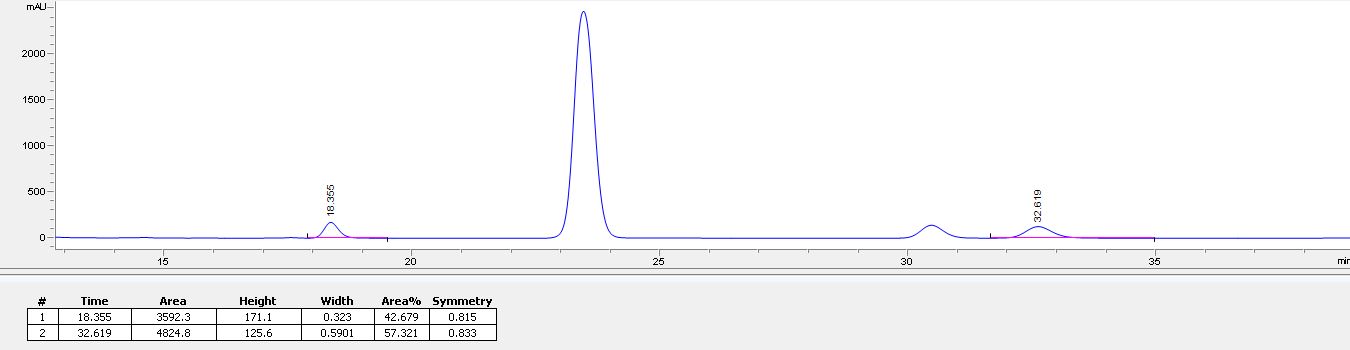


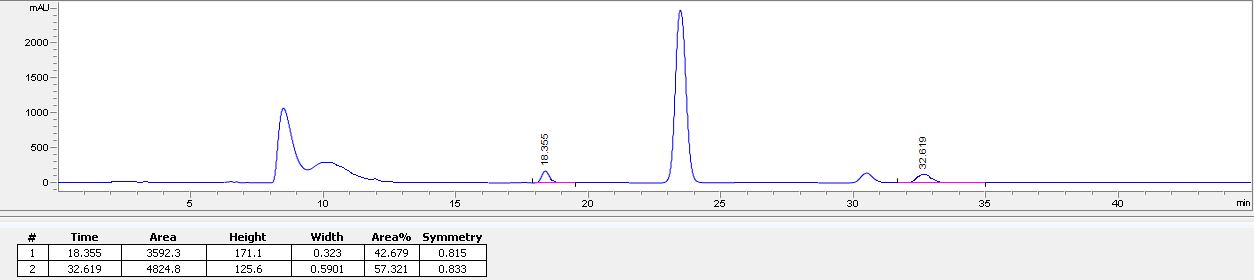


Asymmetric Mukaiyama-Mannich reaction with aldimine **2c** and ketene silyl acetal catalyzed by CPA **1b (50R+50S)** at – 78 °C and 10% catalyst loading in toluene.


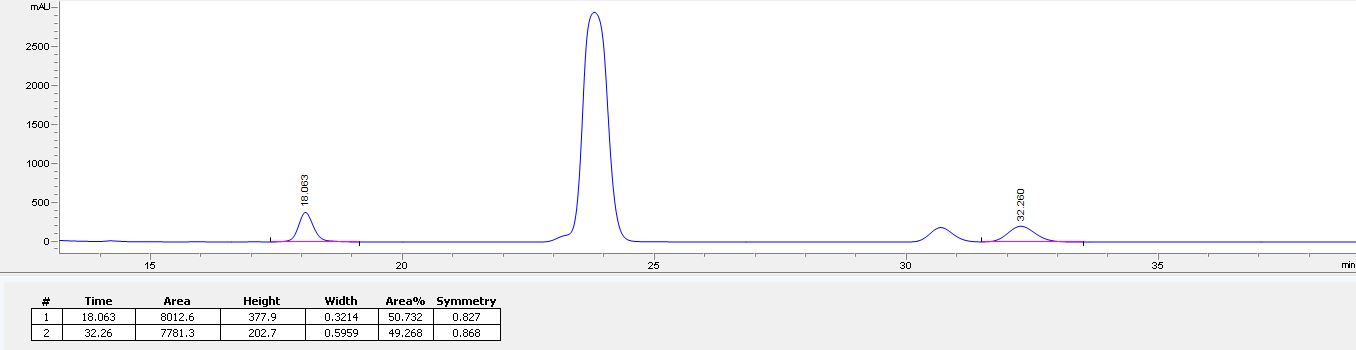


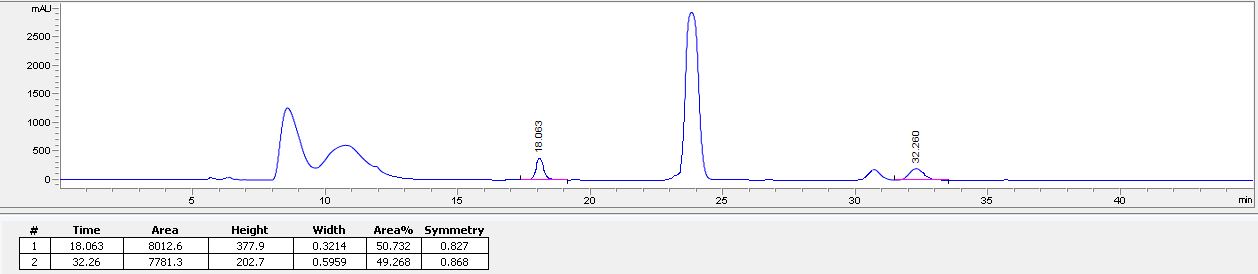


Asymmetric Mukaiyama-Mannich reaction with aldimine **2a** and ketene silyl acetal catalyzed by CPA **1c (100R)** at – 78 °C and 10% catalyst loading in toluene.


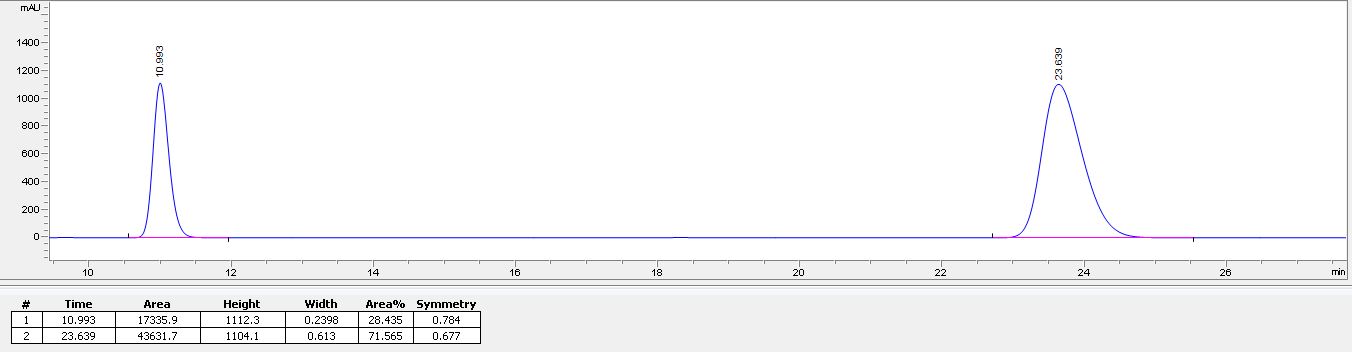


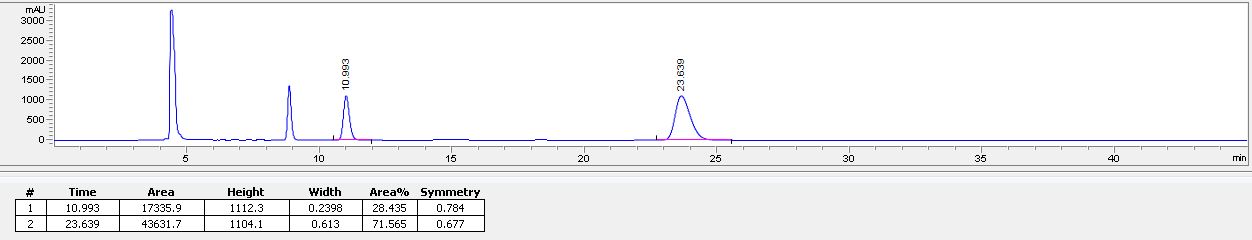


Asymmetric Mukaiyama-Mannich reaction with aldimine **2a** and ketene silyl acetal catalyzed by CPA **1c (90R+10S)** at – 78 °C and 10% catalyst loading in toluene.


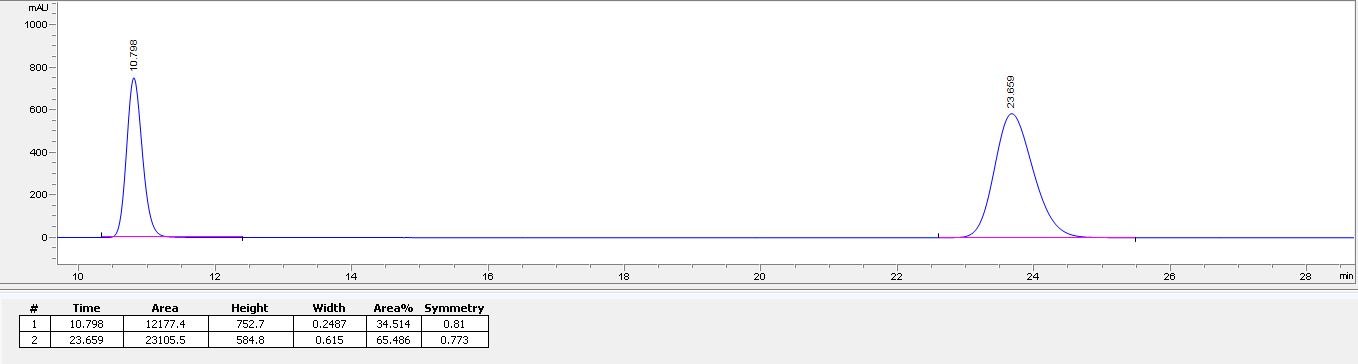


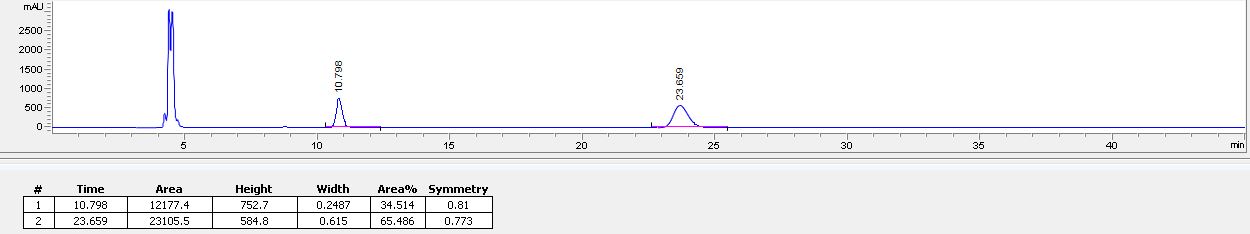


Asymmetric Mukaiyama-Mannich reaction with aldimine **2a** and ketene silyl acetal catalyzed by CPA **1c (80R+20S)** at – 78 °C and 10% catalyst loading in toluene.


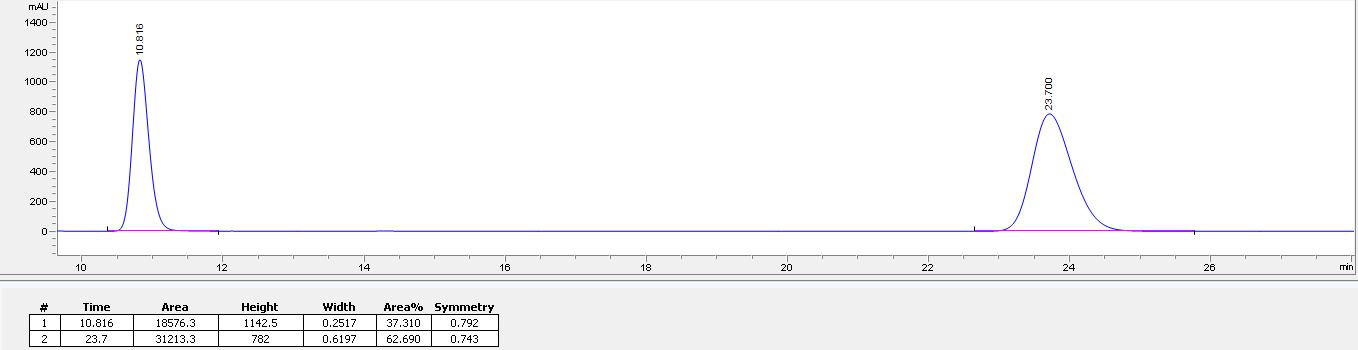


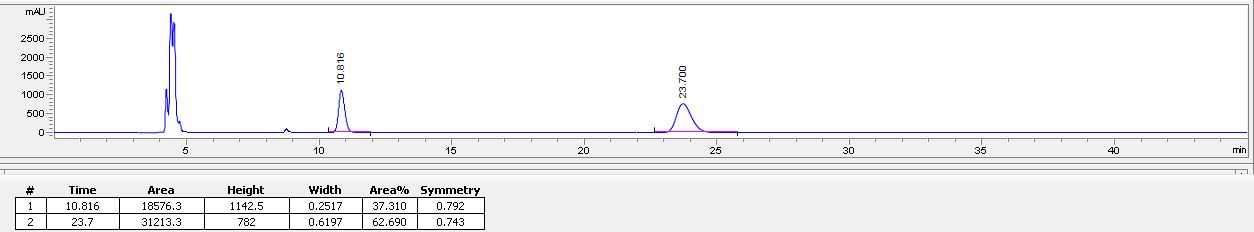


Asymmetric Mukaiyama-Mannich reaction with aldimine **2a** and ketene silyl acetal catalyzed by CPA **1c (70R+30S)** at – 78 °C and 10% catalyst loading in toluene.


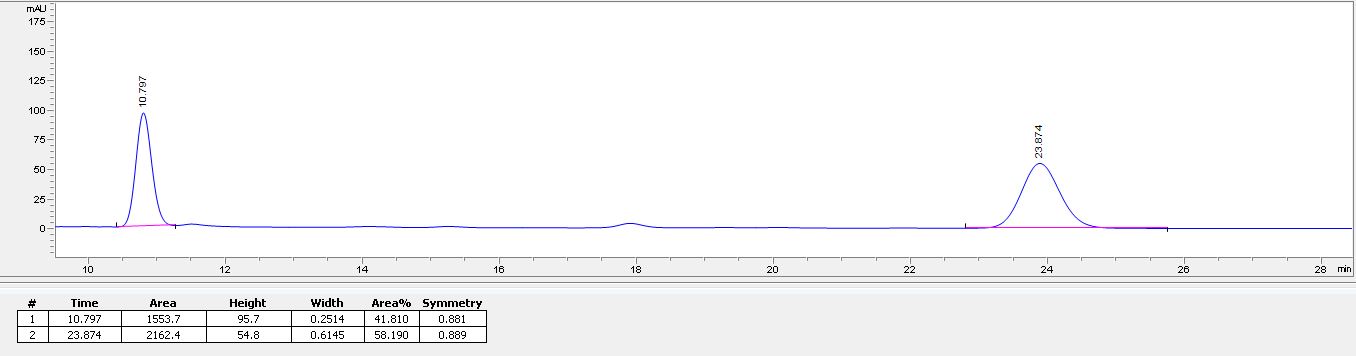


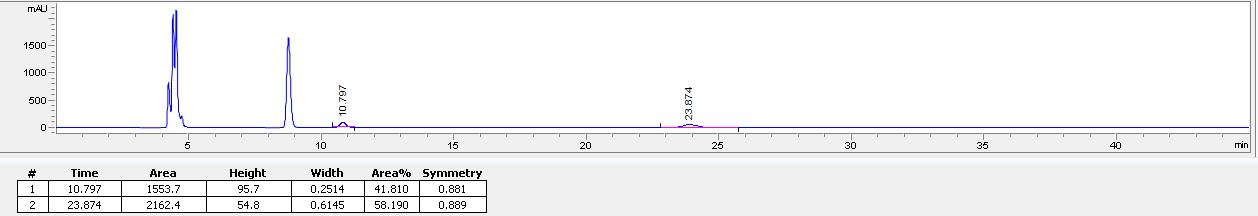


Asymmetric Mukaiyama-Mannich reaction with aldimine **2a** and ketene silyl acetal catalyzed by CPA **1c (60R+40S)** at – 78 °C and 10% catalyst loading in toluene.


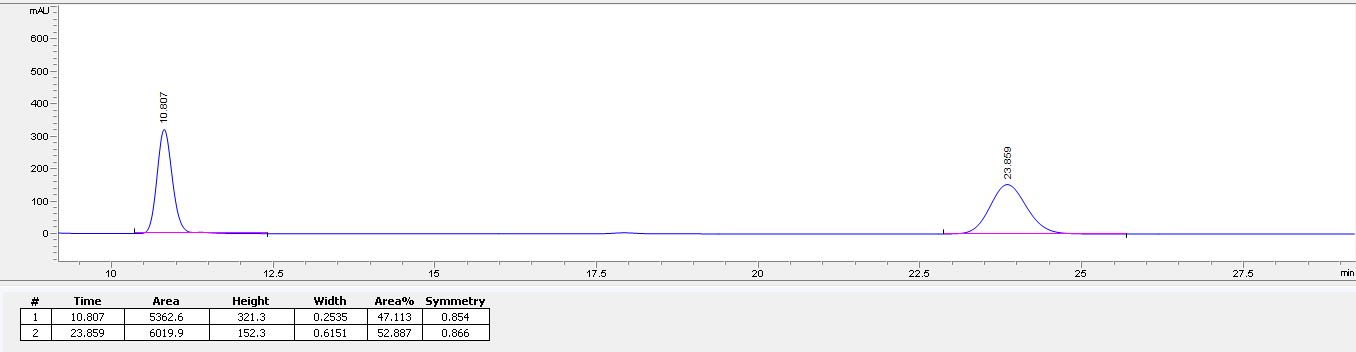


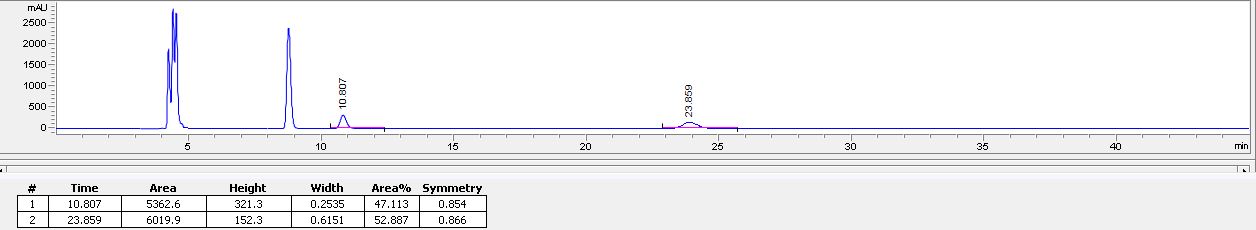


Asymmetric Mukaiyama-Mannich reaction with aldimine **2a** and ketene silyl acetal catalyzed by CPA **1c (50R+50S)** at – 78 °C and 10% catalyst loading in toluene.


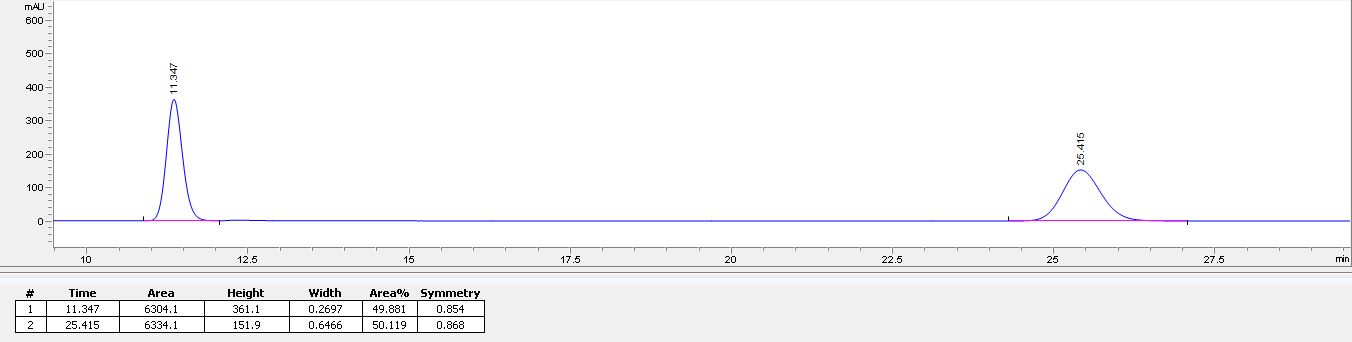


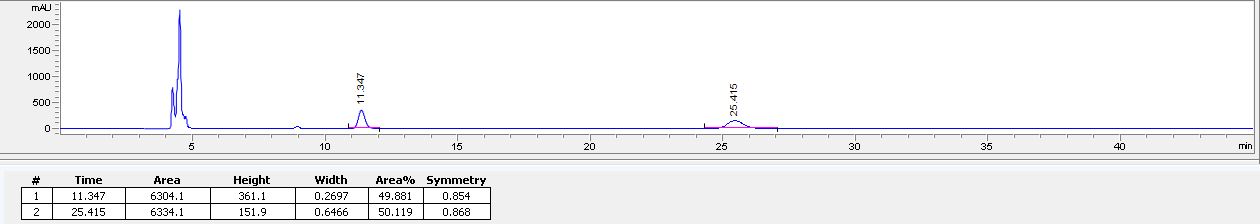


Asymmetric Mukaiyama-Mannich reaction with aldimine **2b** and ketene silyl acetal catalyzed by CPA **1b** at – 78 °C and 10% catalyst loading in CD_2_Cl_2_.


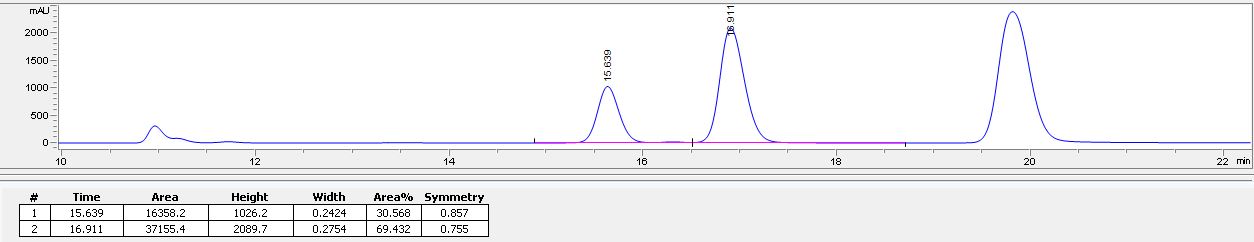


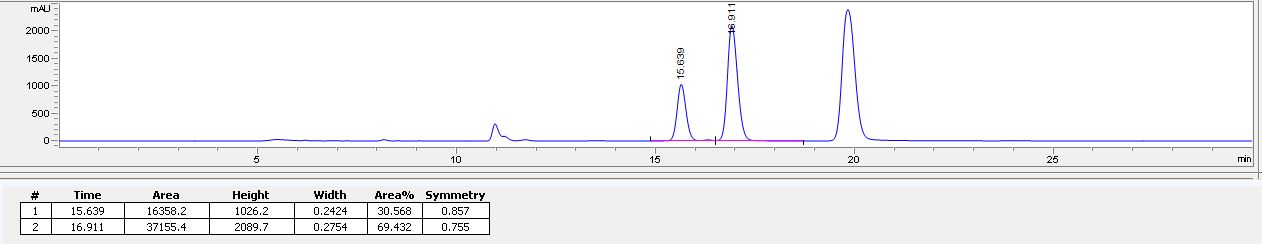


Asymmetric Mukaiyama-Mannich reaction with aldimine **2c** and ketene silyl acetal catalyzed by CPA **1b** at – 78 °C and 10% catalyst loading in toluene.


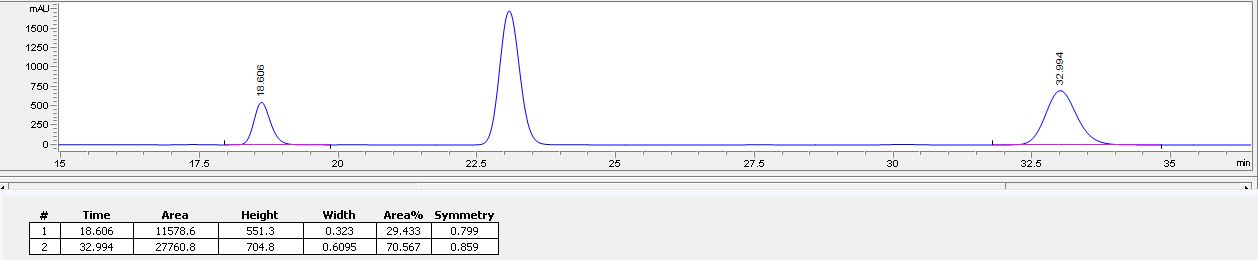


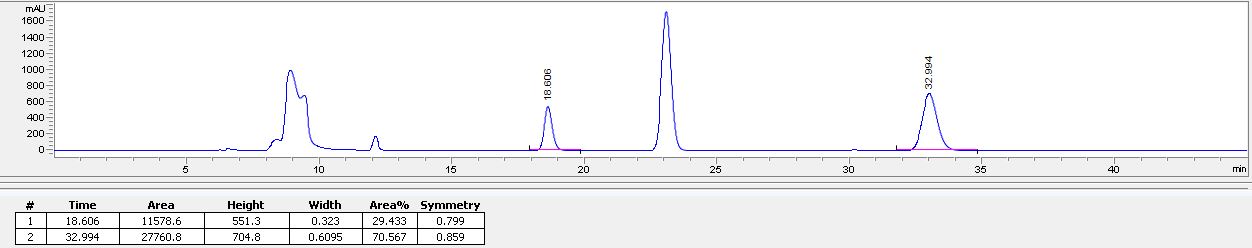


# NMR Spectroscopic Investigations

### Acid/Imine Systems


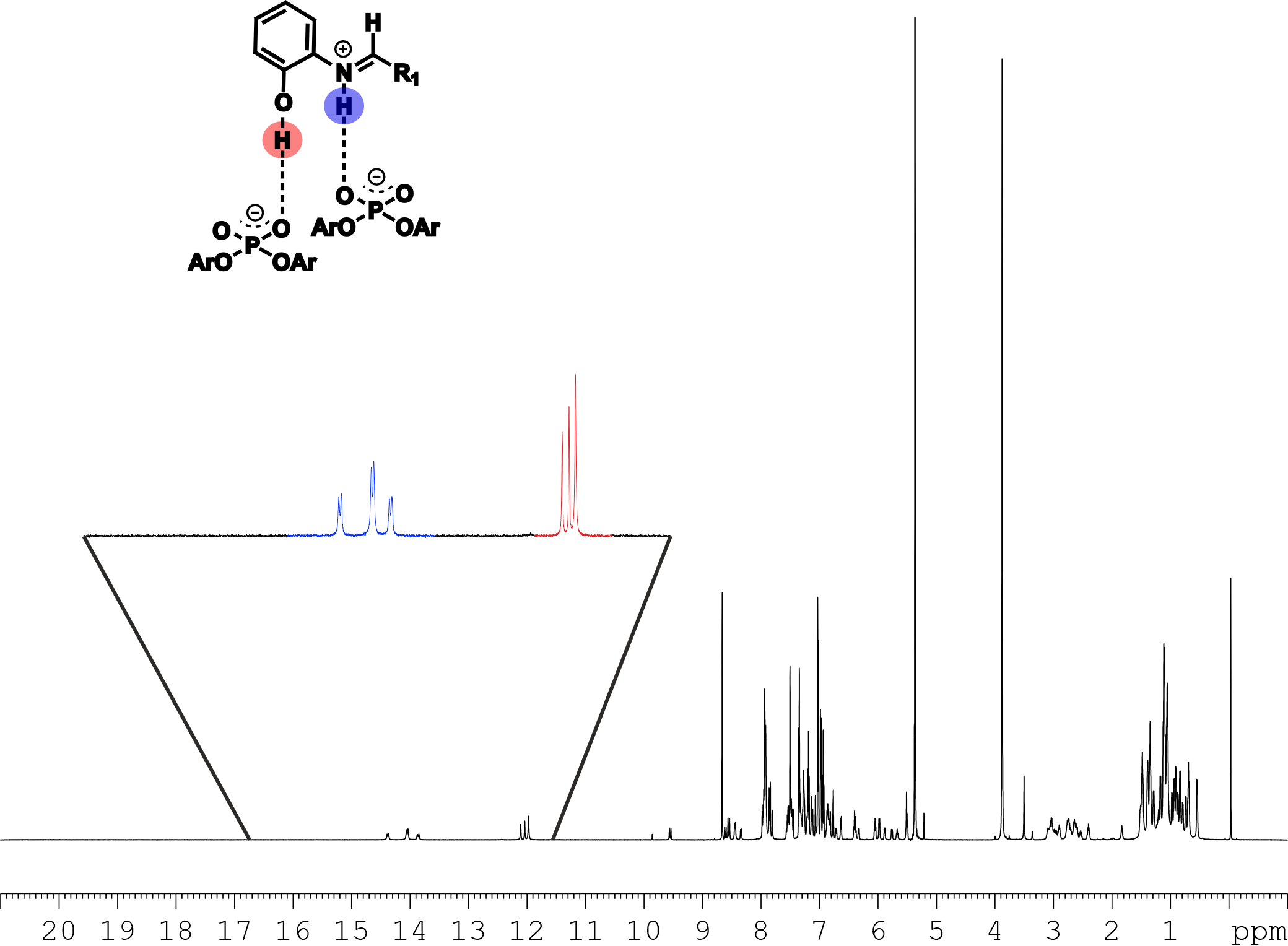


Figure S1: ^1^H spectrum of **1d**/**2a** at a 1:1 ratio and a concentration of 10 mM respectively in CD_2_Cl_2_ at 600 MHz at 193 K. The spectra of **1d/2a** was reproduced from previous data to show the basis for the structural elucidation of the [CPA/imine]_2_ dimers.(1) The presence of well separated signals in the H-bond region enabled a detailed NMR investigation revealing three dimeric [CPA/imine]_2_ structures. However, this study focuses on the catalytically active CPA catalysts **1a** and **1b** to evaluate how the formation of [CPA/imine]_2_ dimers influence the reaction outcome. A detailed structural elucidation of these catalysts was not possible as the H-bond region appears significantly broadened. Still, two key regions characteristic for the aggregated species could be monitored: Signals around 14 ppm were assigned to the POHN hydrogen bonds of the dimers. Signals at approximately 12 ppm were attributed to the POHO hydrogen bonds of the dimer.


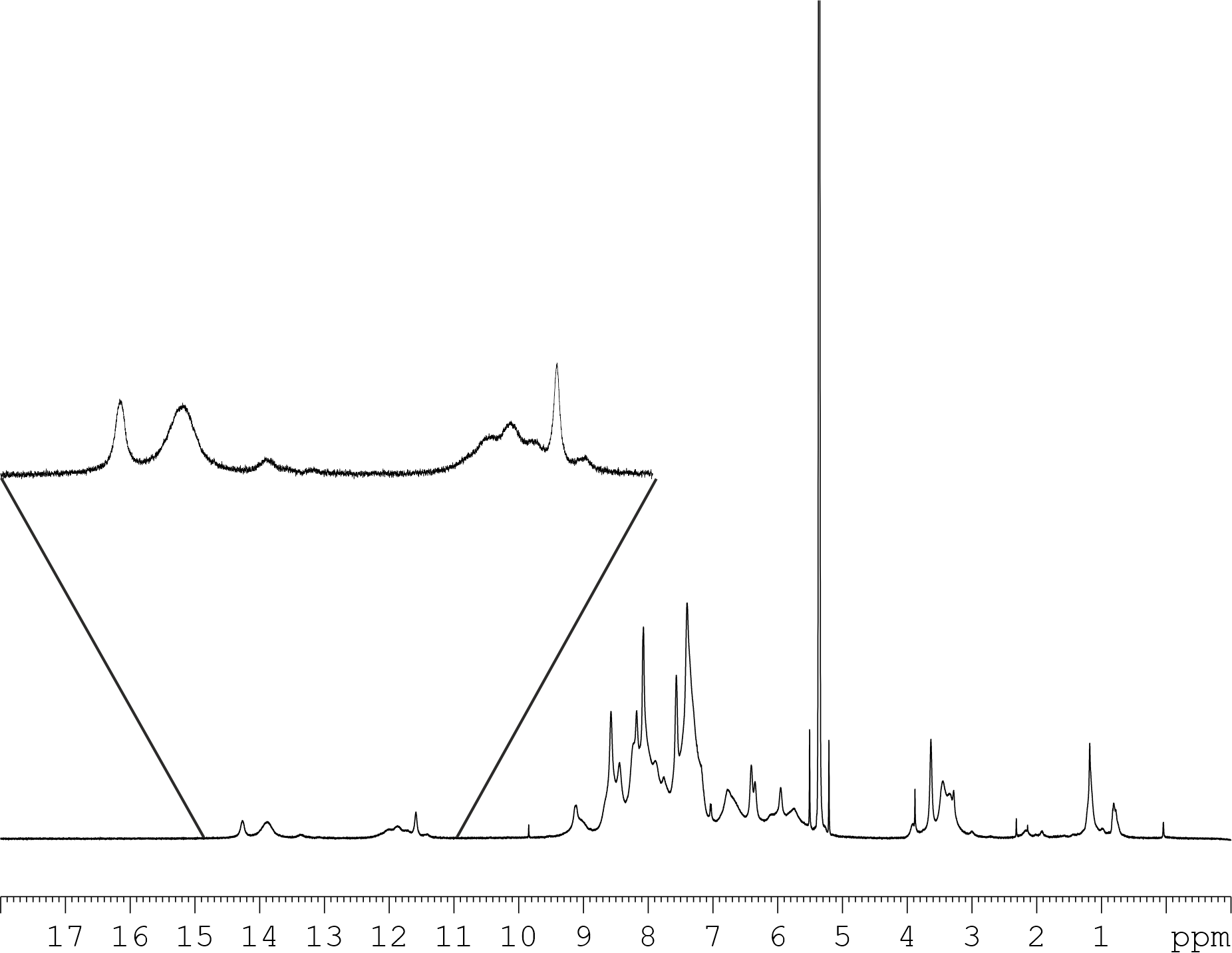


Figure S2: ^1^H spectrum of **1b/2a** at 193 K in a 1:1 ratio (600 MHz, CD_2_Cl_2_).


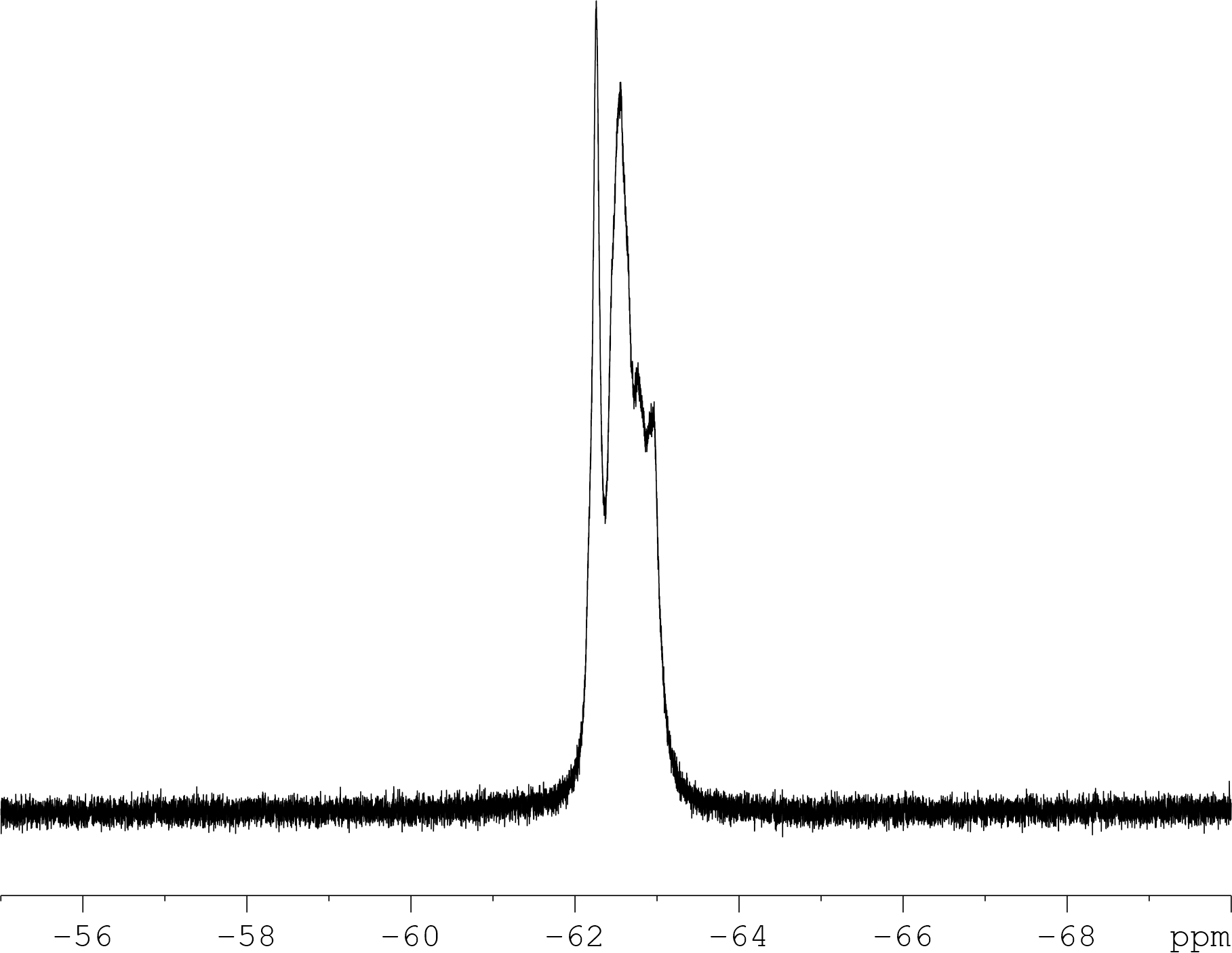


Figure S3: ^19^F spectrum of **1b/2a** at 193 K in a 1:1 ratio (600 MHz, CD_2_Cl_2_).


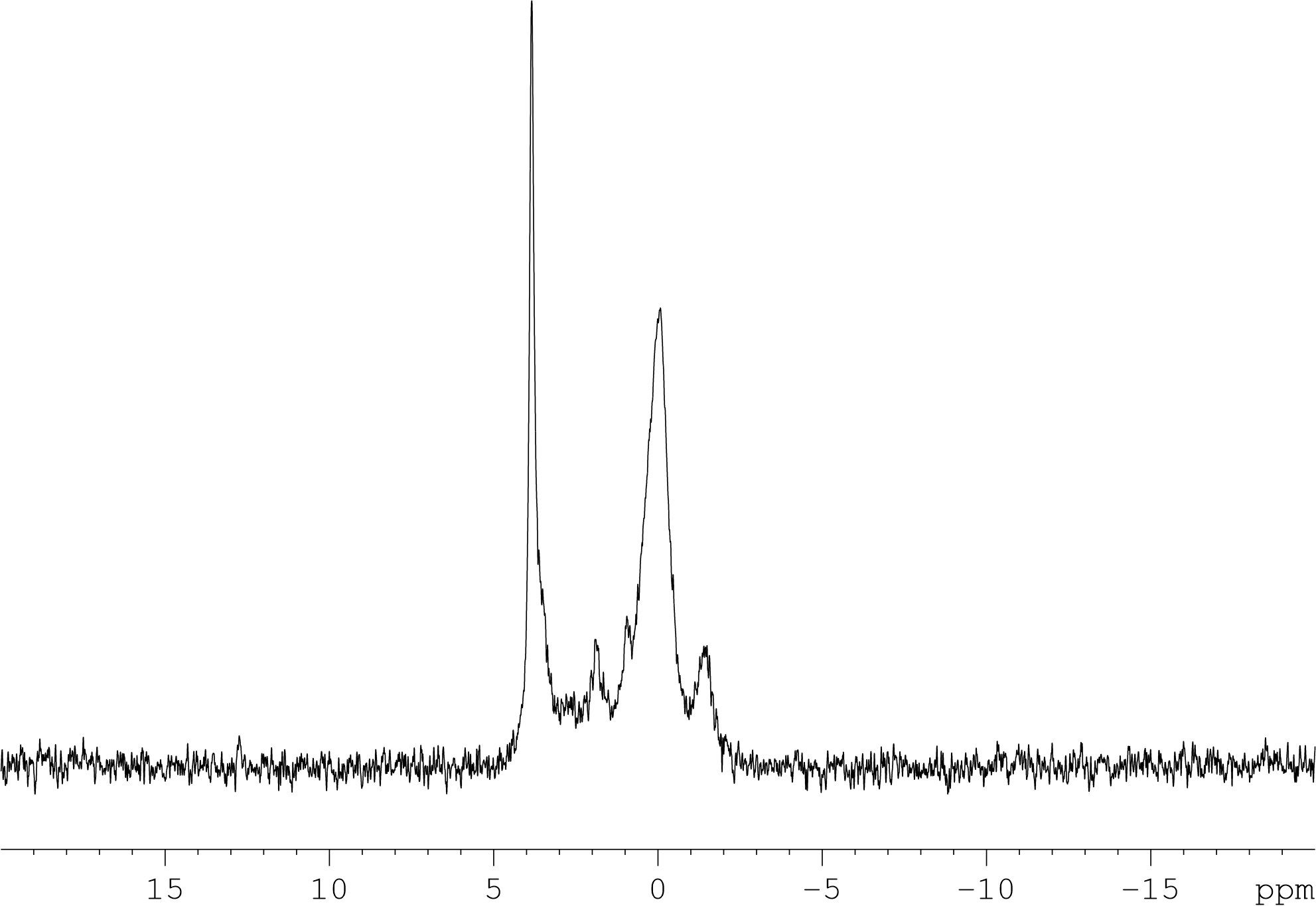


Figure S4: ^31^P spectrum of **1b/2a** at 193 K in a 1:1 ratio (600 MHz, CD_2_Cl_2_).


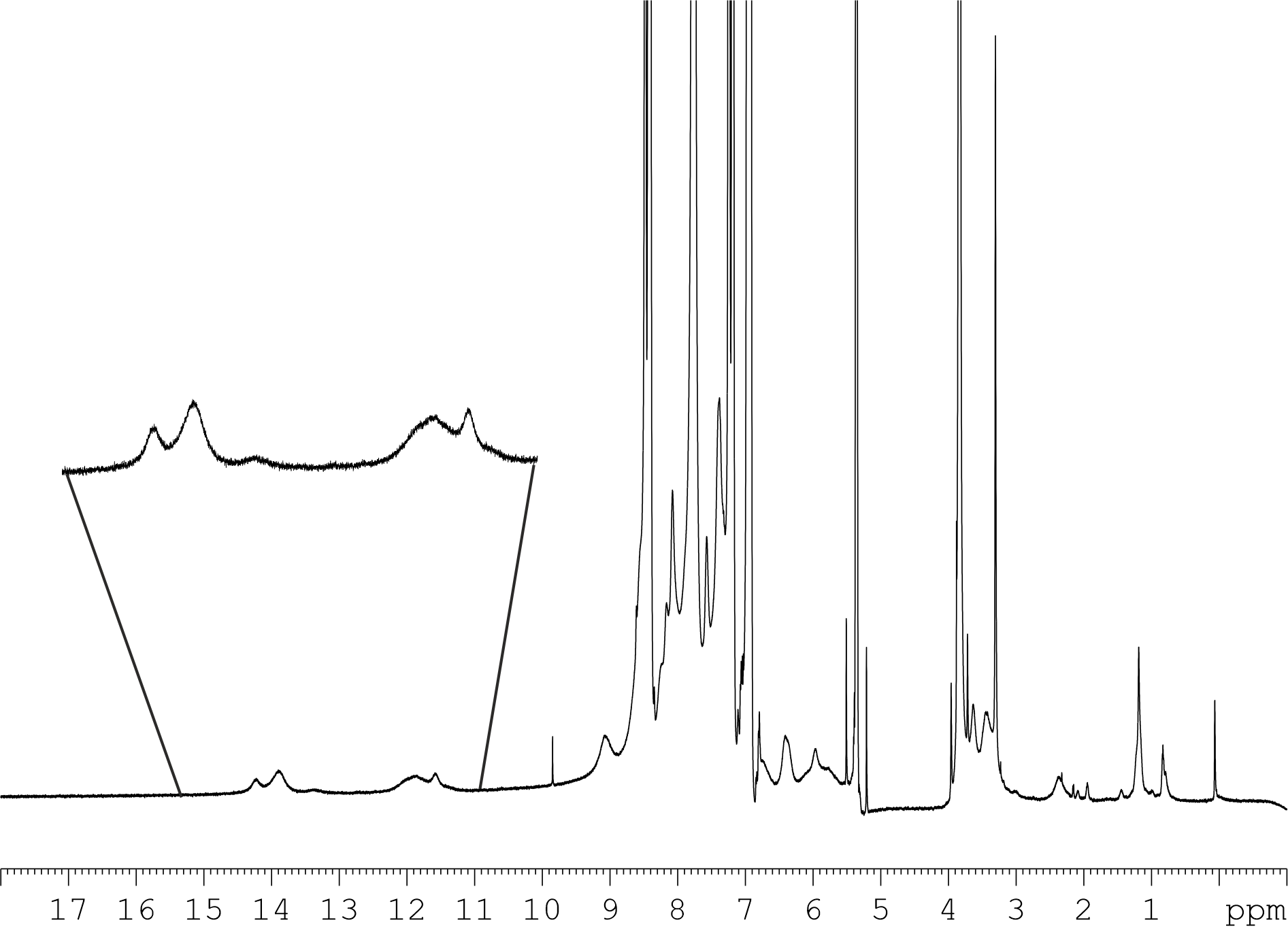


Figure S5: ^1^H spectrum of **1b/2a** at 193 K in a 1:10 ratio (600 MHz, CD_2_Cl_2_).

Figure S6: ^19^F spectrum of **1b/2a** at 193 K in a 1:10 ratio (600 MHz, CD_2_Cl_2_).

Figure S7: ^31^P spectrum of **1b/2a** at 193 K in a 1:10 ratio (600 MHz, CD_2_Cl_2_).

Figure S8: ^1^H spectrum of **1b/2b** at 193 K in a 1:1 ratio (600 MHz, CD_2_Cl_2_).

Figure S9: ^19^F spectrum of **1b/2b** at 193 K in a 1:1 ratio (600 MHz, CD_2_Cl_2_).

Figure S10: ^31^P spectrum of **1b/2b** at 193 K in a 1:1 ratio (600 MHz, CD_2_Cl_2_).

Figure S11: ^1^H spectrum of **1b/2b** at 193 K in a 1:10 ratio (600 MHz, CD_2_Cl_2_).

Figure S12: ^19^F spectrum of **1b/2b** at 193 K in a 1:10 ratio (600 MHz, CD_2_Cl_2_).

Figure S13: ^31^P spectrum of **1b/2b** at 193 K in a 1:10 ratio (600 MHz, CD_2_Cl_2_).

Figure S14: ^1^H spectrum of **1b/2c** at 193 K in a 1:1 ratio (600 MHz, CD_2_Cl_2_).

Figure S15: ^19^F spectrum of **1b/2c** at 193 K in a 1:1 ratio (600 MHz, CD_2_Cl_2_).

Figure S16: ^31^P spectrum of **1b/2c** at 193 K in a 1:1 ratio (600 MHz, CD_2_Cl_2_).

Figure S17: ^1^H spectrum of **1b/2c** at 193 K in a 1:10 ratio (600 MHz, CD_2_Cl_2_).

Figure S18: ^19^F spectrum of **1b/2c** at 193 K in a 1:10 ratio (600 MHz, CD_2_Cl_2_).

Figure S19: ^31^P spectrum of **1b/2c** at 193 K in a 1:10 ratio (600 MHz, CD_2_Cl_2_).

Figure S20: ^1^H spectrum of **1c/2a** at 193 K in a 1:1 ratio (600 MHz, CD_2_Cl_2_).

Figure S21: ^19^F spectrum of **1c/2a** at 193 K in a 1:1 ratio (600 MHz, CD_2_Cl_2_).

Figure S22: ^31^P spectrum of **1c/2a** at 193 K in a 1:1 ratio (600 MHz, CD_2_Cl_2_).

Figure S23: ^1^H spectrum of **1c/2a** at 193 K in a 1:10 ratio (600 MHz, CD_2_Cl_2_).

Figure S24: ^19^F spectrum of **1c/2a** at 193 K in a 1:10 ratio (600 MHz, CD_2_Cl_2_).

Figure S25: ^31^P spectrum of **1c/2a** at 193 K in a 1:10 ratio (600 MHz, CD_2_Cl_2_).

Figure S26: ^1^H spectrum of **1c/2b** at 193 K in a 1:1 ratio (600 MHz, CD_2_Cl_2_).

Figure S27: ^19^F spectrum of **1c/2b** at 193 K in a 1:1 ratio (600 MHz, CD_2_Cl_2_).

Figure S28: ^31^P spectrum of **1c/2b** at 193 K in a 1:1 ratio (600 MHz, CD_2_Cl_2_).

Figure S29: ^1^H spectrum of **1c/2b** at 193 K in a 1:10 ratio (600 MHz, CD_2_Cl_2_).

Figure S30: ^19^F spectrum of **1c/2b** at 193 K in a 1:10 ratio (600 MHz, CD_2_Cl_2_).

Figure S31: ^31^P spectrum of **1c/2c** at 193 K in a 1:10 ratio (600 MHz, CD_2_Cl_2_).

Figure S32: ^1^H spectrum of **1c/2c** at 193 K in a 1:1 ratio (600 MHz, CD_2_Cl_2_).

Figure S33: ^19^F spectrum of **1c/2c** at 193 K in a 1:1 ratio (600 MHz, CD_2_Cl_2_).

Figure S34: ^31^P spectrum of **1c/2c** at 193 K in a 1:1 ratio (600 MHz, CD_2_Cl_2_).

Figure S35: ^1^H spectrum of **1c/2c** at 193 K in a 1:10 ratio (600 MHz, CD_2_Cl_2_).

Figure S36: ^19^F spectrum of **1c/2c** at 193 K in a 1:10 ratio (600 MHz, CD_2_Cl_2_).

Figure S37: ^31^P spectrum of **1c/2c** at 193 K in a 1:10 ratio (600 MHz, CD_2_Cl_2_).

Figure S38: ^1^H spectrum of **1a/2a** at 193 K in a 1:1 ratio (600 MHz, CD_2_Cl_2_).

Figure S39: ^31^P spectrum of **1a/2a** at 193 K in a 1:1 ratio (600 MHz, CD_2_Cl_2_).

Figure S40: ^1^H spectrum of **1a/2a** at 193 K in a 1:10 ratio (600 MHz, CD_2_Cl_2_).

Figure S41: ^31^P spectrum of **1a/2a** at 193 K in a 1:10 ratio (600 MHz, CD_2_Cl_2_).

Figure S42: ^1^H spectrum of **1b/2d** at 193 K (600 MHz, CD_2_Cl_2_). The exact ratio and concentration could not be determined due to the insolubility of the imine, even at concentrations as low as 1 mM.

### Diffusion Ordered Spectroscopy (DOSY)

All DOSY measurements were performed with the convection suppressing DSTE (double stimulated echo) pulse sequence developed by Jerschow and Müller in a pseudo 2D mode.(*7*) Therefore, TMS was added to the sample and used as reference for the ^1^H chemical shifts and for temperature as well as the viscosity of the solvent. For the measurement a set of 4 dummy scans and 8-32 scans was used for all samples. A relaxation delay between 1.5 and 3.0 s were used for all samples. The diffusion time delay and the gradient pulse lengths (d20, p30, SINE.100 pulse shape or SMSQ10.100 pulse shape) were optimized for each species to give a sigmoidal signal decay for varying gradient strengths between 5% and 95%. For each species, 32 spectra with linear varying gradient strength from 5% to 95% were measured. For integration, all separated signals were used for each substrate. The signal intensities of these groups in the DOSY spectra were classically analyzed as a function of the gradient strength by the in Bruker TopSpin 3.2 included software T1/T2 relaxation package by employing the Stejskal-Tanner equation.(*8*) Based on the obtained translational diffusion coefficients, the hydrodynamic radii of the analytes *r_H_* were estimated following the Stokes-Einstein equation, with *D_i_* = self-diffusion coefficient, *k* = Boltzmann constant, *T* = temperature, *η* = viscosity of the sample, *c* = correcting factor, *F* = shape factor:(*9*)

$$D_{i}=\frac{k_{B}T}{Fc\pi\eta r_{H}}$$

The shape factor *F* was set to 1 for a spherical shape. The semi-empirical modification by Chen was used to calculate the correction *c*. Therefore, from literature known value for the radius of the corresponding solvent was used (r_CD2Cl2_ = 2.46 Å).(*10, 11*)

$$c_{chen}=\frac{6F}{1+0.695{(\frac{r_{solv}}{r_{ref}})}^{2.234}}$$

Viscosity calibration of the derived *Di* values was performed with literature known values for the radii of TMS (*r_ref_* = 2.96 Å, calculated from hard-sphere increments(*12*)) and the experimentally determined diffusion coefficient *D_ref_* of TMS, which is determined individually for each sample.

$$\eta=\frac{kT(1+0.695\left( \frac{r_{solv}}{r_{ref}} \right)^{2.234})}{6\pi D_{ref}r_{ref}}$$

After including all correction and calibration equations in the Stokes equation, the equation was rearranged for the hydrodynamic radii *r_H_*. The corresponding volumes *V_A_* were calculated with the assumption of a spherical shape.

$$D=\frac{kT(1+0.695\left( \frac{r_{solv}}{r_{H}} \right)^{2.234})}{6\pi\eta r_{H}}$$

The experimental self-diffusion coefficients *D_i_*, the viscosity corrected volumes *V_A_* and the error of all samples were determined. For consistency, all separated imine signals were used for integration.

Table S1: Measured self-diffusion coefficients and calculated volumes for **2a** at a concentration of 10 mM in CD_2_Cl_2_.

| Temperature/K | Diff coefficient/m^2^ s | Volume/Å^3^ | Error |
| --- | --- | --- | --- |
| 298 | 2.42e-09 | 256 | 9 |
| 283 | 1.18e-09 | 303 | 9 |

Table S2: Measured self-diffusion coefficients and calculated volumes for **1b/2a** at a concentration of 10 mM in CD_2_Cl_2_.

| Temperature/K | Diff coefficient/m^2^ s | Volume/Å^3^ | Error |
| --- | --- | --- | --- |
| 298 | 9.70e-10 | 2724 | 42 |
| 283 | 7.34e-10 | 2926 | 68 |
| 273 | 6.19e-10 | 3353 | 11 |
| 263 | 5.11e-10 | 4063 | 178 |
| 253 | 4.04e-10 | 4603 | 32 |

Table S3: Measured self-diffusion coefficients and calculated volumes for **1b/2b** at a concentration of 10 mM in CD_2_Cl_2_.

| Temperature/K | Diff coefficient/m^2^ s | Volume/Å^3^ | Error |
| --- | --- | --- | --- |
| 298 | 1.34e-09 | 1140 | 14 |
| 283 | 1.04e-09 | 1282 | 40 |
| 273 | 8.87e-10 | 1300 | 21 |
| 263 | 7.43e-10 | 1373 | 15 |
| 253 | 6.17e-10 | 1475 | 23 |

Table S4: Measured self-diffusion coefficients and calculated volumes for **1b/2c** at a concentration of 10 mM in CD_2_Cl_2_.

| Temperature/K | Diff coefficient/m^2^ s | Volume/Å^3^ | Error |
| --- | --- | --- | --- |
| 298 | 8.97e-10 | 989 | 2 |
| 283 | 6.88e-10 | 1037 | 0 |
| 273 | 5.78e-10 | 1063 | 50 |
| 263 | 4.81e-10 | 1154 | 14 |
| 253 | 3.90e-10 | 1240 | 3 |

# Non-linear Analysis

### Theory

We fitted the nonlinear reaction behavior to the simplest ML_2_ model, which was originally developed by Kagan.(*13, 14*) The model describes an asymmetric catalytic reaction based on two enantiomeric chiral ligands, where three different catalyst species may be formed: two homochiral and one *meso* complex with relative concentrations fixed by an equilibrium constant *K*.

*β* is a parameter which allows *K* to be related to the optical purity of the chiral catalyst *ee_cat_* by eliminating the individual mole fractions of the three complexes (*x*, *y* and *z*) from the equation.

$$K= \frac{z^{2}}{xy}$$

$$\beta=\frac{z}{x+y}=\frac{-K{ee}_{cat}^{2}+\sqrt{-4K{ee}_{cat}^{2}+K(4+K{ee}_{cat}^{2})}}{4+K{ee}_{cat}^{2}}$$

The two enantiopure catalysts ML*_R_*L*_R_* and ML*_S_*L*_S_* give identical reaction rates (*r_RR_* = *r_SS_*) and reaction products of opposite enantioselectivities (*ee_o_* and -*ee_o_*, respectively). Racemic product is formed from the *meso* catalyst ML*_R_*L*_S_*, which exhibits a reactivity of *g* relative to the enantiopure catalysts.

$$g=\frac{r_{RS}}{r_{RR}}$$

With these definitions, Kagan and co-workers showed that the enantioselectivity of the reaction product obtained from this mixture, *ee_prod_*, will vary with the *ee_cat_* according to the following equation:

$${ee}_{prod}={ee}_{0}{ee}_{cat}\frac{1+\beta}{1+g\beta}$$

The parameters *K* and *g* provide hints about the nature of the catalyst mixture at varying relative amount of L*_R_* and L*_S_*. A value of *K* = 4 gives a statistical distribution of ligands between the three complexes, where larger values indicate a predominance of the *meso* species. Values of *g* less than 1 indicate that the *meso* species is a less active catalyst than the enantiopure species for that particular reaction.

### Mukaiyama-Mannich Reaction Data

The Mukaiyama-Mannich reaction was performed following GP III using different ratios of the chiral Brønsted acid catalyst, yielding the corresponding imine with varying enantiomeric excess.

Table S5: Asymmetric Mukaiyama-Mannich reaction with aldimine **2a** and ketene silyl acetal catalyzed by CPA **1b** at – 78°C and 10% catalyst loading in toluene.

| *R*-1b/mmol | *S*-1b/mmol | *ee* (aux) | *ee*% (prod) |
| --- | --- | --- | --- |
| 0.02 | 0 | 1 | 95 |
| 0.018 | 0.002 | 0.8 | 92 |
| 0.016 | 0.004 | 0.6 | 86 |
| 0.014 | 0.006 | 0.4 | 76 |
| 0.012 | 0.008 | 0.2 | 42 |
| 0.01 | 0.01 | 0 | -1 |
| 0.008 | 0.012 | -0.2 | -47 |
| 0.006 | 0.014 | -0.4 | -77 |
| 0.004 | 0.016 | -0.6 | -90 |
| 0.002 | 0.018 | -0.8 | -93 |
| 0 | 0.02 | -1 | -96 |

Table S6: Asymmetric Mukaiyama-Mannich reaction with aldimine **2a** and ketene silyl acetal catalyzed by CPA **1b** at – 78°C and 10% catalyst loading in CD_2_Cl_2_.

| *R*-1b/mmol | *S*-1b/mmol | *ee* (aux) | *ee*% (prod) |
| --- | --- | --- | --- |
| 0.02 | 0 | 1 | 81 |
| 0.018 | 0.002 | 0.8 | 73 |
| 0.016 | 0.004 | 0.6 | 57 |
| 0.014 | 0.006 | 0.4 | 41 |
| 0.012 | 0.008 | 0.2 | 22 |
| 0.01 | 0.01 | 0 | 0 |
| 0.008 | 0.012 | -0.2 | -26 |
| 0.006 | 0.014 | -0.4 | -48 |
| 0.004 | 0.016 | -0.6 | -66 |
| 0.002 | 0.018 | -0.8 | -75 |
| 0 | 0.02 | -1 | -80 |

Table S7: Asymmetric Mukaiyama-Mannich reaction with aldimine **2c** and ketene silyl acetal catalyzed by CPA **1b** at – 78°C and 10% catalyst loading in toluene.

| *R*-1b/mmol | *S*-1b/mmol | *ee* (aux) | *ee*% (prod) |
| --- | --- | --- | --- |
| 0.02 | 0 | 1 | 72 |
| 0.018 | 0.002 | 0.8 | 56 |
| 0.016 | 0.004 | 0.6 | 44 |
| 0.014 | 0.006 | 0.4 | 29 |
| 0.012 | 0.008 | 0.2 | 15 |
| 0.01 | 0.01 | 0 | -1 |

Table S8: Asymmetric Mukaiyama-Mannich reaction with aldimine **2a** and ketene silyl acetal catalyzed by NTPA **1c** at – 78°C and 10% catalyst loading in toluene.

| *R*-1b/mmol | *S*-1b/mmol | *ee* (aux) | *ee*% (prod) |
| --- | --- | --- | --- |
| 0.02 | 0 | 1 | 43 |
| 0.018 | 0.002 | 0.8 | 31 |
| 0.016 | 0.004 | 0.6 | 25 |
| 0.014 | 0.006 | 0.4 | 16 |
| 0.012 | 0.008 | 0.2 | 6 |
| 0.01 | 0.01 | 0 | 0 |

### Data Fitting

Figure S43: Asymmetric Mukaiyama-Mannich reaction with aldimine **2a** and ketene silyl acetal catalyzed by CPA **1b** at – 78°C and 10% catalyst loading in toluene.

Figure S44: Asymmetric Mukaiyama-Mannich reaction with aldimine **2a** and ketene silyl acetal catalyzed by CPA **1b** at – 78°C and 10% catalyst loading in CD_2_Cl_2_.

Figure S45: Asymmetric Mukaiyama-Mannich reaction with aldimine **2c** and ketene silyl acetal catalyzed by CPA **1b** at – 78°C and 10% catalyst loading in toluene.

Figure S46: Asymmetric Mukaiyama-Mannich reaction with aldimine **2a** and ketene silyl acetal catalyzed by NTPA **1c** at – 78°C and 10% catalyst loading in toluene.

# Reaction Kinetics of the Mukaiyama-Mannich reaction

### Procedure for the ex-situ kinetics

General procedure for ex-situ kinetics (GP IV):

To five different solutions of aldimine **2a** (0.1 mmol) and 1,3,5-Trimethoxybenzene (0.1 mmol) was added the chiral Brønsted acid catalyst **1b** in varying concentrations (0.001 – 0.025 mmol) in 1mL dry CD_2_Cl_2_ and cooled to -78 °C. A solution of ketene silyl acetal (34.9 mg, 40.6 μL, 0.2 mmol) under argon atmosphere was then added. At 10 min intervals, 100 μL aliquots of the reaction mixture were transferred to an NMR tube, quenched in a warm water bath, diluted by 400 μL of dry CD_2_Cl_2_ and analyzed by ^1^H NMR (relaxation delay d1 = 20 s) to determine the yield. The absence of reaction at room temperature was confirmed by remeasuring the first sample taken after 10 min; identical yields were obtained after 1 h. The first 100 μL aliquot was taken prior to the addition of the silyl acetal to determine the exact imine-to-catalyst ratio. Catalyst concentrations of 0.7mM, 4.5 mM, 9.8 mM, 20.3 mM, and 25.8 mM were measured. Yields were plotted against time, and initial reaction rates *v_0_* were determined from the slope of the linear plots (Figure S47). The following initial rates were obtained:

Table S9: Initial rates for varying catalyst concentrations in the Asymmetric Mukaiyama-Mannich reaction with aldimine **2a** catalyzed by CPA **1b** at – 78°C.

| **Catalyst concentration [mM]** | **v_o_ [min^-1^]** |
| --- | --- |
| 0.7 | No reaction |
| 4.5 | 0.23 |
| 9.8 | 0.60 |
| 20.3 | 0.75 |
| 25.8 | 0.76 |

Figure S47: The linear ranges of the reaction profiles for the Mukaiyama-Mannich reaction of imine **2a** are shown. The kinetics were done ex-situ with catalyst **1b** (4.5mM, 9.8 mM 20.3 mM and 25.8 mM) at -78 °C in dry CD_2_Cl_2_.

$$v=\frac{\partial\left[ amine \right]}{\partial t}=k_{obs} \left[ Cat \right]^{n} \left[ Imine \right]^{m} \left[ Silyl \right]^{l}$$

The reaction order *n* can be calculated using the following equation:

$$n=\frac{\ln\left( \frac{v_{0,2}}{v_{0,1}} \right)}{\ln\left( \frac{\left[ cat \right]_{2}}{\left[ cat \right]_{1}} \right)}$$

The following reaction orders *n* were obtained:

Table S10: Catalyst Orders in the Asymmetric Mukaiyama-Mannich reaction with aldimine **2a** catalyzed by CPA **1b** at – 78°C.

| **Catalyst Concentration Range [mM]** | ***n*** |
| --- | --- |
| 4.5 – 9.8 | 1.26 |
| 9.8 – 20.3 | 0.31 |
| 20.3 – 25.8 | 0.046 |

**Interpretation:**

To analyze the reaction kinetics, initial rates were measured across a range of catalyst concentrations. The determined catalyst order varied from 1.26 at low concentrations to ~ 0 at higher concentrations. These observations lead to the following conclusions based on the different concentration regimes:

(I) Low-catalyst concentration regime: A kinetic order in catalyst greater than 1 was observed, specifically 1.26 when increasing the catalyst concentration from 4.5mM to9.8mM. Notably, upon reducing the catalyst concentration to 0.7mM, no product formation was detected. Generally, reactions involving two catalyst molecules are associated with a two-order dependence. While catalyst aggregation can shift the resting state from the monomeric to the dimeric species, thereby reducing the dependence of the reaction rate on the catalyst, this dimerization alone cannot account for the complete lack of reaction at 0.7mM. Furthermore, the dimerization is likely not dominant enough at 10mM to reduce the kinetic order from 2 to 1. Therefore, the CPA-catalyzed hydrolysis of the silyl acetal must be considered. Assuming a first-order dependence on CPA for the decomposition of the silyl acetal, the side reaction outcompetes the main reaction pathway at sufficiently low CPA concentrations, effectively halting product formation.

(II) High-catalyst concentration regime: Increasing the CPA concentration shifts resting state toward the dimeric catalytic species. However, as derived in the low-catalyst regime, this shift only reduces the catalyst order from 2 to 1. An apparent kinetic order in catalyst approaching zero can only be rationalized by a partial inactivation of the catalyst. Since the catalyst was fully dissolved, this reduction is proposed to be due to the inherent presence of water. Even with rigorous drying, CPAs typically contain some water. Consequently, as the catalyst concentration was increased, the absolute amount of water in the system also increased, raising the apparent rate constant of the silyl acetal hydrolysis. The subsequent consumption of the silyl acetal and the competitive blocking of the catalytic sites the silyl acetal result in the observed trend toward zero-order kinetics at high concentrations.

Though, the determined kinetic order of 1.26 at low-catalyst concentrations indicates the active participation of a dimeric catalyst species. In conclusion, the kinetic study is fully consistent with the NLE analysis.(*15*)

# Computational Details

### General Molecular Dynamics (MD) Simulations Details

All MD simulations were conducted with GROMACS(*16*), version 2025.2. During the simulations a velocity rescale thermostat(*17*) (tau = 1.0 ps) was used to set the target temperature. A reference pressure of 1 bar was set by the c-rescale barostat(*18*) (compressibility = 2.5^-5^ bar^-1^). The verlet cutoff scheme with a cutoff of 1.0 nm as well as PME for coulomb interactions were used. A dispersion correction for energy and pressure was applied.

### Replica Exchange Molecular Dynamics Simulations Details

All 62 replicas were equilibrated for 1 ns with a timestep of 1 fs. After that every replica was simulated for 275 ns with the same timestep. During the simulation an average exchange probability of 24.0 % was achieved, the exchanges were proposed every 5000 steps with left/right alternation. The simulation boxes contained 2 CPA, 2 Iminium-Ions and 1705 CD_2_Cl_2_ Molecules. The individual temperatures were as follows:

190.0, 192.5, 195.0, 197.5, 200.0, 203.0, 206.0, 209.0, 212.0, 215.0, 218.0, 221.0, 224.0, 227.0, 230.0, 233.5, 237.0, 240.5, 244.0, 247.5, 251.0, 254.5, 258.0, 262.0, 266.0, 270.0, 274.0, 278.0, 282.0, 286.0, 290.0, 294.0, 298.0, 303.0, 308.0, 313.0, 318.0, 323.0, 328.0, 333.0, 338.0, 343.0, 348.0, 353.0, 358.0, 363.0, 368.0, 373.0, 378.0, 383.0, 388.0, 393.0, 398.0, 403.0, 408.0, 413.0, 418.0, 423.0, 428.0, 433.0, 438.0, 443.0.

The average exchange probability for every individual pair of replicas can be found in Figure S48.

Figure S48: Average Exchange Probabilities between Replicas

### Results and Statistics

In order to investigate the nature and properties of occurring H-bonds, the distribution of angles and bond lengths of all hydrogen bonds between CPA and the Iminium-Ions were analyzed, which can be seen in Figure S49.

Figure S49: 2D-Distribution (angles, distances) of Hydrogen Bonds between CPA and Iminium-Ions in CD_2_Cl_2_. Borders drawn at 2.4 Angstroms and 115 degrees.

In order to look for occurring conformers within the simulations, the N-N distance between the two Iminium Ions in the boxes was investigated as a starting point to narrowing down the exact number and type of the present conformers. As can be seen in Figure S50, there are three maxima present, which leads to the conclusion that there could be potentially three or more conformers to be found. This is consistent with the findings in the NMR spectra so far.

Figure S50: Distance between the two Nitrogen atoms of the two iminium ions.

As a general assumption, we estimated the total possible number of conformers regarding two CPA and two Iminium Ions to 9. Since we have two Iminium Ions which both have two H-bond sites (O-H and N-H) and two CPA molecules which also have two H-bonding sites, one Iminium Ion can either coordinate bridging between two CPAs, coordinate one CPA with two of its sites (c2O) or coordinate one CPA with one of its sites (c1O). For two Iminium Ions that can coordinate independently this means nine possibilities. Those nine possibilities along with their absolute occurrence is depicted in Figure S51.

Figure S51: Counts of nine different possible structural conformers of the CPA-Iminium dimer.

After reviewing all the above possibilities, several groups could be formed:

**Group 1**: Monomers are spatially close, but neither bridging, nor coordinating towards each other. This includes four of the nine cases (c2O-c2O, c1O-c1O, c1O-c2O, c2O-c1O). This group could easily be identified to belong to the broad peak in Figure S50 at 28 Å.

**Group 2**: The two Iminium Ions are bridging between the two CPAs and are all together forming a dimer. This naturally includes only one of the nine cases (bridging-bridging) and could be identified to have an N-N distance of 4 Å, which correlates with the left peak in Figure S50.

**Group 3**: One of the Iminium Ions is bridging the other one is either in c2O or c1O state. This case could be linked with the remaining four cases (bridging-c2O, bridging-c1O and vice versa). This group can be linked to the peak at 10 Å.

Under group three several structures have been found that share π-Stacking as a present coordination feature besides H-bonding, as can be seen in Figure S55. To further investigate the nature of the dimer conformation of Group 2, the occurrence of four possible structure motifs has been counted, which is represented in Figure S52. The four possible structures revolve around the Iminium Ions being ordered anti/syn concerning their methoxy group or being ordered cis/trans concerning their alcohol group.

Figure S52: Counts of different possible structure of the bridging dimer.

There it is possible to observe that the cis-anti confirmation is the one that occurs most, while cis-syn almost never occurs, trans-anti never occurs and trans-syn only rarely occurs. In conclusion and based on this MD-study it is highly probable that the three dimeric peaks in the NMR can be assigned to the anti-cis (Figure S53) and syn-trans (Figure S54) bridging dimers and to the case where one of the Iminium Ions is bridging while the other one is coordinating only one of the CPA molecules (Figure S55).

Figure S53: Dimer Structure Motif: anti-cis

Figure S54: Dimer Structure Motif: syn-trans

Figure S55: Dimer Structure Motif: bridging-c2O

### Calculation of the Substrate Accessible Surface Area and Prediction of Product Isomerism

The substrate-accessible surface area (SuASA) of the iminium carbon atoms was calculated using FreeSASA (version 2.2.1).(*19*) A probe radius of 3 Å was employed along with the Shrake Rupley Algorithm (*20*) using 2000 grid points per atom. Given that every [CPA/imine]_2_ dimer contains two iminium ions that can potentially be reaction sites, SuASA values were calculated separately for each iminium carbon atom.

For the open conformer (bridging-c2o) (56), the anti-cis conformer (61), and the syn-trans conformer (15), all snapshots in which these structures occurred were extracted from the molecular dynamics trajectories and analyzed. For each structure the iminium carbon atom was defined as reactive when its SuASA value was greater than the SuASA value of the corresponding carbon atom of the second iminium ion. Structures in which both carbon atoms showed values of 0 Å²were excluded from further analysis and classified as nonreactive.

For each reactive carbon atom, a plane was constructed using the C-N and C-C bond vectors to define the iminium π-system. The corresponding normal vector was calculated to distinguish between the two faces of the iminium ion. Both sides were then screened for neighboring atoms within a 6 Å²radius. The side with fewer or no neighbors was then assigned as the preferred direction of the nucleophilic attack.

That information enabled us to qualitatively predict the product enantiomer ratios for the three conformational motifs. The syn-trans conformer (Figure S54) shows a SuASA of 0 Å² for all but one structures which exhibited a single carbon atom with a SuASA of 2.8 Å². The predicted product for this conformer was the S enantiomer.

For the anti-cis conformer (Figure S53), 33 frames satisfied the accessibility criterion, predicting an S:R ratio of 24:9. Average SuASA values were 4.6 Å² for the S-forming structures and 1.5 Å² for the R-forming structures.

The bridging-c2o conformer (Figure S55 and Figure S57) yielded 48 qualifying frames, corresponding to a predicted S:R ratio of 32:16, with average SuASA values of 4.3 Å² (S-forming) and 6.5 Å²(R-forming).

Overall, the bridging-c2o structure exhibits the highest iminium accessibility and consequently the greatest propensity for a substrate attack. Furthermore, across all analyzed replica exchange molecular dynamics (REMD) frames, the exposed face of the more accessible iminium ion within this conformer consistently corresponds to S-product formation. These values are summarized in Table S11.

To account for relative population effects, SuASA values were weighted by their relative occurrence to ${SuASA}_{weighted}=SuASA\cdot\frac{N_{i}}{N_{total}}$, with $N_{total}=132$. This scaling yields a weighted parameter that qualitatively correlates with the contribution to the reactive complex and can now be compared across all structure motifs (Table S12). The weighted analysis further supports that the bridging-c2o dimer is the dominant contributor to the reactive complex and favors formation of the S product.

To further visualize the accessibility of the reactive sites within the open dimeric complex, Figure S56 shows the Van der Waals radii of the two CPA molecules and two iminium ions (orange and purple), along with blue spheres indicating the positions a substrate would need to enable nucleophilic attack. It is important to note that the spheres have a radius of approximately 3 Å, which is exactly the probe radius of the SuASA algorithm and still smaller than the actual substrate size. However, it was chosen to guarantee the best possible visibility of all molecules. It is clearly visible that only one iminium ion is sterically accessible. Furthermore, only one side of the mentioned iminium ion is accessible, at least in this one example snapshot, which directly correlates to the results of the SuASA analysis. Below that in Figure S57, we show the same complex without the van der Waals radii.

Table S11: SuASA values in Å² for the three complexes with number of occurrences in brackets.

| **SASA** | **Bridging-c2o  (occurrence)** | **Anti-Cis (occurrence)** | **Syn-Trans (occurrence)** |
| --- | --- | --- | --- |
| **Average (both C atoms)** | 2.4 (56) | 1.1 (61) | 0.1 (15) |
| **Only free C** | 5.0 (48) | 3.8 (33) | 2.8 (1) |
| **Only hindered C** | 0.5 (48) | 0.1 (33) | 0.0 (1) |
| **Only free C, S product** | 4.3 (32) | 4.6 (24) | 2.8 (1) |
| **Only free C, R product** | 6.5 (16) | 1.5 (9) | none |

Table S12: Relative contribution of the three structures to the reactive complex. Derived from the SuASA values (in Å²) weighted by their relative occurrence during REMD.

| **SuASA** | **Bridging-c2o** | **Anti-Cis** | **Syn-Trans** |
| --- | --- | --- | --- |
| **Average (both C atoms)** | 1.0 | 0.5 | 0.0 |
| **Only free C** | 1.8 | 1.0 | 0.0 |
| **Only hindered C** | 0.0 | 0.0 | 0.0 |
| **Only free C, S product** | 1.0 | 0.8 | 0.0 |
| **Only free C, R product** | 0.8 | 0.1 | none |

Figure S56: View of structural motif III illustrating the steric accessibility of the bridging conformer.

Figure S57: View of structural motif III illustrating the direction of attack by the silyl acetal.

### Force Field and Force Field Generation Details

The dichlormethane force field was created by Horinek et al.(*21*) In that force field the Lennard-Jones paramters of the Cl atom were slightly adjusted (s = 0.33 nm, e = 1.2 kJ/mol) to reproduce the correct density at 300 K. The CPA forcefield was prepared with antechamber (v.22.0)(*22*) and acpype (v. 2023.10.27)(*23*) based on the General Amber Force Field 2 (GAFF2).(*24*) Geometry optimizations and single point energy calculations were done with ORCA (v.6.0.1).(*25*) RESP charges were calculated with Multiwfn (v. 3.7).(*26*) The charges in the CPA topology were altered to the RESP charges. The Iminium ion force field was prepared with QUBEKIT (v. 2.1.1).(*27–29*) The ORCA calculations concerning the CPA molecule utilized the PBE0 functional and def-TZVPP basis set and Grimme's D3 dispersion Correction with Becke-Johnson damping. The QUBEKIT calculations concerning the iminium ion utilized the *ω*b97x-d functional with the6-311++G(d,p) basis set.

**References**

1. Gramüller, J.; Dullinger, P.; Horinek, D.; Gschwind, R. M. Bidentate substrate binding in Brønsted acid catalysis: structural space, hydrogen bonding and dimerization. *Chem. Sci.* **2022,** *13* (48), 14366–14372. DOI: 10.1039/D2SC05076E.

2. Ciaccia, M.; Tosi, I.; Baldini, L.; Cacciapaglia, R.; Mandolini, L.; Di Stefano, S.; Hunter, C. A. Applications of dynamic combinatorial chemistry for the determination of effective molarity. *Chem. Sci.* **2015,** *6* (1), 144–151. DOI: 10.1039/C4SC02347A.

3. Qin, X.; Ding, G.; Wang, Z.; Zhang, S.; Li, H.; Luo, Z.; Gao, F. Remarkable difference between five- and six- number-membered ring transition states for intramolecular proton transfer in excited state. *J. Photochem. Photobiol., A* **2017,** *339*, 25–35. DOI: 10.1016/j.jphotochem.2017.02.016.

4. Cao, C.-T.; Zhou, W.; Cao, C. Abnormal effect of hydroxyl on the longest wavelength maximum in ultraviolet absorption spectra for bis-aryl Schiff bases. *J. Phys. Org. Chem.* **2017,** *30* (10), e3672. DOI: 10.1002/poc.3672.

5. Zhou, F.; Yamamoto, H. A Powerful Chiral Phosphoric Acid Catalyst for Enantioselective Mukaiyama-Mannich Reactions. *Angew. Chem. Int. Ed.* **2016,** *55* (31), 8970–8974. DOI: 10.1002/anie.201603929.

6. Hecht, M.; Dullinger, P.; Silva, W.; Horinek, D.; Gschwind, R. M. Highly acidic N-triflylphosphoramides as chiral Brønsted acid catalysts: the effect of weak hydrogen bonds and multiple acceptors on complex structures and aggregation. *Chem. Sci.* **2024,** *15* (24), 9104–9111. DOI: 10.1039/D4SC01939C.

7. Jerschow, A.; Müller, N. Diffusion-Separated Nuclear Magnetic Resonance Spectroscopy of Polymer Mixtures. *Macromolecules* **1998,** *31* (19), 6573–6578. DOI: 10.1021/ma9801772.

8. Stejskal, E. O.; Tanner, J. E. Spin Diffusion Measurements: Spin Echoes in the Presence of a Time-Dependent Field Gradient. *The Journal of Chemical Physics* **1965,** *42* (1), 288–292. DOI: 10.1063/1.1695690.

9. Macchioni, A.; Ciancaleoni, G.; Zuccaccia, C.; Zuccaccia, D. Determining accurate molecular sizes in solution through NMR diffusion spectroscopy. *Chem. Soc. Rev.* **2008,** *37* (3), 479–489. DOI: 10.1039/b615067p.

10. Zuccaccia, D.; Macchioni, A. An Accurate Methodology to Identify the Level of Aggregation in Solution by PGSE NMR Measurements: The Case of Half-Sandwich Diamino Ruthenium(II) Salts. *Organometallics* **2005,** *24* (14), 3476–3486. DOI: 10.1021/om050145k.

11. Chen, H. C.; Chen, S. H. Diffusion of crown ethers in alcohols. *J. Phys. Chem.* **1984,** *88* (21), 5118–5121. DOI: 10.1021/j150665a063.

12. Ben-Amotz, D.; Willis, K. G. Molecular hard-sphere volume increments. *J. Phys. Chem.* **1993,** *97* (29), 7736–7742. DOI: 10.1021/j100131a051.

13. Puchot, C.; Samuel, O.; Dunach, E.; Zhao, S.; Agami, C.; Kagan, H. B. Nonlinear effects in asymmetric synthesis. Examples in asymmetric oxidations and aldolization reactions. *Journal of the American Chemical Society* **1986,** *108* (9), 2353–2357. DOI: 10.1021/ja00269a036.

14. Guillaneux, D.; Zhao, S.-H.; Samuel, O.; Rainford, D.; Kagan, H. B. Nonlinear Effects in Asymmetric Catalysis. *Journal of the American Chemical Society* **1994,** *116* (21), 9430–9439. DOI: 10.1021/ja00100a004.

15. Blackmond, D. G. Kinetic aspects of nonlinear effects in asymmetric catalysis. *Acc. Chem. Res.* **2000,** *33* (6), 402–411. DOI: 10.1021/ar990083s.

16. Abraham, M. J.; Murtola, T.; Schulz, R.; Páll, S.; Smith, J. C.; Hess, B.; Lindahl, E. GROMACS: High performance molecular simulations through multi-level parallelism from laptops to supercomputers. *SoftwareX* **2015,** *1-2*, 19–25. DOI: 10.1016/j.softx.2015.06.001.

17. Bussi, G.; Donadio, D.; Parrinello, M. Canonical sampling through velocity rescaling. *The Journal of Chemical Physics* **2007,** *126* (1), 14101. DOI: 10.1063/1.2408420.

18. Bernetti, M.; Bussi, G. Pressure control using stochastic cell rescaling. *The Journal of Chemical Physics* **2020,** *153* (11), 114107. DOI: 10.1063/5.0020514.

19. Mitternacht, S. FreeSASA: An open source C library for solvent accessible surface area calculations. *F1000Research* **2016,** *5*, 189. DOI: 10.12688/f1000research.7931.1.

20. Shrake, A.; Rupley, J. A. Environment and exposure to solvent of protein atoms. Lysozyme and insulin. *J. Mol. Biol.* **1973,** *79* (2), 351–371. DOI: 10.1016/0022-2836(73)90011-9.

21. Berg, N.; Bergwinkl, S.; Nuernberger, P.; Horinek, D.; Gschwind, R. M. Extended Hydrogen Bond Networks for Effective Proton-Coupled Electron Transfer (PCET) Reactions: The Unexpected Role of Thiophenol and Its Acidic Channel in Photocatalytic Hydroamidations. *J. Am. Chem. Soc.* **2021,** *143* (2), 724–735. DOI: 10.1021/jacs.0c08673.

22. Wang, J.; Wang, W.; Kollman, P. A.; Case, D. A. Automatic atom type and bond type perception in molecular mechanical calculations. *Journal of molecular graphics & modelling* **2006,** *25* (2), 247–260. DOI: 10.1016/j.jmgm.2005.12.005.

23. Da Sousa Silva, A. W.; Vranken, W. F. ACPYPE - AnteChamber PYthon Parser interfacE. *BMC research notes* **2012,** *5*, 367. DOI: 10.1186/1756-0500-5-367.

24. Wang, J.; Wolf, R. M.; Caldwell, J. W.; Kollman, P. A.; Case, D. A. Development and testing of a general amber force field. *J. Comput. Chem.* **2004,** *25* (9), 1157–1174. DOI: 10.1002/jcc.20035.

25. Neese, F. The ORCA program system. *WIREs Comput Mol Sci* **2012,** *2* (1), 73–78. DOI: 10.1002/wcms.81.

26. Lu, T. A comprehensive electron wavefunction analysis toolbox for chemists, Multiwfn. *The Journal of Chemical Physics* **2024,** *161* (8). DOI: 10.1063/5.0216272.

27. Horton, J. T.; Allen, A. E. A.; Dodda, L. S.; Cole, D. J. QUBEKit: Automating the Derivation of Force Field Parameters from Quantum Mechanics. *J. Chem. Inf. Model.* **2019,** *59* (4), 1366–1381. DOI: 10.1021/acs.jcim.8b00767.

28. Cole, D. J.; Vilseck, J. Z.; Tirado-Rives, J.; Payne, M. C.; Jorgensen, W. L. Biomolecular Force Field Parameterization via Atoms-in-Molecule Electron Density Partitioning. *J. Chem. Theory Comput.* **2016,** *12* (5), 2312–2323. DOI: 10.1021/acs.jctc.6b00027.

29. Allen, A. E. A.; Payne, M. C.; Cole, D. J. Harmonic Force Constants for Molecular Mechanics Force Fields via Hessian Matrix Projection. *J. Chem. Theory Comput.* **2018,** *14* (1), 274–281. DOI: 10.1021/acs.jctc.7b00785.
